# A wearable cardiac ultrasound imager

**DOI:** 10.1038/s41586-022-05498-z

**Published:** 2023-01-25

**Authors:** Hongjie Hu, Hao Huang, Mohan Li, Xiaoxiang Gao, Lu Yin, Ruixiang Qi, Ray S. Wu, Xiangjun Chen, Yuxiang Ma, Keren Shi, Chenghai Li, Timothy M. Maus, Brady Huang, Chengchangfeng Lu, Muyang Lin, Sai Zhou, Zhiyuan Lou, Yue Gu, Yimu Chen, Yusheng Lei, Xinyu Wang, Ruotao Wang, Wentong Yue, Xinyi Yang, Yizhou Bian, Jing Mu, Geonho Park, Shu Xiang, Shengqiang Cai, Paul W. Corey, Joseph Wang, Sheng Xu

**Affiliations:** 1 Department of Nanoengineering, University of California San Diego, La Jolla, CA USA; 2 Department of Electrical and Computer Engineering, University of California San Diego, La Jolla, CA USA; 3 Department of Computer Science and Engineering, University of California San Diego, La Jolla, CA USA; 4 Materials Science and Engineering Program, University of California San Diego, La Jolla, CA USA; 5 Department of Mechanical Engineering, Massachusetts Institute of Technology, Cambridge, MA USA; 6 Materials Science and Engineering Program, University of California, Riverside, CA USA; 7 Department of Mechanical and Aerospace Engineering, University of California San Diego, La Jolla, CA USA; 8 Department of Anesthesiology, University of California, San Diego Health Sulpizio Cardiovascular Center, La Jolla, CA USA; 9 Department of Radiology, School of Medicine, University of California San Diego, La Jolla, CA USA; 10 Department of Neurosurgery, Yale University, New Haven, CT USA; 11 Department of Chemical Engineering, Stanford University, Stanford, CA USA; 12 Softsonics, Inc., San Diego, CA USA; 13 Department of Anesthesiology, Sharp Memorial Hospital, San Diego, CA USA; 14 Department of Bioengineering, University of California San Diego, La Jolla, CA USA

**Keywords:** Echocardiography, Disease prevention, Sensors and biosensors

## Abstract

Continuous imaging of cardiac functions is highly desirable for the assessment of long-term cardiovascular health, detection of acute cardiac dysfunction and clinical management of critically ill or surgical patients^1–4^. However, conventional non-invasive approaches to image the cardiac function cannot provide continuous measurements owing to device bulkiness^5–11^, and existing wearable cardiac devices can only capture signals on the skin^12–16^. Here we report a wearable ultrasonic device for continuous, real-time and direct cardiac function assessment. We introduce innovations in device design and material fabrication that improve the mechanical coupling between the device and human skin, allowing the left ventricle to be examined from different views during motion. We also develop a deep learning model that automatically extracts the left ventricular volume from the continuous image recording, yielding waveforms of key cardiac performance indices such as stroke volume, cardiac output and ejection fraction. This technology enables dynamic wearable monitoring of cardiac performance with substantially improved accuracy in various environments.

## Abstract (web summary)

Innovations in device design, material fabrication and deep learning are described, leading to a wearable ultrasound transducer capable of dynamic cardiac imaging in various environments and under different conditions.

## Main

## Main

**Fig. 1:**
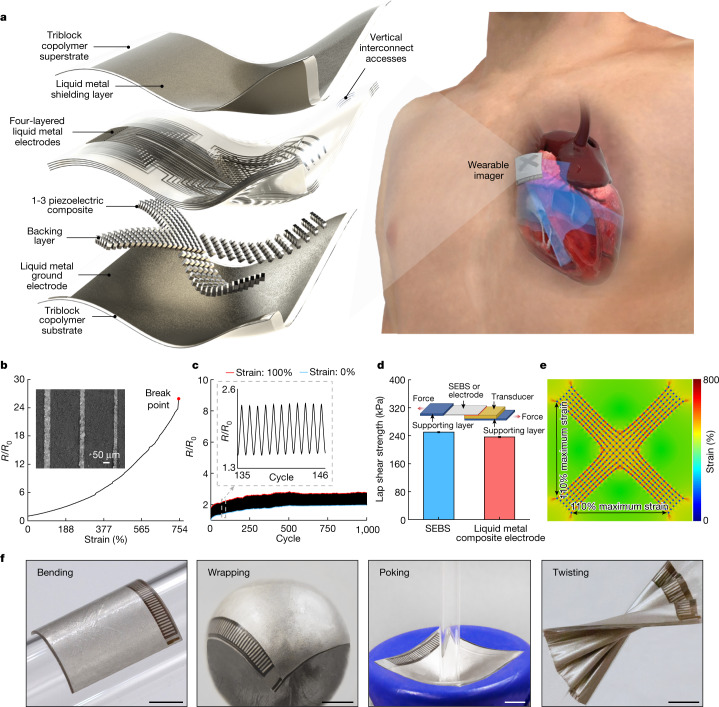
Design and characterization of the wearable cardiac imager. **a**, Schematics showing the exploded view of the wearable imager, with key components labelled (left) and its working principle (right). **b**, Resistance of the liquid metal composite electrode as a function of uniaxial tensile strain. The electrode can be stretched up to about 750% without failure. The *y* axis is the relative resistance defined as *R*/*R*_0_, in which *R*_0_ and *R* are the measured resistances at 0% strain and a given strain, respectively. The inset is a scanning electron micrograph of the liquid metal composite electrodes with a width as small as about 30 µm. Scale bar, 50 μm. **c**, Cycling performance of the electrode between 0% and 100% uniaxial tensile strain, showing the robustness of the electrode. The inset shows the zoomed-in features of the graph during cyclic stretching and relaxing of the electrode. **d**, Lap shear strength of the bonding between transducer elements and SEBS or liquid metal composite electrode. Data are mean and s.d. from *n* = 3 tests. The inset is a schematic setup of the lap shear test. **e**, Finite element analysis of the entire device under 110% biaxial stretching. **f**, Optical images showing the mechanical compliance of the wearable imager when bent on a developable surface, wrapped around a non-developable surface, poked and twisted. Scale bars, 5 mm.

**Extended Data Fig. 1:**
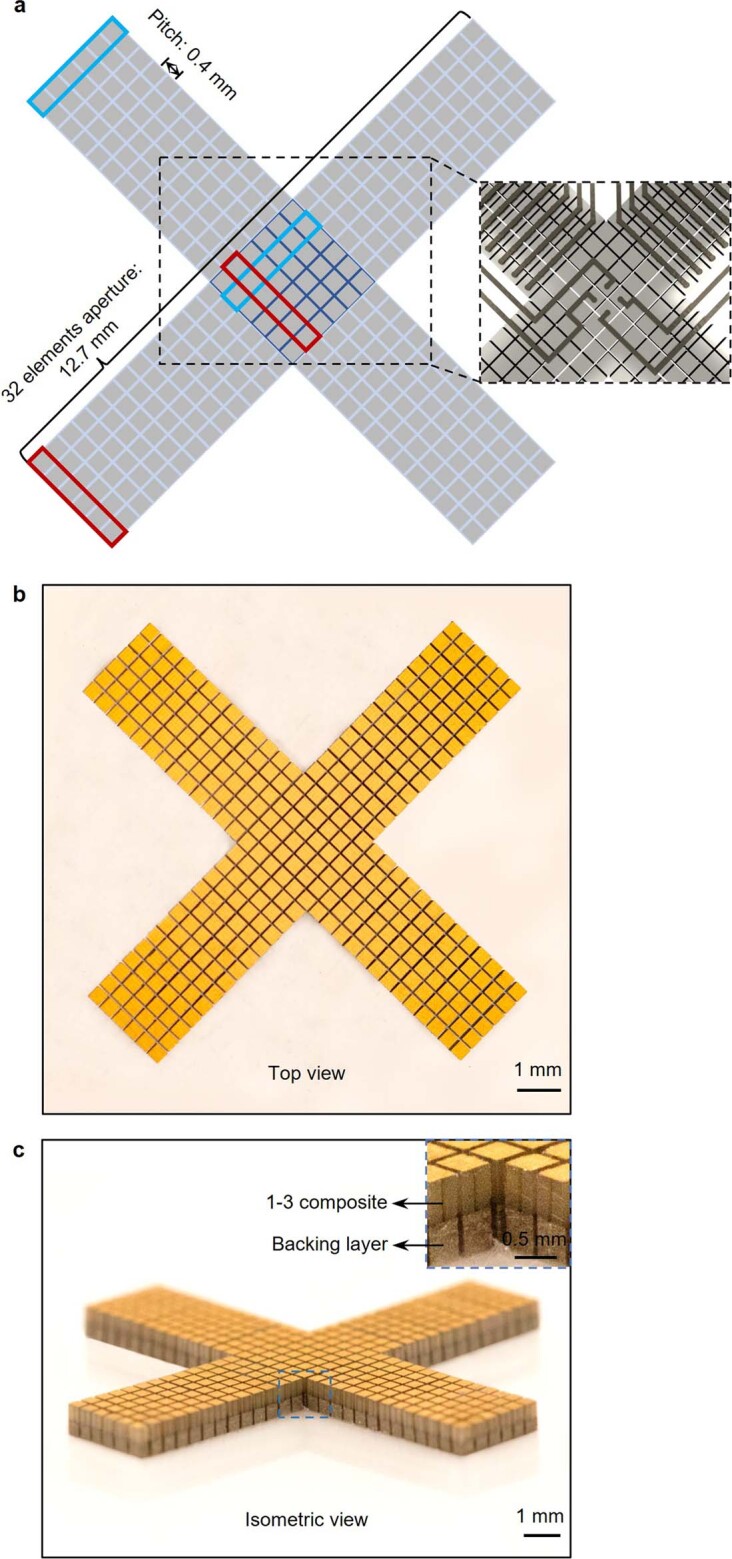
Schematics and optical images of the orthogonal imager. **a**, The orthogonal imager consists of four arms, in which six small elements in one column are combined as one long element, and a central part that is shared by the four arms. The blue and red boxes label a long element integrated by six small pieces in each direction. The number of elements in one direction is 32. The pitch between the elements is 0.4 mm. Optical images in top view (**b**) and isometric view (**c**) showing the morphology of the orthogonal array. We used an automatic alignment strategy to fabricate the orthogonal array by bonding a large piece of backing layer with a large piece of 1-3 composite and then dicing them together into small elements with designed configurations. Inset in **c** shows the details of the elements. The 1-3 composite and backing layer have been labelled.

The device features piezoelectric transducer arrays, liquid metal composite electrodes and triblock copolymer encapsulation, as shown by the exploded schematics (Fig. 1a, left, Extended Data Fig. 1 and Supplementary Discussion 3). The device is built on styrene–ethylene–butylene–styrene (SEBS). To provide a comprehensive view of the heart, standard clinical practice is to image it in two orthogonal orientations by rotating the ultrasound probe^17^. To eliminate the need for manual rotation, we designed the device with an orthogonal configuration (Fig. 1a, right and Supplementary Videos 1 and 2). Each transducer element consisted of an anisotropic 1-3 piezoelectric composite and a silver-epoxy-based backing layer^18,19^. To balance the penetration depth and spatial resolution, we chose a centre resonant frequency of 3 MHz for deep tissue imaging^19^ (Supplementary Fig. 1). The array pitch was 0.4 mm (that is, 0.78 ultrasonic wavelengths), which enhances lateral resolutions and reduces grating lobes^20^.

To individually address each element in such a compact array, we made high-density multilayered stretchable electrodes based on a composite of eutectic gallium–indium liquid metal and SEBS^21^. The composite is highly conductive and easy to pattern (Fig. 1b,c, Supplementary Figs. 2–4 and Methods). Lap shear measurements show that the interfacial bonding strength is about 250 kPa between the transducer element and the SEBS substrate, and about 236 kPa between the transducer element and the composite electrode (Fig. 1d and Supplementary Fig. 5), which are both stronger than typical commercial adhesives^22^ (Supplementary Table 2). The resulting electrode has a thickness of only about 8 μm (Supplementary Figs. 6 and 7). Electromagnetic shielding, also made of the composite, can mitigate the interference of ambient electromagnetic waves, which reduces the noise in the ultrasound radiofrequency signals and enhances the image quality^23^ (Supplementary Fig. 8 and Supplementary Discussion 4). The device has excellent electromechanical properties, as determined by its high electromechanical coupling coefficient, low dielectric loss, wide bandwidth and negligible crosstalk (Supplementary Fig. 1 and Methods). The entire device has a low Young’s modulus of 921 kPa, comparable with the human skin modulus^24^ (Supplementary Fig. 9). The device exhibits a high stretchability of up to approximately 110% (Fig. 1e and Supplementary Fig. 10) and can withstand various deformations (Fig. 1f). Considering that the typical strain on the human skin is within 20% (ref. ^19^), these mechanical properties allow the wearable imager to maintain intimate contact with the skin over a large area, which is challenging for rigid ultrasound devices^25^.

## Imaging strategies and characterizations

We evaluated the quality of the generated images based on the five most crucial metrics for anatomical imaging: spatial resolutions (axial, lateral and elevational), signal-to-noise ratio, location accuracies (axial and lateral), dynamic range and contrast-to-noise-ratio^26^.

**Fig. 2:**
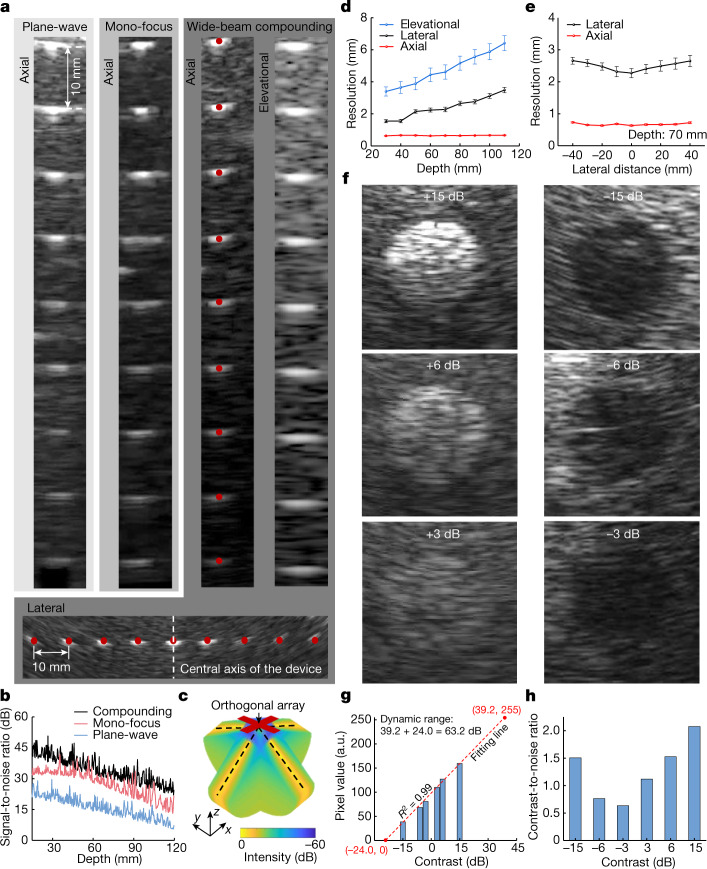
B-mode imaging strategies and characterizations. **a**, Imaging results on wire (100 µm in diameter) phantoms using different transmit beamforming strategies. The first three columns show the images through plane-wave, mono-focus and wide-beam compounding at different depths, respectively. The fourth column shows the imaging resolution of wide-beam compounding in the elevational direction. The bottom row shows images of laterally distributed wires by the wide-beam compounding, from which the lateral accuracy and spatial resolutions at different lateral distances from the central axis can be obtained. **b**, Signal-to-noise ratios as a function of the imaging depth under different transmission strategies. **c**, Simulated acoustic fields of the wide-beam compounding, with enhanced acoustic field across the entire insonation area. **d**, Elevational, lateral and axial resolutions of the device using wide-beam compounding at different depths. **e**, Lateral and axial resolutions of the device using wide-beam compounding with different lateral distances from the central axis. Data in **d** and **e** are mean and s.d. from five tests (*n* = 5). **f**, Imaging inclusions with different contrasts to the matrix. On the basis of these B-mode images, the dynamic range (**g**) and contrast-to-noise ratio (**h**) of the device can be quantified.

The transmit beamforming strategy is critical for image quality. Therefore, we compared three distinct strategies: plane-wave, mono-focus and wide-beam compounding. Phantoms containing monofilament wires were used for this comparison (Supplementary Fig. 11, position 1). Among the three strategies, the wide-beam compounding implements a sequence of divergent acoustic waves with a series of transmission angles, and the generated images of each transmission are coherently combined to create a compounding image, which has the best quality with an expanded sonographic window^27^ (Fig. 2a,b and Supplementary Figs. 12–14). We also used a receive beamforming strategy to further improve the image quality (Supplementary Fig. 15 and Methods). The wide-beam compounding achieves a synthetic focusing effect and, therefore, a high acoustic intensity across the entire insonation area (Fig. 2c and Supplementary Fig. 13), which leads to the best signal-to-noise ratio and spatial resolutions (Fig. 2a, third column, Fig. 2b and Supplementary Fig. 12).

To quantify the device spatial resolutions using the wide-beam compounding strategy, we measured full widths at half maximum from the point spread function curves^28^ extracted from the images (Fig. 2a, third and fourth columns and the bottom row and Supplementary Fig. 11, positions 1 and 2). As the depth increases, the elevational resolution deteriorates (Fig. 2d) because the beam becomes more divergent in the elevational direction. Therefore, we integrated six small elements into a long element (Extended Data Fig. 1) to offer better acoustic beam convergence and elevational resolution. The lateral resolution deteriorates only slightly with depth (Fig. 2d) owing to the process of receive beamforming (Methods). The axial resolution remains almost constant with depth (Fig. 2d) because it depends only on the frequency and bandwidth of the transducer array. Similarly, at the same depth, the axial resolution remains consistent with different lateral distances from the central axis of the device, whereas the lateral resolution is the best at the centre, where there is a high overlap of acoustic beams after compounding (Fig. 2e and Methods).

Another critical metric for imaging is the location accuracies. The agreements between the imaging results and the ground truths (the red dots in Fig. 2a) in the axial and lateral directions are 96.01% and 95.90%, respectively, indicating excellent location accuracies (Methods).

Finally, we evaluated the dynamic range and contrast-to-noise ratio of the device using the wide-beam compounding strategy. Phantoms containing cylindrical inclusions with different acoustic impedances were used for the evaluation (Supplementary Fig. 11, position 3). A high acoustic impedance mismatch results in images with high contrast (Fig. 2f). We extracted the average grey values of the inclusion images and performed a linear regression^29^, and determined the dynamic range to be 63.2 dB (Fig. 2g, Supplementary Fig. 16 and Methods), which is well above the 60-dB threshold typically used in medical diagnosis^30^.

We selected two regions of interest, one inside and the other outside each inclusion area, to derive the contrast-to-noise ratios^31^, which range from 0.63 to 2.07 (Fig. 2h and Methods). A higher inclusion contrast leads to a higher contrast-to-noise ratio of the image. The inclusions with the lowest contrast (+3 dB or −3 dB) can be clearly visualized, demonstrating the outstanding sensitivity of this device^20^. The performance of the wearable imager is comparable with that of the commercial device (Supplementary Figs. 17 and 18, Extended Data Table 1 and Supplementary Discussion 5).

## Echocardiography from several views

**Extended Data Fig. 2:**
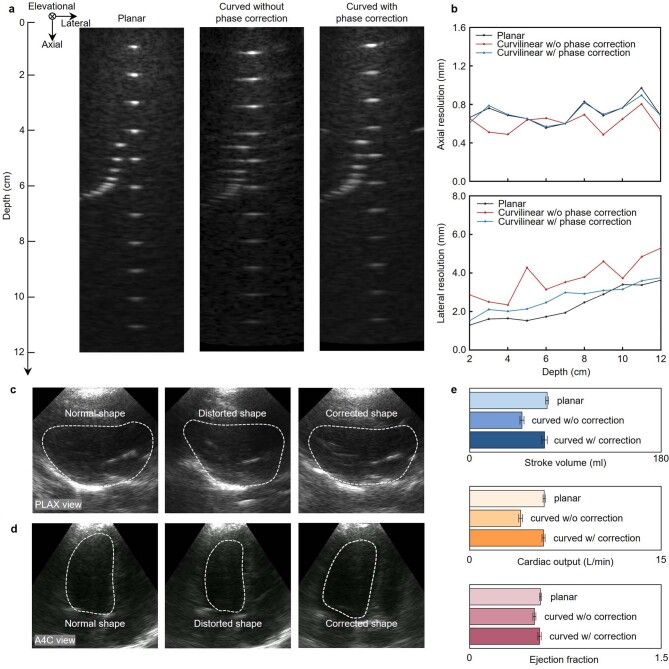
Characterization of the effects of phase correction on imaging quality. B-mode images of a line phantom obtained from different situations (**a**). Left, from a planar surface. Middle, from a curvilinear surface without phase correction. Right, from a curvilinear surface with phase correction. **b**, The axial and lateral resolutions at different depths under these three situations. No obvious difference in axial resolution was found because it is mainly dependent on the transducer frequency and bandwidth. The lateral resolution of the wearable imager was improved after phase correction. Images collected in (**c**), the parasternallong-axis (PLAX) view of the heart and (**d**), the apical four-chamber view when measured by a planar probe (left panel), a curved probe without phase correction (middle panel) and a curved probe with phase correction (right panel). The left ventricular boundaries are labelled by white dashed lines in the images. **e**, Comparison of measured cardiac indices showing the impact of phase correction. Each measurement is based on the mean of five consecutive cardiac cycles (n = 5). The standard deviations are indicated by the error bars.

Echocardiography is commonly used to examine the structural integrity and blood-delivery capabilities of the heart. Uniquely for soft devices, the contours of the human chest cause a non-planar distribution of the transducer elements, which leads to phase distortion and therefore image artefacts^32^. We used a three-dimensional scanner to collect the chest curvature to compensate for element position shifts within the wearable imager and thus correct phase distortion during transmit and receive beamforming (Supplementary Fig. 19, Extended Data Fig. 2 and Supplementary Discussion 6).

**Fig. 3:**
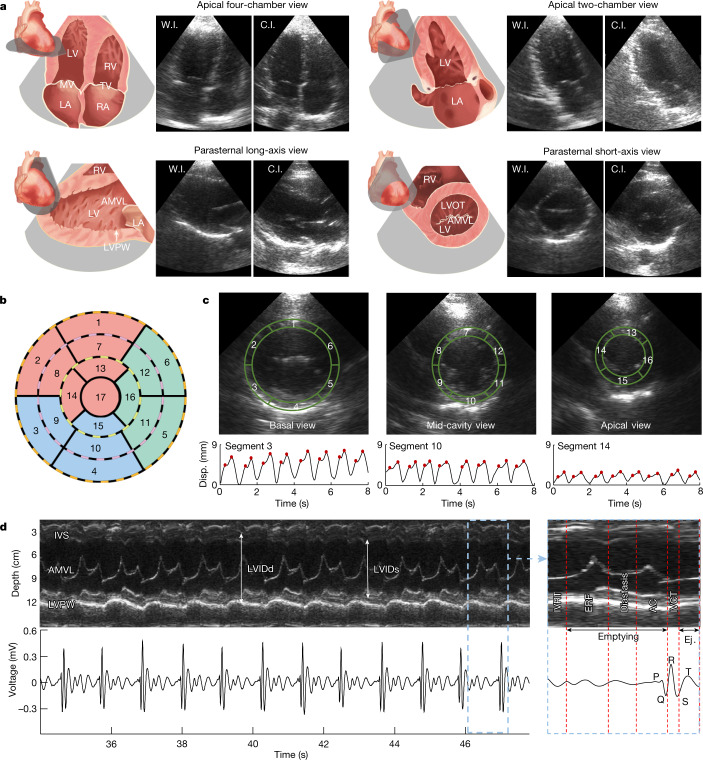
Echocardiography in several standard views. **a**, Schematics and B-mode images of cardiac anatomies from the wearable and commercial imagers. The wearable imager was placed in the parasternal position for imaging in the parasternal long-axis and short-axis views and relocated at the apical position for imaging in the apical four-chamber and two-chamber views. **b**, 17-segment model representation of the left ventricular wall. Each of the concentrically nested rings that make up the circular plot represents the parasternal short-axis view of the myocardial wall from a different level of the left ventricle. **c**, B-mode images of the left ventricle in basal, mid-cavity and apical views (top row) and corresponding typical displacement for segments 3, 10 and 14, respectively (bottom row). The physical regions of the left ventricular wall represented by each segment of the 17-segment model have been labelled on the corresponding short-axis views. The peaks are marked with red dots. **d**, M-mode images (upper left) extracted from parasternal long-axis view and corresponding electrocardiogram signals (lower left). A zoomed-in plot shows the different phases of a representative cardiac cycle (right). Primary events include diastole and opening of the mitral valve during the P-wave of the electrocardiogram, opening of the aortic valve and systole during the QRS complex and closure of the aortic valve during the T-wave. AC, atrial contraction; AMVL, anterior mitral valve leaflet; C.I., commercial imager; ERF, early rapid filling; Ej., ejection; IVCT, isovolumetric contraction time; IVRT, isovolumetric relaxation time; IVS, interventricular septum; LA, left atrium; LV, left ventricle; LVIDd, left ventricular internal diameter end diastole; LVIDs, left ventricular internal diameter end systole; LVOT, left ventricular outflow tract; LVPW, left ventricular posterior wall; MV, mitral valve; RA, right atrium; RV, right ventricle; TV, tricuspid valve; W.I., wearable imager.

**Extended Data Fig. 3:**
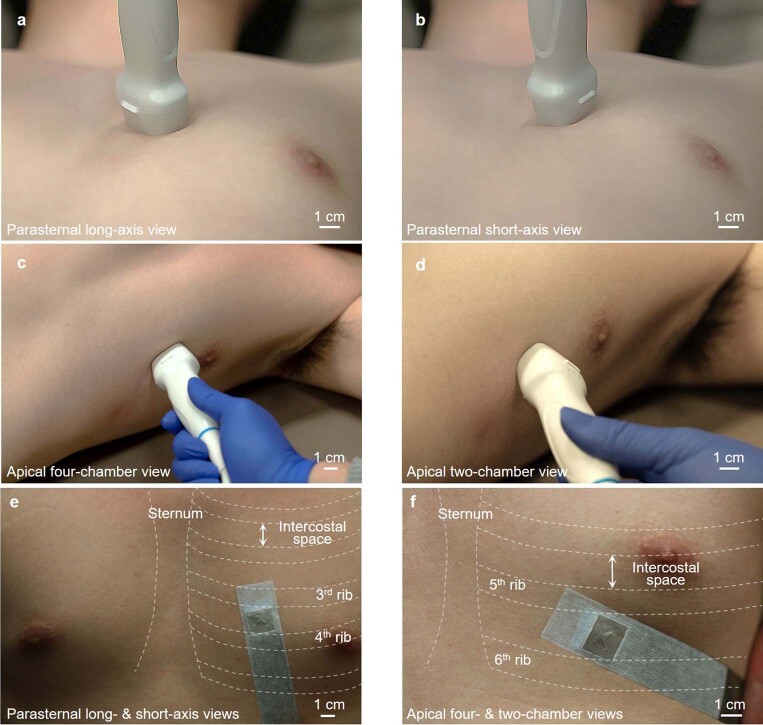
Optical images showing positions and orientations for ultrasound heart imaging. **a**, Parasternal long-axis view. **b**, Parasternal short-axis view. **c**, Apical four-chamber view. **d**, Apical two-chamber view. The orthogonal wearable cardiac imager combines parasternal long-axis and short-axis views (**e**) and apical four-chamber and apical two-chamber views without rotation (**f**). The wearable imager can capture two parasternal views from a single position or two apical views from another single position. The sternum and ribs are labelled to indicate intercostal spaces.

We compared the performance of the wearable device with a commercial device in four primary views of echocardiography, in which critical cardiac features can be identified (Extended Data Fig. 3). Figure 3a shows the schematics and corresponding B-mode images of these four views, including apical four-chamber view, apical two-chamber view, parasternal long-axis view and parasternal short-axis view. The difference between the results from the wearable and commercial devices is negligible. The parasternal short-axis view is particularly useful for evaluating the contractile function of the myocardium based on its motion in the radial direction and its relative thickening, as both are easily seen from this view. During contraction and relaxation, healthy myocardium undergoes strain and the wall thickness changes accordingly: thickening during contraction and thinning during relaxation. The strength of the left ventricle’s contractile function can be directly reflected on the ultrasound image through the magnitude of the myocardial strain. Abnormalities in the contractile function, such as akinesia, can be indicative of ischaemic heart disease and myocardial infarction^33^.

To better localize the specific segment of the left ventricular wall that is potentially pathological, the 17-segment model can be adopted as in standard clinical practice^33^ (Fig. 3b). We took the basal, mid-cavity and apical slices of the parasternal short-axis view from the left ventricular wall, and divided them into segments according to the model. Each segment is linked to a certain coronary artery, allowing ischaemia in the coronary arteries to be localized on the basis of akinesia in the corresponding myocardial segment^33^. We then recorded the displacement waveforms of the myocardium boundaries (Fig. 3c and Supplementary Discussion 6). The two peaks in each cardiac cycle in the displacement curves correspond to the two inflows into the left ventricle during diastole. The wall displacements, as measured in the basal, mid-cavity and apical views, become sequentially smaller owing to the decreasing radius of the myocardium along the conical shape of the left ventricle.

Motion-mode (M-mode) images track activities over time in a one-dimensional target region^34,35^. We extracted M-mode images from parasternal long-axis view B-mode images (Fig. 3d). Primary targets include the left ventricular chamber, the septum and the mitral/aortic valves. In M-mode, structural information, such as the myocardial thickness and the left ventricular diameter, can be tracked according to the distances between the boundaries of features. Valvular functions, for example, their opening and closing velocities, can be evaluated on the basis of the distance between the leaflet and septal wall (Supplementary Discussion 1). Moreover, we can correlate the mechanical activities in the M-mode images with the electrical activities in the electrocardiogram measured simultaneously during different phases in a cardiac cycle (Fig. 3d and Supplementary Discussions 1 and 6).

## Monitoring during motion

Stress echocardiography assesses cardiac responses to stress induced by exercise or pharmacologic agents, which may include new or worsened ischaemia presenting as wall-motion abnormalities, and is crucial in the diagnosis of coronary artery diseases^36^. Furthermore, subjects with heart failure may sometimes seem asymptomatic at rest, as the heart sacrifices its efficiency to maintain the same cardiac output^37,38^. Thus, by pushing the heart towards its limits during exercise, the lack of efficiency becomes apparent. However, in current procedures, ultrasound images are obtained only before and after exercise. With the cumbersome apparatus, it is impossible to acquire data during exercise, which may contain invaluable real-time insights when new abnormalities initiate^39^ (Supplementary Discussion 7). Also, because images are traditionally obtained after exercise, a quick recovery can mask any transient pathologic response during stress and lead to false-negative examinations^40^. In addition, the end point for terminating the exercise is subjective, which may result in suboptimal testing.

**Extended Data Fig. 4:**
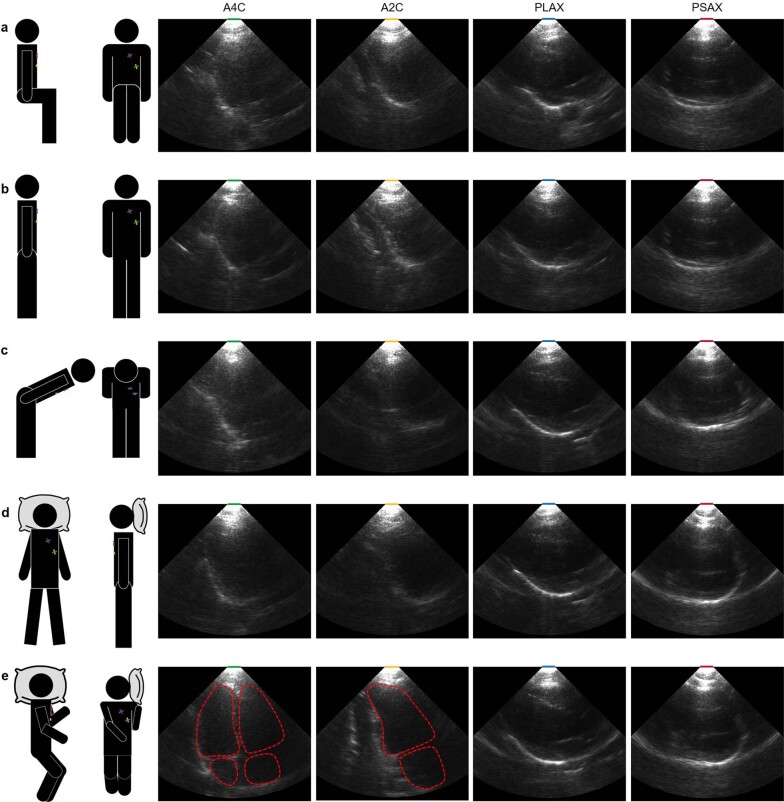
B-mode images collected from a subject with different postures. The four views collected when the subject is sitting (**a**), standing (**b**), bending over (**c**), lying flat (**d**) and lying on their side (**e**). The PLAX and PSAX views can keep their quality at different postures, whereas the quality of A4C and A2C views can only be achieved when lying on the side. A2C, apical two-chamber view; A4C, apical four-chamber view; PLAX, parasternal long-axis view; PSAX, parasternal short-axis view.

The wearable ultrasonic patch is ideal for overcoming these challenges. The device can be attached to the chest with minimal constraint to the movement of the subject, providing a continuous recording of cardiac activities before, during and after exercise with negligible motion artefacts (Extended Data Fig. 4). This not only captures the real-time responses during the test but also offers objective data to standardize the end point and enhances patient safety throughout the test (Supplementary Discussion 7). We used liquidus silicone as the couplant to achieve images of stable quality instead of water-based ultrasound gels that evaporate over time (Supplementary Figs. 20 and 21 and Supplementary Discussion 8). We observed no skin irritation or allergy after 24 h of continuous wear (Supplementary Fig. 22). The heart rate of the subject remained stable with a constant device temperature of about 32 °C after the device continuously worked for 1 h (Supplementary Fig. 23). In addition, one device was tested on different subjects (Supplementary Fig. 24). The reproducible results indicate the stable and reliable performance of the wearable imager.

**Fig. 4:**
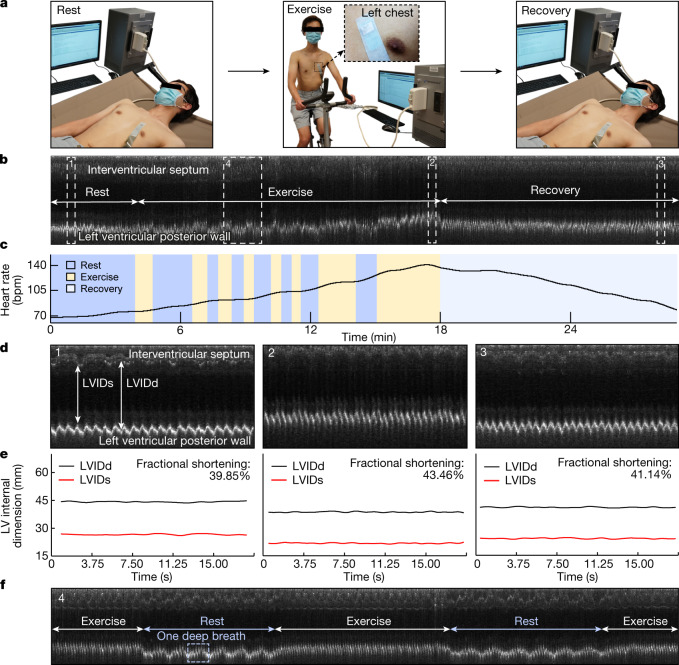
Monitoring during motion. **a**, Three stages of stress echocardiography. In the rest stage, the subject laid supine for around 4 min. In the exercise stage, the subject rode a stationary bike for about 15 min, with intervals for rest. In the recovery stage, the subject laid supine again for about 10 min. The wearable imager was attached to the chest of the subject throughout the entire test, even during the transitions between the stages. **b**, Continuous M-mode echocardiography extracted from the parasternal long-axis-view B-mode images of the entire process. Key features of the interventricular septum and left ventricular posterior wall are identified. The stages of rest, exercise (with intervals of rest) and recovery are labelled. **c**, Variations in the heart rate extracted from the M-mode echocardiography. **d**, Zoomed-in images of sections 1 (rest), 2 (exercise) and 3 (recovery) (dashed boxes) in **b**. **e**, Left ventricular internal diameter end diastole (LVIDd) and left ventricular internal diameter end systole (LVIDs) waveforms of the three different sections of the recording and corresponding average fractional shortenings. **f**, Zoomed-in images of section 4 (dashed box) during exercise with intervals of rest in **b**. In the first interval, the subject took a rhythmic deep breath six times, whereas during exercise, there seems to be no obvious signs of a deep breath, probably because the subject switched from diaphragmatic (rest) to thoracic (exercise) breathing, which is shallower and usually takes less time.

**Extended Data Fig. 5:**
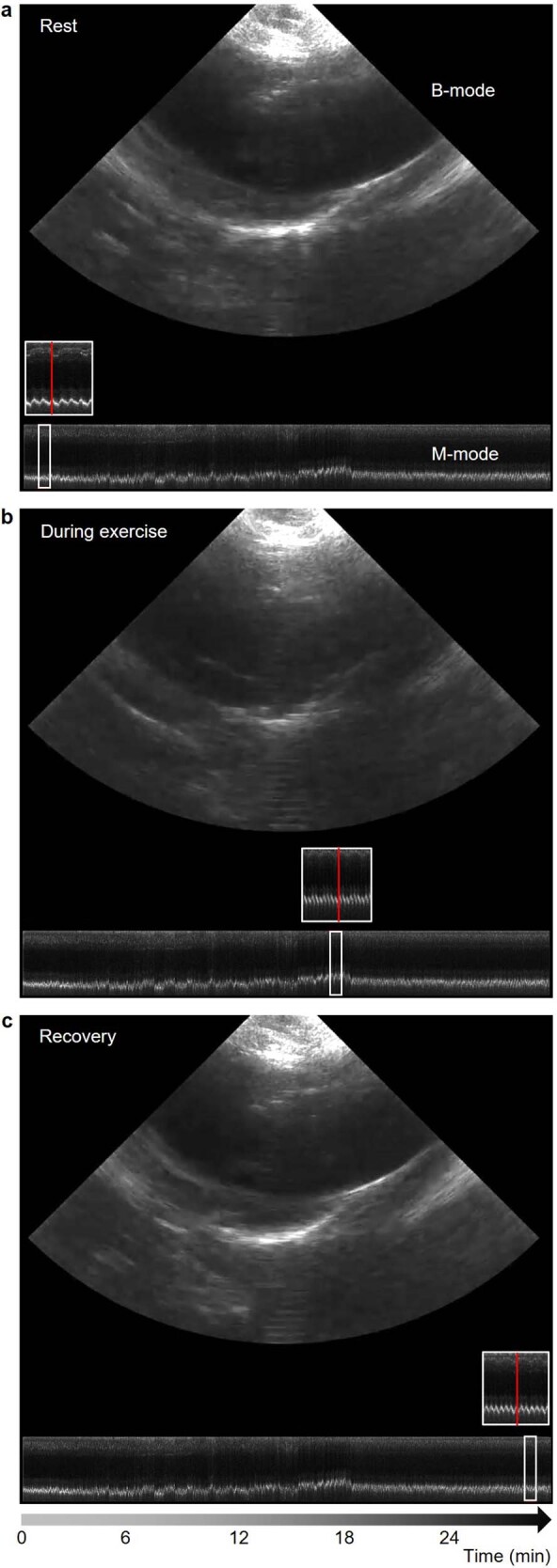
Continuous cardiac imaging during rest, exercise and recovery. Representative B-mode and M-mode images during rest (**a**), exercise (**b**) and recovery (**c**). The red line highlights the M-mode section corresponding to the current B-mode frame. More details can be seen in Supplementary Video 3.

We performed stress echocardiography to demonstrate the performance of the device during exercise (Supplementary Discussion 7). The device was attached to the subject for continuous recording along the parasternal long axis during the entire process, which consisted of three main stages (Fig. 4a). In the rest stage, the subject laid in the supine position. In the exercise stage, the subject exercised on a stationary bike with several intervals until a possible maximal heart rate was reached. In the recovery stage, the subject was placed in the supine position again. The device demonstrated uninterrupted tracking of the left ventricular activities, including the corresponding M-mode echocardiography and synchronized heart-rate waveform (Fig. 4b,c, Extended Data Fig. 5 and Supplementary Video 3). We examined a representative section of each testing stage and extracted the left ventricular internal diameter end systole (LVIDs) and left ventricular internal diameter end diastole (LVIDd) (Fig. 4d). The LVIDs and LVIDd of the subject remained stable during the rest stage (Fig. 4e). In the exercise stage, the interventricular septum and left ventricular posterior wall of the subject moved closer to the skin surface, with the latter moving more than the former, resulting in a decrease in LVIDs and LVIDd. In the recovery stage, the LVIDs and LVIDd slowly returned to normal. The variation in fractional shortening, a measure of the cardiac muscular contractility, reflects the changing demand for blood supply in different stages of stress echocardiography (Fig. 4e). Particularly, section 4 in Fig. 4b includes periods of exercise and intervals for rest, when patterns of a deep breath can also be seen from the left ventricular posterior wall motions (Fig. 4f).

## Automatic image processing

Cardiovascular diseases are often associated with changes in the pumping capabilities of the heart, which could be measured by stroke volume, cardiac output and ejection fraction. Non-invasive, continuous monitoring of these indices are valuable for the early detection and surveillance of cardiovascular conditions (Supplementary Discussion 9). Critical information embodied in these waveforms may help precisely determine potential risk factors and track the health state^41^ (Supplementary Discussion 10). On the other hand, processing of the unprecedented image data streams, if done manually, can be overwhelming for clinicians, which potentially introduces interobserver variability or even errors^42^.

**Fig. 5:**
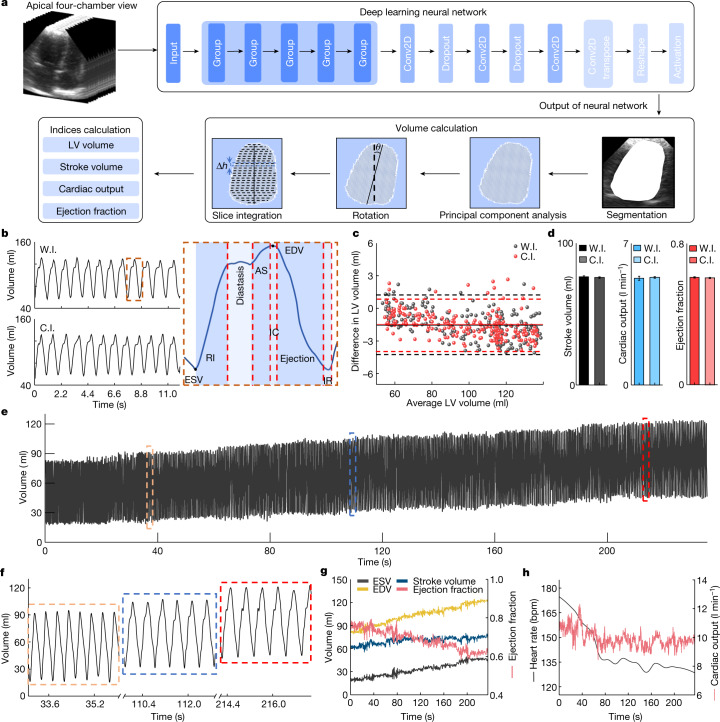
Automatic image processing by deep learning. **a**, Schematic workflow. Pre-processed images are used to train the FCN-32 model. The trained model accepts the unprocessed images and predicts the left ventricular (LV) volume, based on which stroke volume, cardiac output and ejection fraction are derived. **b**, Left ventricular volume waveform generated by the FCN-32 model from both the wearable imager (W.I.) and the commercial imager (C.I.) (left). Critical features are labelled in one detailed cardiac cycle (right). **c**, Bland–Altman analysis of the average of (*x* axis) and the difference between (*y* axis) the model-generated and manually labelled left ventricular volumes for the wearable (black) and commercial (red) imagers. Dashed lines indicate the 95% confidence interval and about 95% of the data points are within the interval for both imagers. Solid lines indicate mean differences. **d**, Comparing the stroke volume, cardiac output and ejection fraction extracted from results by the wearable and commercial imagers. Data are mean and s.d. from twelve cardiac cycles (*n* = 12). **e**, The model-generated left ventricular volume waveform in the recovery stage. **f**, Three representative sections of the recording from the initial, middle and end stages of **e**. End-systolic volume (ESV), end-diastolic volume (EDV), stroke volume and ejection fraction (**g**) and cardiac output and heart rate waveforms (**h**) derived from the left ventricular volume waveform. The end-systolic volume and end-diastolic volume gradually recover to the normal range in the end section. The stroke volume increases from about 60 ml to about 70 ml. The ejection fraction decreases from about 80% to about 60%. The cardiac output decreases from about 11 l min^−1^ to about 9 l min^−1^, indicating that the decreasing heart rate from about 175 bpm to about 130 bpm overshadowed the increasing stroke volume. AS, atrial systole; IC, isovolumetric contraction; IR, isovolumetric relaxation; RI, rapid inflow.

**Extended Data Fig. 6:**
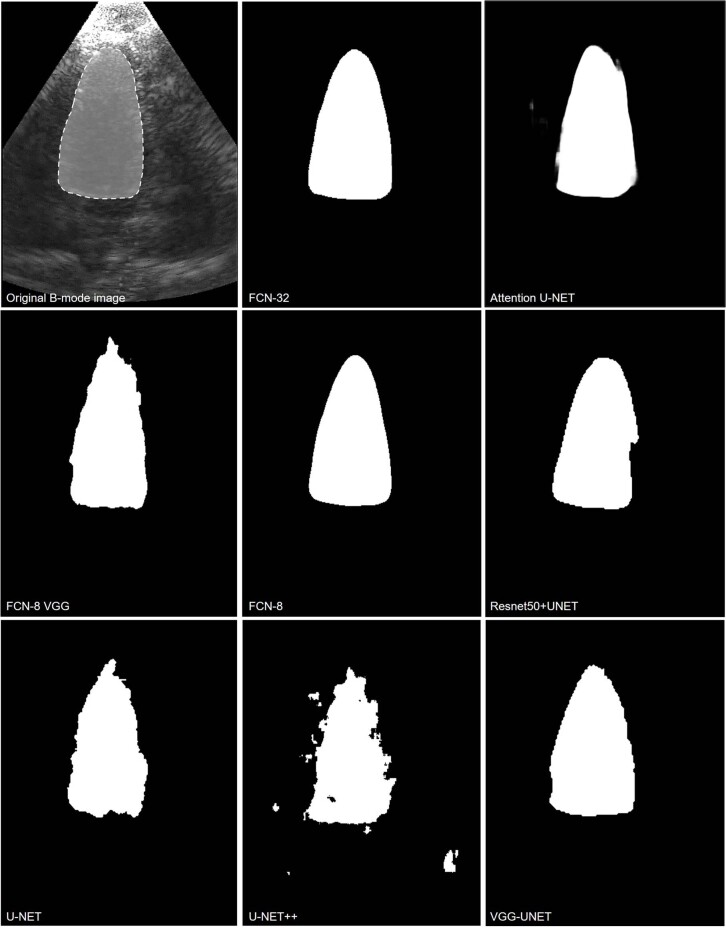
Segmentation results of the left ventricle with different deep learning models. By qualitatively evaluating the result, we found no ‘jitteriness’ in Supplementary Video 4. The segmented left ventricle contracts and relaxes as naturally as the B-mode video. The segmentation boundaries are smooth with the highest fidelity. Compared with the original B-mode image, the FCN-32 model has the best agreement among all models used in this study.

**Extended Data Fig. 7:**
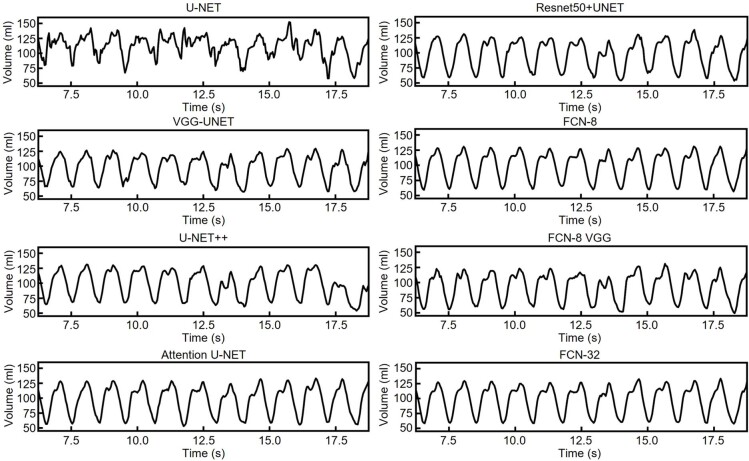
Waveforms of the left ventricular volume obtained with different deep learning models. Those waveforms are from segmenting the same B-mode video. Qualitatively, the waveform generated by the FCN-32 model gains the best stability and the least noise, and the waveform morphology is more constant from cycle to cycle. Quantitatively, the comparison results of those models is in Supplementary Fig. 26, which shows that the FCN-32 model has the highest mean intersection over union, showing the best performance in this study.

Automatic image processing can overcome the challenges. We applied a deep learning neural network to extract key information (for example, the left ventricular volume in apical four-chamber view) from the continuous stream of images (Fig. 5a, Supplementary Fig. 25 and Supplementary Discussion 11). We evaluated different types of deep learning models^43^ through the output images and waveforms of the left ventricular volume (Extended Data Figs. 6 and 7, Supplementary Table 3 and Supplementary Video 4). The FCN-32 model outperforms others based on qualitative and quantitative analyses (Supplementary Fig. 26, Supplementary Tables 4 and 5 and Supplementary Discussion 11). We also applied data augmentation to expand the dataset and improve the performance (Supplementary Fig. 27 and Supplementary Discussion 11).

**Extended Data Fig. 8:**
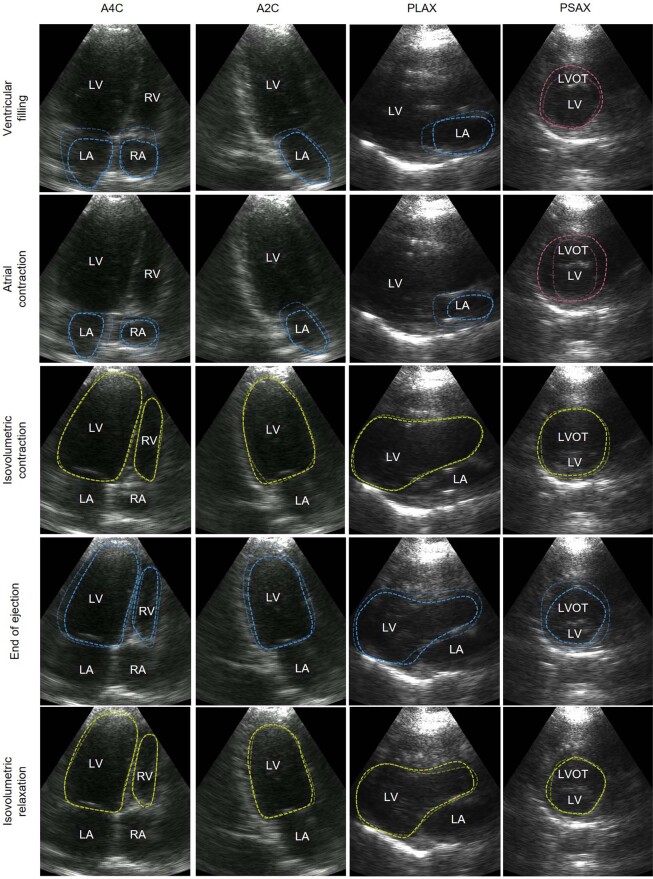
Different phases in a cardiac cycle obtained from B-mode imaging. The rows are B-mode images of A4C, A2C, PLAX and PSAX views in the same phase. The columns are B-mode images of the same view during ventricular filling, atrial contraction, isovolumetric contraction, end of ejection and isovolumetric relaxation. The dashed lines highlight the main features of the current phase. Bluish lines mean shrinking in the volume of the labelled chamber. Reddish lines mean expansion in the volume of the labelled chamber. Yellowish lines mean retention in the volume of the labelled chamber. A2C, apical two-chamber view; A4C, apical four-chamber view; LA, left atrium; LV, left ventricle; LVOT, left ventricular outflow tract; RA, right atrium; RV, right ventricle; PLAX, parasternal long-axis view; PSAX: parasternal short-axis view.

The output left ventricular volumes for the wearable and commercial imagers show similar waveform morphologies (Fig. 5b, left). From the waveforms, corresponding phases of a cardiac cycle can be identified (Fig. 5b, right and Extended Data Fig. 8). Bland–Altman analysis gives a quantitative comparison between the model-generated and manually labelled left ventricular volumes, indicating a stable and reliable performance of the FCN-32 model^44^ (Fig. 5c and Supplementary Discussion 11). The mean differences in the left ventricular volume are both approximately −1.5 ml, which is acceptable for standard medical diagnosis^45^. We then derived stroke volume, cardiac output and ejection fraction from the left ventricular volume waveforms. No marked difference is observed in the averages or standard deviations between the two devices (Fig. 5d). The results verified the comparable performance of the wearable imager to the commercial imager.

The left ventricular volume is constantly changing and generally follows a steady-state pattern at rest in healthy subjects. Therefore, stroke volume, cardiac output and ejection fraction also tend to remain constant. However, cardiac pathologies or ordinary daily activities such as exercise may dynamically change those indices. To validate the performance of the wearable imager under dynamic situations, we extracted the left ventricular volume from recordings in the recovery stage of stress echocardiography (Fig. 5e). The dimensions of the left ventricle cannot be accurately determined when the images are collected in the standing position, owing to anatomical limitations of the human body (Supplementary Fig. 28 and Supplementary Discussion 9). Owing to the deep breathing after exercise, the heart was sometimes blocked by the lungs in the image. We used an image-imputation algorithm to complement the blocked part (Supplementary Fig. 29 and Supplementary Discussion 11). The acquired waveform shows an increasing trend in the left ventricular volume. Figure 5f illustrates three representative sections of the recording taken from the beginning, middle and end of the recovery stage. In the initial section, the diastasis stage is barely noticeable because of the high heart rate. In the middle section, the diastasis stage becomes visible. In the end section, the heart rate decreases notably. The end-diastolic and end-systolic volumes are increasing, because the slowing heartbeat during recovery allows more time for blood to fill the left ventricle^46^ (Fig. 5g). The stroke volume gradually increases, indicating that the end-diastolic volume increases slightly faster than the end-systolic volume (Fig. 5g). The ejection fraction decreases as heart contraction decreases during the recovery (Fig. 5g). The cardiac output reduces, indicating a larger influence brought about by the decreasing heart rate than the increasing stroke volume (Supplementary Discussion 9).

## Discussion

Echocardiography is crucial in the diagnosis of cardiac diseases, but the current implementation in clinics is cumbersome and limits its application in continuous monitoring. Emerging technologies based on wearable rigid modules^25^ or flexible patches^47^ lack one or more of the ideal properties of wearable ultrasound technologies (Extended Data Table 2). In this work, we provided uninterrupted frame-by-frame acquisitions of cardiac images even when the subject was undertaking intensive exercise. In addition, the wearable imager with deep learning gave actionable information by automatically and continuously outputting curves of critical cardiac metrics, such as myocardial displacement, stroke volume, ejection fraction and cardiac output, which are highly desirable in critical care, cardiovascular disease management and sports science^48^. This capability is unprecedented in conventional clinical practice^9^ and the non-invasiveness can extend potential benefits to the outpatient and athletic populations.

The implications of this technology go far beyond imaging the heart, as it can be generalized to image other deep tissues, such as the inferior vena cava, abdominal aorta, spine and liver (Supplementary Fig. 30). For example, as demonstrated in an ultrasound-guided biopsy procedure on a cyst phantom (Supplementary Fig. 31), the two orthogonal imaging sections present the entire biopsy process simultaneously, freeing up one hand of the operator (Supplementary Video 5). The uniquely enabling capability of this technology forgoes the need for an operator to constantly hold the device.

Other future efforts could ensue by further improving spatial resolutions (Supplementary Fig. 32). A three-dimensional scanner can only provide the curvature of a static human chest. To accommodate the dynamic chest curvature, advanced imaging algorithms need to be developed to compensate for the phase distortion and thus improve spatial resolutions. In addition, the wearable imager is connected to the back-end system for data processing by means of a flexible cable (Supplementary Fig. 33) and future work needs to focus on system miniaturization and integration. Besides, the FCN-32 neural network can only be applied to subjects in the training dataset at present. Its generalizability could potentially be improved by expanding the training dataset or optimizing the network with few-shot-learning^49^ or reinforcement-learning^50^ strategies, which will allow the model to adapt to a larger population.

## Methods

### Materials

Gallium–indium eutectic liquid metal, toluene, ethyl alcohol, acetone and isopropyl alcohol were purchased from Sigma-Aldrich. SEBS (G1645) was obtained from Kraton. Silicone (Ecoflex 00-30) was bought from Smooth-On as the encapsulation material of the device. Silicone (Silbione) was obtained from Elkem Silicones as the ultrasound couplant. Aquasonic ultrasound transmission gel was bought from Parker Laboratories. 1-3 composite (PZT-5H) was purchased from Del Piezo Specialties. Silver epoxy (Von Roll 3022 E-Solder) was obtained from EIS. Anisotropic conductive film cable was purchased from Elform.

### Design and fabrication of the wearable imager

We designed the transducer array in an orthogonal geometry, similar to a Mills cross array (Supplementary Fig. 34), to achieve biplane standard views simultaneously. For the transducers, we chose the 1-3 composite for transmitting and receiving ultrasound waves because it possesses superior electromechanical coupling^18^. In addition, the acoustic impedance of 1-3 composites is close to that of the skin, maximizing the acoustic energy propagating into human tissues^19^. The backing layer dampens the ringing effect, broadens the bandwidth and thus improves the spatial resolution^18,51^.

We used an automatic alignment strategy to fabricate the orthogonal array. The existing method of bonding the backing layer to the 1-3 composite was to first dice many small pieces of backing layer and 1-3 composite, and then bond each pair together one by one. A template was needed to align the small pieces. This method was of very low efficiency. In this study, we bond a large piece of backing layer with a large piece of 1-3 composite and then dice them together into small pieces with designed configurations. The diced array is then automatically aligned on adhesive tape with high uniformity and perfect alignment.

Electrodes based on eutectic gallium–indium liquid metal are fabricated to achieve better stretchability and higher fabrication resolution than existing electrodes based on serpentine-shaped copper thin film. Eutectic gallium–indium alloys are typically patterned through approaches such as stencil lithography^52^, masked deposition^53^, inkjet printing^54^, microcontact printing^55^ or microfluidic channelling^56^. Although these approaches are reliable, they are either limited in patterning resolution or require sophisticated photolithography or printing hardware. The sophisticated hardware makes fabrication complicated and time-consuming, which presents a challenge in the development of compact, skin-conformal wearable electronics.

In this study, we exploited a new technology for patterning. We first screen-printed a thin layer of liquid metal on a substrate. A key consideration before screen printing was how to get the liquid metal to wet the substrate. To solve this problem, we dispersed big liquid metal particles into small microparticles using a tip sonicator (Supplementary Fig. 2). When microparticles contacted air, their outermost layer generated an oxide coating, which lowered the surface tension and prevented those microparticles from aggregating. In addition, we used 1.5 wt.% SEBS as a polymer matrix to disperse the liquid metal particles because SEBS could wet well on the liquid metal surface. We also used SEBS as the substrate. Therefore, the SEBS in the matrix and the substrate could merge and cure together after screen printing, allowing the liquid metal layer to adhere to the substrate efficiently and uniformly. Then we used laser ablation to selectively remove the liquid metal from the substrate to form patterned electrodes.

The large number of piezoelectric transducer elements in the array requires many such electrodes to address each element individually. We designed a four-layered top electrode and a common ground electrode. There are SEBS layers between different layers of liquid metal electrodes as insulation. To expose all electrode layers to connect to transducer elements, we used laser ablation to drill vertical interconnect accesses^21^. Furthermore, we created a stretchable shielding layer using liquid metal and grounded it through a vertical interconnect access, which effectively protected the device from external electromagnetic noises (Supplementary Fig. 8).

Before we attached the electrodes to the transducer array, we spin-coated toluene–ethanol solution (volume ratio 8:2) on the top of the multilayered electrode to soften the liquid-metal-based elastomer, also known as ‘solvent-welding’. The softened SEBS provided a sufficient contact surface, which could help form a relatively strong van der Waals force between the electrodes and the metal on the transducer surface. After bonding the electrodes to the transducer array, we left the device at room temperature to let the solvent evaporate. The final bonding strength of more than 200 kPa is stronger than many commercial adhesives^22^.

To encapsulate the device, we irrigated the device in a petri dish with uncured silicone elastomer (Ecoflex 00-30, Smooth-On) to fill the gap between the top and bottom electrodes and the kerf among the transducer elements. We then cured the silicone elastomer in an oven for 10 min at 80 °C. As the filling material, it suppresses spurious shear waves from adjacent elements, effectively isolating crosstalk between the elements^18,19^. With that being said, we think the main reason for the suppressed spurious shear waves is because of the epoxy in the 1-3 composite, which limits the lateral vibration of the piezoelectric materials. The Ecoflex as the filling material may have contributed but not played a chief role because the kerf is not too wide, only 100 to 200 µm. We lifted off the glass slide on the top electrode and directly covered the top electrode with a shielding layer. Then we lifted off the glass slide on the bottom electrode to release the entire device. Finally, screen-printing an approximately 50-μm layer of silicone adhesive on the device surface completed the entire fabrication.

### Characterization of the liquid metal electrode

Existing wearable ultrasound arrays can achieve excellent stretchability by serpentine-shaped metal thin films as electrodes^19,26^. The serpentine geometry, however, severely limits the filling ratio of functional components, precluding the development of systems that require a high integration density or a small pitch. In this study, we chose to use liquid metal as the electrode owing to its large intrinsic stretchability, which makes the high-density electrode possible. The patterned liquid metal electrode had a minimum width of about 30 μm with a groove of about 24 μm (Supplementary Fig. 3), an order of magnitude finer than other stretchable electrodes^18,26,57^. The liquid metal electrode is ideal for connecting arrays with a small pitch^58^.

This liquid metal electrode exhibited high conductivity, exceptional stretchability and negligible resistance change under tensile strain (Fig. 1b and Supplementary Fig. 4). The initial resistance at 0% strain was 1.74 Ω (corresponding to a conductivity of around 11,800 S m^−1^), comparable with reported studies^59,60^. The resistance gradually increased with strain until the electrode reached the approximately 750% failure strain (Fig. 1b and Supplementary Fig. 4). The relative resistance is a parameter widely used to characterize the change in the resistance of a conductor (that is, the liquid metal electrode in this case) under different strains relative to the initial resistance^58–60^. The relative resistance is unitless. When the strain was 0%, the initial resistance *R*_0_ was 1.74 Ω. When the electrode was under 750% strain, the electrode was broken and the resistance *R* at the breaking point was measured to be 44.87 Ω. Therefore, the relative resistance (*R*/*R*_0_) at the breaking point was 25.79.

To investigate the electrode fatigue, we subjected them to 100% cyclic tensile strain (Fig. 1c). The initial 500 cycles observed a gradual increase in the electrode resistance because the liquid metal, when stretched, could expose more surfaces. These new surfaces were oxidized after contacting with air, leading to the resistance increase (Supplementary Fig. 4). After the initial 500 cycles, the liquid metal electrode exhibited stable resistance because, after a period of cycling, there were not many new surfaces exposed.

This study is the first to use liquid metal-based electrodes to connect ultrasound transducer elements. The bonding strength between them directly decides the robustness and endurance of the device. This is especially critical for the wearable patch, which will be subjected to repeated deformations during use. Therefore, we characterized the bonding strength of the electrode to the transducer element using a lap shear test. The liquid metal electrode was first bonded with the transducer element. The other sides of the electrode and the element were both fixed with stiff supporting layers. The supporting layer serves to be clamped by the tensile grips of the testing machine. Samples will be damaged if they are clamped by the grips directly. Then a uniaxial stretching was applied to the sample at a strain rate of 0.5 s^−1^. The test was stopped when the electrode was delaminated from the transducer element. A SEBS film was bonded with a transducer element and we performed the lap shear test using the same method. The peak values of the curve were used to represent the lap shear strength (Fig. 1d). The bonding strength between the pure SEBS film and the transducer element was roughly 250 kPa, and that between the electrode and the transducer element was about 236 kPa, which were both stronger than many commercial adhesives (Supplementary Table 2). The results indicate the robust bonding between the electrode and the element, preventing the electrodes from delamination under various deformations. This robust bonding does not have any limitations on the ultrasound pressures that can be transduced.

### Characterization of the transducer elements

The electromechanical coupling coefficient of the transducer elements was calculated to be 0.67, on par with that of commercial probes (0.58–0.69)^61^. This superior performance was largely owing to the technique for bonding transducer elements and electrodes at room temperature in this study, which protected the piezoelectric material from heat-induced damage and depolarization. The phase angle was >60°, substantially larger than most earlier studies^18,62^, indicating that most of the dipoles in the element aligned well after bonding^63^. The large phase angle also demonstrated the exceptional electromechanical coupling performance of the device. Dielectric loss is critical for evaluating the bonding process because it represents the amount of energy consumed by the transducer element at the bonding interface^20^. The average dielectric loss of the array was 0.026, on par with that of the reported rigid ultrasound probes (0.02–0.04)^64–66^, indicating negligible energy consumed by this bonding approach (Supplementary Fig. 1b). The response echo was characterized in time and frequency domains (Supplementary Fig. 1c), from which the approximately 35 dB signal-to-noise ratio and roughly 55% bandwidth were derived. The crosstalk values between a pair of adjacent elements and a pair of second nearest neighbours have been characterized (Supplementary Fig. 1d). The average crosstalk was below the standard −30 dB in the field, indicating low mutual interference between elements.

### Characterization of the wearable imager

We characterized the wearable imager using a commercial multipurpose phantom with many reflectors of different forms, layouts and acoustic impedances at various locations (CIRS ATS 539, CIRS Inc.) (Supplementary Fig. 11). The collected data are presented in Extended Data Table 1. For most of the tests, the device was first attached to the phantom surface and rotated to ensure the best imaging plane. Raw image data were saved to guarantee minimum information loss caused by the double-to-int8 conversion. Then the raw image data were processed using the ‘scanConversion’ function provided in the k-Wave toolbox to restore the sector-shaped imaging window (restored data). We applied five times upsampling in both vertical and lateral directions. The upsampled data were finally converted to the dB unit using:

1 $${I}_{{\rm{new}}}=20\times {\log }_{10}({I}_{{\rm{old}}})$$

The penetration depth was tested with a group of lines of higher acoustic impedance than the surrounding background distributed at different depths in the phantom. The penetration depth is defined as the depth of the deepest line that is differentiable from the background (6 dB higher in pixel value). Because the deepest line available in this study was at a depth of 16 cm and was still recognizable from the background, the penetration depth was determined as >16 cm.

The accuracy is defined as the precision of the measured distance. The accuracy was tested with the vertical and lateral groups of line phantoms. The physical distance between the two nearest pixels in the vertical and lateral directions was calculated as:

2 $$\Delta y=\frac{{\rm{imaging}}\,{\rm{d}}{\rm{e}}{\rm{p}}{\rm{t}}{\rm{h}}}{{N}_{{\rm{pixel}},{\rm{v}}{\rm{e}}{\rm{r}}{\rm{t}}{\rm{i}}{\rm{c}}{\rm{a}}{\rm{l}}}-1}$$

3 $$\Delta x=\frac{{\rm{imaging}}\,{\rm{w}}{\rm{i}}{\rm{d}}{\rm{t}}{\rm{h}}}{{N}_{{\rm{pixel}},{\rm{l}}{\rm{a}}{\rm{t}}{\rm{e}}{\rm{r}}{\rm{a}}{\rm{l}}}-1}$$

We acquired the measured distance between two lines (shown as two bright spots in the image) by counting the number of pixels between the two spots and multiplying them by Δ*y* or Δ*x*, depending on the measurement direction. The measured distances at different depths were compared with the ground truth described in the data sheet. Then the accuracy can be calculated by:

4 $${\rm{Accuracy}}=\,1-\left|\frac{{\rm{computed}}\,{\rm{d}}{\rm{i}}{\rm{s}}{\rm{t}}{\rm{a}}{\rm{n}}{\rm{c}}{\rm{e}}}{{\rm{ground}}\,{\rm{t}}{\rm{r}}{\rm{u}}{\rm{t}}{\rm{h}}}-1\right|$$

The lateral accuracy was presented as the mean accuracy of the four neighbouring pairs of lateral lines at a depth of 50 mm in the phantom.

The spatial resolutions were tested using the lateral and vertical groups of wires. For the resolutions at different depths, the full width at half maximum of the point spread function in the vertical or lateral directions for each wire was calculated. The vertical and lateral resolutions could then be derived by multiplying the number of pixels within the full width at half maximum by Δ*y* or Δ*x*, depending on the measurement direction. The elevational resolutions were tested by rotating the imager to form a 45° angle between the imager aperture and the lines. Then the bright spot in the B-mode images would reveal scatters out of the imaging plane. The same process as calculating the lateral resolutions was applied to obtain the elevational resolutions. The spatial resolutions at different imaging areas were also characterized with the lateral group of wires. Nine wires were located at ±4 cm, ±3 cm, ±2 cm, ±1 cm and 0 cm from the centre. The lateral and axial resolutions of the B-mode images from those wires were calculated with the same method.

Note that the lateral resolution worsens with the depth, mainly because of the receive beamforming (Supplementary Fig. 15). There are two beamformed signals, A and B. The lateral resolution of the A point (*x*_1_) is obviously better than that of the B point (*x*_2_). The fact that lateral resolution becomes worse with depth is inevitable in all ultrasound imaging, as long as receive beamforming is used.

As for different transmit beamforming methods, the wide-beam compounding is the best because it can achieve a synthetic focusing effect in the entire insonation area. The better the focusing effect, the higher the lateral resolution, which is why the lateral resolution of the wide-beam compounding is better than the other two transmit methods at the same depth. Furthermore, the multiple-angle scan used in the wide-beam compounding can enhance the resolution at high-angle areas. The multiple-angle scan combines transmissions at different angles to achieve a global high signal-to-noise ratio, resulting in improved resolutions.

The elevational resolution can only be characterized when the imaging target is directly beneath the transducer. For those targets that are far away from the centre, they are difficult to be imaged, which makes their elevational resolutions challenging to calculate. When characterizing the elevational resolution, the device should rotate 45°. In this case, most of the reflected ultrasound waves from those wires cannot return to the device owing to the large incidence angles. Therefore, those wires cannot be captured in the B-mode images. One potential solution is to decrease the rotating angle of the device, which may help capture more wires distributed laterally in the B-mode image. However, a small rotating angle will cause the elevational image to merge with the lateral image, which increases the error of calculating the elevational resolution. Considering those reasons, we only characterized the elevational resolution of the imaging targets directly beneath the transducer array.

The contrast resolution, the minimum contrast that can be differentiated by the imaging system, was tested with greyscale objects. The collected B-mode images are shown in Fig. 2. Because the targets with +3 and −3 dB, the lowest contrast available in this study, could still be recognized in the images, the contrast resolution of the wearable imager is determined as <3 dB.

The dynamic range in an ultrasound system refers to the contrast range that can be displayed on the monitor. The contrast between an object and the background is indicated by the average grey value of all pixels in the object in the display. The grey value is linearly proportional to the contrast. The larger the contrast, the larger the grey value. Because the display window was using the data type ‘uint8’ to differentiate the greyscale, the dynamic range was defined as the contrast range with a grey value ranging from 0 to 255.

The object with −15 dB contrast has the lowest average grey value, whereas the object with +15 dB contrast has the highest (Supplementary Fig. 16). In our case, there are six objects with different contrasts to the background in the phantom. The highest grey value obtained from the object of +15 dB contrast was 159.8, whereas the lowest grey value from the object of −15 dB contrast was 38.7. We used a linear fit to extrapolate the contrasts when the corresponding average grey values were equal to 255 and 0, which corresponded to contrasts of 39.2 dB and −24.0 dB, respectively. Then the dynamic range was determined as:

5 $${\rm{Dynamic}}\,{\rm{r}}{\rm{a}}{\rm{n}}{\rm{g}}{\rm{e}}=39.2-\left(-24.0\right)=63.2\,{\rm{dB}}$$

The dead zone is defined as the depth of the first line phantom that is not overwhelmed by the initial pulses. The dead zone was tested by imaging a specific set of wire phantoms with different depths right beneath the device (Supplementary Fig. 11, position 4) directly and measuring the line phantoms that were visible in the B-mode image.

The bandwidth of the imager is defined as the ratio between the full width at half maximum in the frequency spectrum and the centre frequency. It was measured by a pulse-echo test. A piece of glass was placed 4 cm away from the device and the reflection waveform was collected with a single transducer. The collected reflection waveform was converted to the frequency spectrum by a fast Fourier transform. The full width at half maximum was read from the frequency spectrum. We obtained the bandwidth using:

6 $${\rm{Bandwidth}}=\frac{{\rm{full}}\,{\rm{width}}\,{\rm{at}}\,{\rm{half}}\,{\rm{maximum}}}{{\rm{centre}}\,{\rm{frequency}}}$$

Contrast sensitivity represents the capability of the device to differentiate objects with different brightness contrasts^20^. The contrast sensitivity was tested with the greyscale objects. The contrast sensitivity is defined as the contrast-to-noise ratio (CNR) of the objects having certain contrasts to the background in the B-mode image:

7 $${\rm{CNR}}=\frac{\left|{\mu }_{{\rm{in}}}-{\mu }_{{\rm{out}}}\right|}{\sqrt{{\sigma }_{{\rm{in}}}^{2}+{\sigma }_{{\rm{out}}}^{2}}}$$

in which *μ*_in_ and *σ*_in_ are the mean and the standard deviation of pixel intensity within the object, and *μ*_out_ and *σ*_out_ are the mean and the standard deviation of pixel intensity of the background.

The insertion loss is defined as the energy loss during the transmission and receiving. It was tested in water with a quartz crystal, a function generator with an output impedance of 50 Ω and an oscilloscope (Rigol DS1104). First, the transducer received an excitation in the form of a tone burst of a 3-MHz sine wave from the function generator. Then the same transducer received the echo from the quartz crystal. Given the 1.9-dB energy loss of the transmission into the quartz crystal and the 2.2 × 10^−4^ dB (mm MHz)^−1^ attenuation of water, the insertion loss could be calculated as:

8 $${\rm{Insertion}}\,{\rm{l}}{\rm{o}}{\rm{s}}{\rm{s}}=\left|20\times {\log }_{10}\left(\frac{{V}_{{\rm{r}}}}{{V}_{{\rm{t}}}}\right)+1.9+2.2\times {10}^{-4}\times 2d\times {f}_{{\rm{r}}}^{2}\right|$$

### Simulation of the acoustic field

The simulation computes the root mean square of the acoustic pressure at each point in the defined simulation field. The root mean square is defined in the equation below and gives an average acoustic pressure over a certain time duration, which is pre-defined in a packaged function of the software. In the equation, *x*_*i*_ is the simulated acoustic pressure at the *i*th time step.

9 $${x}_{{\rm{RMS}}}=\sqrt{\frac{1}{n}({x}_{1}^{2}+{x}_{2}^{2}+\cdots +{x}_{n}^{2})}$$

Figure 2c is the simulated root mean square of the transmitted acoustic pressure field by the orthogonal transducers. The simulation was done using the MATLAB UltraSound Toolbox^67^. Each one-dimensional phased array in the orthogonal transducers gives a sector-shaped acoustic pressure field. The simulation merges two such sector-shaped acoustic pressure fields. The imaging procedure was done with the same parameters as the simulations.

In the simulation, we defined the transducer parameters first: the centre frequency of the transducers as 3 MHz, the width of the transducers as 0.3 mm, the length of the transducers as 2.3 mm, the pitch of the array as 0.4 mm, the number of elements as 32 and the bandwidth of the transducers as 55%. Then we defined wide-beam compounding (Supplementary Fig. 13) as the transmission method: 97 transmission angles, from −37.5° to +37.5°, with a step size of 0.78°. Then the acoustic pressure field was the overall effect of the 97 transmissions. Finally, we defined the computation area: −8 mm to +8 mm in the lateral direction, −6 mm to +6 mm in the elevational direction and 0 mm to 140 mm in the axial direction.

## Online content

Any methods, additional references, Nature Portfolio reporting summaries, source data, extended data, supplementary information, acknowledgements, peer review information; details of author contributions and competing interests; and statements of data and code availability are available at 10.1038/s41586-022-05498-z.

### Supplementary information

Supplementary Information This file contains Supplementary Discussions 1–11, Supplementary Figures 1–34, Supplementary Tables 1–5 and Supplementary References.

Supplementary Video 1 Cardiac long and short axis views imaged by an orthogonal array.

Supplementary Video 2 Cardiac apical four–and two–chamber views imaged by an orthogonal array.

Supplementary Video 3 Continuous cardiac imaging during rest, exercise, and recovery.

Supplementary Video 4 Left ventricle segmentation results by FCN-32.

Supplementary Video 5 Imaging guided biopsy on a phantom by an orthogonal array.

## Figures and Tables

**Extended Data Table 1:**
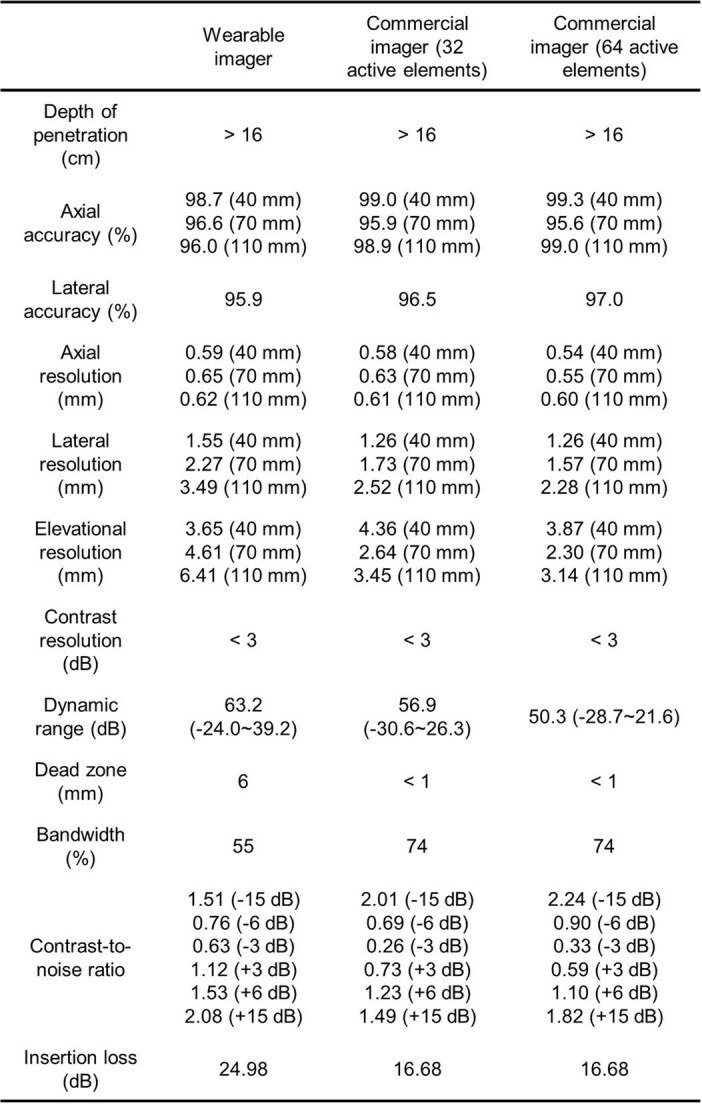
Full comparison of the imaging metrics between the wearable imager and a commercial ultrasound imager (Model P4-2v)

The overall performance of the wearable imager is comparable with that of the commercial one. Because the wearable orthogonal array has 32 elements in each direction, we also provided measurements of the commercial imager with only 32 elements activated.

**Extended Data Table 2:**
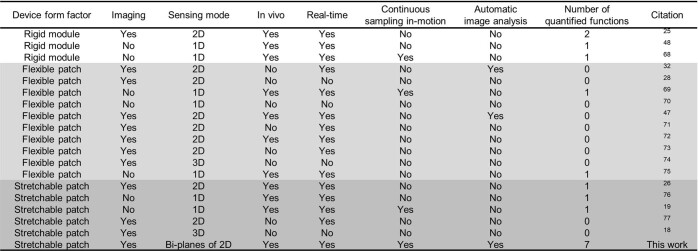
Summary of wearable ultrasonic devices for continuous monitoring of deep tissues

‘Number of quantified functions’ means the amount of physiological signals collected from images. For example, the number of quantified functions in this work is seven because we can extract myocardium displacement, left ventricular internal diameter, fractional shortening, stroke volume, ejection fraction, cardiac output and heart rate from those B-mode images.

## Data Availability

All data are available in the manuscript or Supplementary Information.

## Code availability

The code that produced the findings of this study is available at https://github.com/UCSD-XuGroup/Wearable-Cardiac-Ultrasound-Imager.
