# Supplementary material for: A wearable cardiac ultrasound imager
Source: Nature. 2023 Jan 25;613(7945):667–75. doi: 10.1038/s41586-022-05498-z (PMC9876798; doi:10.1038/s41586-022-05498-z)
Supplement: Supplementary file 1 — This file contains Supplementary Discussions 1–11, Supplementary Figures 1–34, Supplementary Tables 1–5 and Supplementary References. [file 41586_2022_5498_MOESM1_ESM.pdf]

nature portfolio

https://doi.org/10.1038/s41586-022-05498-z

# Supplementary information

# A wearable cardiac ultrasound imager

In the format provided by the authors and unedited

**Supplementary Information for**

# A wearable cardiac ultrasound imager

Hongjie Hu[1]*, Hao Huang[1]*, Mohan Li[2]*, Xiaoxiang Gao[1]*, Lu Yin[1], Ruixiang Qi[3], Ray S. Wu[1], Xiangjun Chen[4], Yuxiang Ma[1,5], Keren Shi[4,6], Chenghai Li[7], Timothy M. Maus[8], Brady Huang[9], Chengchangfeng Lu[2], Muyang Lin[1], Sai Zhou[4], Zhiyuan Lou[1], Yue Gu[4,10], Yimu Chen[1], Yusheng Lei[1,11], Xinyu Wang[1], Ruotao Wang[1], Wentong Yue[1], Xinyi Yang[4], Yizhou Bian[1], Jing Mu[4], Geonho Park[1], Shu Xiang[12], Shengqiang Cai[4,7], Paul W. Corey[13], Joseph Wang[1,4], Sheng Xu[1,2,4,9,14#]

[1]Department of Nanoengineering, University of California San Diego, La Jolla, CA 92093, USA.
[2]Department of Electrical and Computer Engineering, University of California San Diego, La Jolla, CA 92093, USA.
[3]Department of Computer Science and Engineering, University of California San Diego, La Jolla, CA 92093, USA.
[4]Materials Science and Engineering Program, University of California San Diego, La Jolla, CA 92093, USA.
[5]Department of Mechanical Engineering, Massachusetts Institute of Technology, Cambridge, MA 02139, USA.
[6]Materials science and engineering program, University of California Riverside, Riverside, CA 92521, USA.
[7]Department of Mechanical and Aerospace Engineering, University of California San Diego, La Jolla, CA 92093, USA.
[8]Department of Anesthesiology, UC San Diego Health-Sulpizio Cardiovascular Center, La Jolla, CA 92037, USA.
[9]Department of Radiology, School of Medicine, University of California San Diego, La Jolla, CA 92103, USA.
[10]Department of Neurosurgery, Yale University, New Haven, CT 06520, USA.
[11]Department of Chemical Engineering, Stanford University, Stanford, CA 94305, USA.
[12]Softsonics, Inc., San Diego, CA 92122, USA.
[13]Department of Anesthesiology, Sharp Memorial Hospital, San Diego 92123, CA, USA.
[14]Department of Bioengineering, University of California San Diego, La Jolla 92093, CA, USA.
*These authors contributed equally to this work.
[#]Email: shengxu@ucsd.edu

Content

**Supplementary Discussion 1: Significance of the wearable imager** 6
1.1 End-systolic volume (ESV), end-diastolic volume (EDV), heart rate, stroke volume, cardiac output, and ejection fraction 6
1.2 Cardiac functions and common pathologies 6
1.3 B-mode and M-mode images 9
1.4 Unique innovation of the wearable imager 9
1.5 Bandwidth improvement of the wearable imager 12
**Supplementary Discussion 2: Why wearable and why ultrasound?** 13
2.1 Why wearable? 13
2.2 General requirements of imaging the heart 15
2.3 Magnetic resonance imaging 16
2.4 X-ray computed tomography 16
2.5 Emission tomography 17
2.6 Optical coherence tomography 17
2.7 Why ultrasound? 17
2.8 Comparability of the wearable ultrasound to clinical ultrasound 18
**Supplementary Discussion 3: Configuration of the array** 20
3.1 The configuration of the array in this study 20
3.2 The Mills cross array 21
3.3 The 1.5D/1.75D array 21
**Supplementary Discussion 4: Possible sources of electromagnetic interference** 22
**Supplementary Discussion 5: Measurement variations of imaging metrics** 22
**Supplementary Discussion 6: Imaging procedures** 23
6.1 Imaging from different views 24
6.2 Phase correction on nonplanar surfaces 25
6.3 Validation for long-term use 28
6.4 Simultaneous measurements of M-mode images and electrocardiogram 28
**Supplementary Discussion 7: Stress echocardiography** 29

7.1 Significance 29

7.2 Limitations of existing procedures 29

7.3 Echocardiography by the wearable imager 30

**Supplementary Discussion 8: Acoustic coupling of wearable imager** 31

**Supplementary Discussion 9: Continuous cardiac performance monitoring** 32

9.1 Monitoring ejection fraction, cardiac output, and stroke volume simultaneously 32

9.2 Monitoring the left ventricular volume by 3D imaging 34

9.3 Monitoring the left ventricular volume by 2D imaging 35

9.4 Monitoring the left ventricular volume by model estimations 35

9.5 Anatomical considerations of imaging posture 41

**Supplementary Discussion 10: Detailed left ventricle working processes** 43

**Supplementary Discussion 11: Neural network for continuous ultrasound imaging** 44

11.1 Developing the deep learning model 44

11.2 Detailed analysis of the neural network 45

**Supplementary Fig. 1 | Characterization of the transducer array.** 50

**Supplementary Fig. 2 | Fabrication processes of the wearable imager.** 52

**Supplementary Fig. 3 | Images showing the fabrication resolution of the liquid metal composite electrodes.** 54

**Supplementary Fig. 4 | Mechanical testing of the liquid metal composite electrodes.** 55

**Supplementary Fig. 5 | Results of lap shear strength tests.** 56

**Supplementary Fig. 6 | Optical images of the multilayered liquid metal composite electrodes.** 57

**Supplementary Fig. 7 | The thickness of the SEBS substrate and the printed liquid metal composite.** 58

**Supplementary Fig. 8 | Characterization of noise levels after applying different strategies sequentially.** 59

**Supplementary Fig. 9 | Stress-strain curve of the entire device.** 60

**Supplementary Fig. 10 | Biaxial mechanical testing of the entire device.** 61

**Supplementary Fig. 11 | The structure of the phantom for device characterizations.** 62

**Supplementary Fig. 12 | Characterization of resolutions and acoustic fields with different transmission methods and angles.** 63

**Supplementary Fig. 13 | The mechanism of wide-beam compounding B-mode imaging.** 65

**Supplementary Fig. 14 | Signal-to-noise ratio as a function of step size and number of steering angles of the wide-beam compounding imaging.** 66

**Supplementary Fig. 15 | The flow chart of receive beamforming.** 67

**Supplementary Fig. 16 | Gray scale B-mode images of phantoms and selected windows for calculating the dynamic range.** 68

**Supplementary Fig. 17 | Detailed comparison of the imaging metrics between the wearable and the commercial imagers.** 69

**Supplementary Fig. 18 | Schematic experimental setups of resolution tests.** 70

**Supplementary Fig. 19 | Processes of evaluating the surface curvature for phase correction.** 72

**Supplementary Fig. 20 | B-mode images collected with different couplants.** 74

**Supplementary Fig. 21 | Quantitatively evaluation of different coupling conditions.** 75

**Supplementary Fig. 22 | Optical images of attaching the wearable imager to the chest for long-term.** 76

**Supplementary Fig. 23 | Continuous surface temperature and heart rate monitoring for 1 hour.** 77

**Supplementary Fig. 24 | Images of the parasternal long axis view from 10 subjects using a recycled device.** 78

**Supplementary Fig. 25 | The structure of the FCN-32 neural network.** 79

**Supplementary Fig. 26 | The comparison of the intersection over union among different models used in this study.** 81

**Supplementary Fig. 27 | The types and results of data augmentation.** 83

**Supplementary Fig. 28 | Imaging from apical four chamber view with different positions.** 84

**Supplementary Fig. 29 | Validation of the image imputation algorithm.** 86

**Supplementary Fig. 30 | B-mode images of the abdominal area and liver from the wearable and the commercial imagers.** 87

**Supplementary Fig. 31 | B-mode images of biopsy tests on a commercial phantom (CIRS 052).** 88

**Supplementary Fig. 32 | B-mode images of cardiac anatomies tested by an experienced cardiac sonographer.** 89

**Supplementary Fig. 33 | Photograph and schematics of the imaging system.** 90

**Supplementary Fig. 34 | Configuration of a Mills cross array.** 92

**Supplementary Table 1 | Summary of existing imaging methods for the heart.** 93

**Supplementary Table 2 | Comparison between the bonding strength of the liquid metal electrode, pure SEBS, and commercial adhesives.** 94

**Supplementary Table 3 | Model parameters and code availability.** 95

**Supplementary Table 4 | Sample sizes for all models.** 96

**Supplementary Table 5 | Mean Intersection over Union among different models.** 97

**Supplementary Video 1. Cardiac long and short axis views imaged by an orthogonal array.** 98

**Supplementary Video 2. Cardiac apical four- and two- chamber views imaged by an orthogonal array.** 98

**Supplementary Video 3. Continuous cardiac imaging during rest, exercise, and recovery.** 98

**Supplementary Video 4. Left ventricle segmentation results by FCN-32.** 98

**Supplementary Video 5. Imaging guided biopsy on a phantom by an orthogonal array.** 98

**References** 99

## Supplementary Discussion 1: Significance of the wearable imager

1.1 End-systolic volume (ESV), end-diastolic volume (EDV), heart rate, stroke volume, cardiac output, and ejection fraction

Normal cardiac function is essential for maintaining systemic tissue perfusion throughout the body[1]. Cardiovascular diseases, especially in the elderly, impose a huge burden in terms of mortality, morbidity, disability, and healthcare costs[2]. More than a million patients are admitted annually to U.S. hospitals with acute heart failure alone, together with a high median percentage of intensive care unit admission of 10%[68] and a high in-hospital mortality rate of around 4% to 7%[69]. Also, cardiovascular failure is one of the leading causes of death in intensive care units[70] and nearly one-quarter of all deaths in intensive care units are attributed to it.

Accurate assessment of subtle changes in cardiac functions is essential for health management and disease prevention for healthy people, as well as diagnosis of pathogenesis and interventions for patients. The signals we can use to evaluate the cardiac functions include the ESV, EDV, heart rate, as well as their derivative signals such as stroke volume, cardiac output, and ejection fraction. ESV and EDV can be obtained by processing the apical four-chamber view B-mode images using a deep learning model. The heart rate can be observed based on the period of contraction in M-mode images. Based on these values, those derivative signals can be calculated by:

$$Stroke\ volume = EDV - ESV \quad (10)$$

$$Cardiac\ output = Stroke\ volume * Heart\ rate \quad (11)$$

$$Ejection\ fraction = \frac{Stroke\ volume}{EDV} \quad (12)$$

Stroke volume indicates the absolute blood volume the left ventricle can pump out in a single stroke. The cardiac output indicates the absolute blood volume the left ventricle can pump out every minute. Ejection fraction indicates the relative fraction of the blood in the left ventricle that the heart can pump out in a single stroke. Altogether, these indices provide insight into the capability of the heart to deliver blood to tissues throughout the body.

1.2 Cardiac functions and common pathologies

Cells in the human body all require a steady supply of oxygen and nutrient for their metabolism. The cellular metabolic rates are not static, but rather are subject to constant fluctuations. Thus, the heart must not only be able to produce a cardiac output meeting the metabolic demands of the body at a given time but also do so efficiently, such that enough headroom is maintained to accommodate any heightened metabolic rates that can occur due to

circumstances such as strenuous exercise. The existence of a nonzero end-systolic volume itself also serves a similar purpose as a buffer[37]. Thus, the occurrence of heart failure is marked either by an inability of the heart to provide a cardiac output meeting the metabolic demands of the body, and/or a compromised efficiency in function and consequent lack of headroom[38].

There are two forms of heart failure: systolic and diastolic. As the names suggest, systolic heart failure results from the heart's lack of ability to pump blood during systole, while diastolic heart failure results from the heart's lack of ability to fill with blood during diastole.

During systole, the goal of the left ventricle is to eject as much of its blood volume as possible. The ability of the left ventricle to do so can be viewed as a function of three factors: (a) the contractility of the myocardium, (b) the afterload in the left ventricle, and (c) the structural integrity of the left ventricle[38].

(a) The contractility acts as the driving force for blood flow out of the left ventricle and through the aortic valve. If the contractility is compromised, the left ventricle's capacity to eject blood will be reduced. This can occur due to conditions such as dilated cardiomyopathy or ischemic heart disease, which cause the myocardium to contract weakly. Alternatively, it can be a result of pathologies such as arrhythmia, in which the myocardium activates asynchronously rather than produces a single, strong impulse during systole.

(b) Afterload is the amount of resistance encountered by the left ventricle as it attempts to contract. The more resistance the left ventricle encounters, the more difficult it will be to eject blood. This can result from conditions such as aortic stenosis, where the aortic valve does not open fully and restricts the blood flow, or hypertension, in which there is elevated pressure in the left ventricle resisting contraction.

(c) The structural integrity of the left ventricle is a prerequisite for its functions. For example, a mitral valve defect that results in an improper seal between the left ventricle and left atrium can cause blood to backflow into the left atrium during systole, instead of flowing through the aortic valve towards the rest of the body.

During diastole, the goal of the left ventricle is to fill with as much blood as possible. This ensures that there is enough blood available to be pumped out afterwards, during systole. Similar to systole, the diastolic function can also be broken down into three contributing factors[38]: (a) the distensibility of the left ventricle, (b) external compression, and (c) structural factors.

(a) In diastole, the distensibility of the left ventricle serves as the driving force, allowing it to expand and be filled with blood. A stiffer or thicker myocardium loses its ability to expand,

resulting in decreased distensibility. For example, the protein deposits caused by amyloidosis can lead to this effect. Hypertrophy can also be caused by chronic hypertension as a result of the heart working against high afterloads[71]. Gradual loss of distensibility is also a natural result of aging[72].

(b) External compression acts as the resistive force against the distensibility of the left ventricle. External compression restricts the volume of blood that can be filled, leading to diastolic heart failure. External compression is introduced from regions external to the left ventricle, such as the pericardium in constrictive pericarditis and cardiac tamponade. It can even come from the right ventricle, as is the case in cor pulmonale, in which right ventricular failure has excessive volume. This dilation of the right ventricle creates an external compression acting on the left ventricle.

(c) Structural factors can also affect diastole, in the form of obstructions to filling. Obstructions prevent the left ventricle from filling optimally during diastole, leading to a reduction of the end-diastolic volume. For example, mitral stenosis can prevent the mitral valve from opening optimally, reducing blood flow into the left ventricle from the left atrium. A left atrial myxoma located in the way of the mitral valve could also cause a similar effect.

Additionally, diastolic heart failure can also result from systolic heart failure. When the ejection fraction decreases due to systolic heart failure, causing an initial drop in stroke volume and cardiac output, the heart may compensate by increasing the end-diastolic volume with higher filling pressure through neurohormonal pathways that increase the vascular tone and intravascular volume[37,38]. This invokes the Frank-Starling relationship[73] by creating a higher pre-load, allowing the stroke volume to return back to normal levels while the ejection fraction remains low. Because diastolic filling must occur with elevated pressure if the cardiac output is to be maintained, diastolic heart failure can happen in this situation. As with nearly all human physiology, this is one of many examples showing how the final indices that we observe are often the result of a long cascade of interactions.

As such, the numerical indices (i.e., ESV, EDV, heart rate, stroke volume, cardiac output, and ejection fraction) by themselves can only point us towards whether systolic/diastolic heart failure has occurred, or possibly both have occurred. For example, in systolic heart failure, one cannot determine whether it stems from a lack of contractility, increased afterload, or loss of structural integrity, based on the numbers alone. Therefore, while these indices may not provide a specific diagnosis, they serve as highly efficient quantitative indicators for the early detection of a broad range of cardiovascular diseases. The ability to continuously monitor these indices opens the opportunity for more comprehensive examinations to be performed in a timely manner.

1.3 B-mode and M-mode images

In this study, we demonstrate direct ultrasound B-mode and M-mode imaging of cardiac structures. This capability of the wearable imager is valuable in providing a more detailed diagnosis after an initial indication of cardiovascular diseases from the numerical indices. A vast number of factors contributing to heart failure can be identified visually just from imaging the heart continuously. For example, ischemia may result in the death of myocardial cells, producing effects such as fibrillation and hypokinesia/akinesia, which can be easily observed in continuously recorded images. Features related to the structural integrity of the heart and obstructions to filling may also be seen, such as valvular stenosis, valvular regurgitation, septal defects, and left atrial myxoma. Hypertrophic cardiomyopathy can result in an obvious thickening of the myocardium that can be seen on the ultrasound recording, while dilated cardiomyopathy results in a visible thinning of the myocardium and dilation of the left ventricle. Hypertrophic cardiomyopathy can also be differentiated from pathologies like amyloidosis due to the extreme brightness of the amyloid proteins relative to the myocardium on the ultrasound image. These are a few notable examples of the vast range of diseases that can be diagnosed conclusively through wearable imaging.

In this study, four standard B-mode ultrasound views of the heart were implemented: parasternal long axis, parasternal short axis, apical four-chamber, and apical two-chamber. Parasternal long and short axis views are orthogonal, so are apical four- and two-chamber views. Each of these views provides a different perspective of the heart, with its own viewing angle and set of structures, allowing for the most comprehensive heart investigations. In addition, imaging the heart from these standard views allows for a more accurate representation of cardiac structural dimensions. Imaging from other angles can also reveal internal structures, but the displayed dimensions may have deviations from what clinicians normally measure. From the four views, the symptoms related to major abnormalities in cardiac functions such as changes in myocardial thickness, can be easily and accurately observed. Furthermore, diseases and activities may increase the heart rate, leading to faster valvular velocities, which could be quantitatively estimated through a frame-by-frame observation method: the distance of the valve movement can be detected from frames in B-mode images. A faster valvular velocity leads to fewer frames to reach the same distance.

1.4 Unique innovation of the wearable imager

Emerging wearable electronics in recent years have gradually transformed ultrasound technologies from traditional large-scale equipment to miniaturized wearable devices. Wearable ultrasound devices have been demonstrated for other applications, including monitoring of blood pressure, blood flow, and tissue motions (Extended Data Table 1), continuous in-motion analytical B-mode imaging of deep tissues, a major aspiration for diagnostic ultrasound, has not been

achieved[18,19,26,28,32,47,48,74-83.] Recently continuous B-mode imaging on various tissues of the human body was reported based on a rigid ultrasound device[25]. The authors used a soft adhesive material to fix the rigid ultrasound device on the human body and acquired only snapshots of the heart when the subject was in-motion. Moreover, there was a lack of analysis of these acquired images. The data from wearable devices are only valuable when we can extract actionable information from them.

In this work, the ultrasound device is soft and can collect high-quality cardiac images during exercise continuously. We reconstructed the B-mode images of phantoms and various tissues (e.g., liver, abdominal aorta, inferior vena cava, and biopsy process, as seen in Supplementary Figs. 30 and 31) with a particular focus on the heart. Because the heart has a complex and fast-moving anatomical structure, continuous cardiac imaging can provide new opportunities of understanding pathologies of many cardiovascular diseases. We showed bi-planar imaging from multiple standard views commonly used in transthoracic echocardiography examinations. We quantified the displacement of myocardium for the diagnosis of myocardial ischemia. We also used a deep learning model to extract key cardiac metrics such as stroke volume, ejection fraction, and cardiac output from the continuous images automatically. These contributions have never been made by any wearable devices. The novelty of this work is two-fold.

(1) Innovation in device engineering, including array design, microfabrication, and transmitting strategy

First, we used 32 elements in each row of transducers with a pitch of 0.4 mm (shorter than one ultrasound wavelength), which considerably enhanced the signal-to-noise ratio and weakened grating-lobes, leading to better spatial resolutions, stronger penetration capabilities, and less artifacts compared to existing wearable phased array images.

Second, to achieve such a large size and small pitch of the array, new microfabrication techniques were required. We replaced the conventional serpentine-based copper electrodes with straight liquid metal electrodes that were inherently stretchable, and micropatterned the traces with screen printing and laser ablation, which significantly increased the pattern resolution, with a minimum width and kerf of 0.03 mm and 0.025 mm, respectively. Such a high pattern resolution enables stretchable electronics with high-density. Additionally, we replaced the manual alignment of hundreds of elements with a new automatic alignment method, which was more time-efficient and had a higher success rate. Moreover, we applied a room-temperature bonding method and made a dense backing layer to enhance the acoustic performance of each ultrasonic transducer in the array.

Third, for the packaging strategy, in previous work, we used a type of silicone elastomer (Ecoflex-0030) as the substrate and superstrate, which provided mechanical support and waterproof encapsulation to the device[18,19]. In this work, we used a triblock copolymer (SEBS) coated by liquid metal as the substrate and superstrate. This did not only provide mechanical support and waterproof encapsulation, but also serves as the ground electrode and the shielding layer to screen any interference from ambient electromagnetic waves that might otherwise induce imaging artifacts.

Specifically, to apply the SEBS as the encapsulation material, we have tried a variety of polymers and many ways of coating liquid-metal. Liquid metal has a large surface tension and does not easily wet the polymer substrate. We solved this problem by mixing SEBS, dissolved in toluene solution, with liquid metal to form a composite electrode, which could readily wet and adhere to the SEBS substrate. Silicone elastomers did not work because they cannot be dissolved by common solvents so they failed to composite with the liquid metal. This new packaging strategy enables more functions and use cases and has never been realized in any reported wearable ultrasonic devices.

Fourth, an advanced wide-beam compounding transmission method was introduced to wearable ultrasonic technologies for the first time. Compared with other traditional transmission approaches, such as single plane-wave and mono-focus, the wide-beam compounding method provides B-mode images with enhanced spatial resolutions, signal-to-noise ratios, and contrast-to-noise ratios[27].

Based on the above innovations in device engineering, we realized high-quality B-mode ultrasound imaging using a wearable ultrasonic patch. High-quality B-mode imaging-guided applications and techniques, such as Doppler-based imaging[84], elastography[85], 3D/4D imaging[86], ultrafast/super-resolution imaging[87,88], and ultrasound stimulation[89], can all be possible on the wearable platform.

(2) Continuous cardiac imaging during exercise and associated automatic data processing by deep learning

Conventionally, cardiac imaging is carried out using bulky expensive equipment while the subject stays still. Existing wearable health devices allow monitoring the human body on the go but can only capture signals on the skin surface and generate signals in the form of data points and curves. Wearable imaging of central organs (e.g., the heart) remains a grand challenge in the field. Particularly, the heart is relatively difficult to image due to its complex and fast-moving anatomical structure and large depth from the skin.

In this study, we demonstrated continuous cardiac imaging with qualities comparable to those from a commercial ultrasound probe. The innovation of this application is two-fold.

First, traditional stress echocardiography can only image the heart after exercise. Wearable cardiac imaging allows capturing the heart anatomy and performance before, during, and promptly after exercise continuously in real time. This technology enables evaluating cardiac abnormalities that are only present under stress, opening up new diagnostic possibilities with information that previously could not be acquired.

Second, conventionally, measuring cardiac indices such as stroke volume, cardiac output, and ejection fraction usually requires cardiologists to contour the boundary of the left ventricle manually, which is very time-consuming and operator-dependent. They typically collect only one representative cardiac cycle to evaluate those parameters[3], giving only discrete data. Although some research studies[3,90] and commercial systems[91] developed algorithms to derive these indices over multiple cardiac cycles, it still requires cardiologists to operate the imaging probe manually. Long-term continuous cardiac imaging by integrating the wearable ultrasound patch with deep-learning based image-processing can derive waveforms of these indices automatically, with an unprecedented high temporal resolution.

In summary, the innovations in device engineering and automated image processing allow the wearable ultrasonic patch to perform long-term continuous cardiac imaging from multiple views during exercise. The output waveforms of cardiac indices such as cardiac output, stroke volume, and ejection fraction have never been achieved by existing wearables.

### 1.5 Bandwidth improvement of the wearable imager

The 55% transducer bandwidth is not as high as that of the commercial P4-2v imager (~74% bandwidth). In contrast to the rigid ultrasound probe, which has a very thick backing layer to effectively attenuate the reverberation, the wearable ultrasonic patch cannot be assembled with such a thick backing layer because it would severely impair the mechanical compliance of the device, preventing it from intimately conforming and coupling to the human skin. Therefore, the current design of a thin yet dense backing layer is a compromise between reducing reverberation and maintaining outstanding mechanical compliance of the device.

To quantify the efficacy of the thin yet dense backing layer in this study, we performed pulse-echo testing of an element without the backing layer. The frequency spectrum and its -6 dB bandwidth are derived (Supplementary Fig. 1). The results show that the backing layer in this study improved the bandwidth by almost 25%.

In future work, we will develop new composites for backing layers[92,93]. Specifically, we will incorporate glass bubbles and tungsten powders into the polyimide resin. By tuning the proportion of the three components, we can synthesize a composite with a small thickness, an appropriate acoustic impedance, and a high attenuation coefficient that is five times higher than the current material[93]. This new composite will allow the wearable ultrasound probe to have both high bandwidth and skin-like mechanical properties.

**Supplementary Discussion 2: Why wearable and why ultrasound?**

2.1 Why wearable?

Not all diseases have regular and sustained symptoms or pathologies. Continuous monitoring over a long period is essential for providing reliable and accurate diagnosis[3,4]. The insufficient sampling of signals would possibly miss transient but critical signals and thus provide less confidence to the diagnostic results. Thus, long-term monitoring is highly desired for those that are sporadic and barely predictable. However, conventional methods to continuously monitor the heart are invasive or semi-invasive, which are resource intensive and limit their use in bedside surveillance for critically ill or surgery patients[94,95]. Invasive methods may require anesthesia that could influence the measurement results[96,97]. Their invasive nature is also associated with risk of morbidity and mortality[94]. Emerging wearable technologies address these challenges by enabling long-term monitoring noninvasively.

Besides stress echocardiography demonstrated in this work, there are several other use cases that can benefit from wearable long-term monitoring.

Sinus arrhythmia produced by the irregular release of impulses from the sinoatrial node is one typical example[98]. Being prevalent among senior people[99], arrhythmia is the root cause of a variety of other symptoms and diseases, such as blood clotting in the heart, hypotension that leads to dizziness, and sudden death. Sinus arrhythmia includes sinus tachycardia, bradycardia, and arrest[100]. Sinus arrhythmias may not have symptoms during the limited examination time because it could be sporadic and barely predictable in daily life[101]. Hence, prolonged tracking time can help record the timing and syndromes for a better diagnosis. Also, while it is simple to detect symptoms with a quick test, it is imprudent to instantly diagnose signs as illness and intervene immediately. This is because sinus rhythm is very sensitive to factors such as mood and respiration, and after a while the variations automatically revert to normal[98]. Existing approaches for monitoring arrhythmia are plagued by various problems. Auscultation of the heartbeat with a stethoscope is a general but nonspecific measurement, because abnormity like premature or abnormal beats do not always produce an audible pumping and may be missed and misdiagnosed[102]. An electrocardiogram could be more effective and accurate. It provides abundant information because different types of arrhythmias have different reflections on electrocardiograms. Doctors can tell

the type from an electrocardiogram directly and thus more quickly make a move. But motion artifacts introduced by body movement will distort electrocardiogram signals badly, which challenges signal accuracy in daily monitoring[103]. Additionally, the lack of structural information prevents electrocardiograms from providing a comprehensive diagnosis. Photoplethysmography fails to provide sufficient information for further diagnosing the type of arrhythmia[104], because it can only monitor the heart rate. There are some studies on building mathematical models to correlate photoplethysmography waveforms to the arrhythmia type, but the result is not accurate enough[105]. Other invasive methods may be less commonly used[106].

Another example is paroxysmal atrial fibrillation, which intermittently occurs as a disorder of cardiac rhythm[107]. This disease also suffers from the same problem that limited examination time may not be enough for a thorough diagnosis, and this could introduce horrible results. Paroxysmal atrial fibrillation may develop into chronic atrial fibrillation, which occurs more frequently, a direct result of misdiagnosis. Moreover, a common error in clinical management of atrial fibrillation is to treat chronic sustained atrial fibrillation and paroxysmal atrial fibrillation similarly, despite some differences in management objectives[107]. Atrial fibrillation is often associated with a high risk of morbidity and mortality from heart failure, stroke, and thromboembolic complications[107,108]. Thus, the failure of detecting the early stage of paroxysmal atrial fibrillation is disastrous.

Similarly, coronary heart disease may also be undetectable in a transient test. Coronary heart disease is caused by plaque built-up near the coronary arteries, which limits the blood supply[109]. Coronary heart disease often develops over decades and is frequently ignored because no symptoms are detected. However, the unnoticed coronary heart disease could lead to an acute heart attack due to the blockage of the artery, which is extremely lethal and results in a high mortality[109]. This heart attack caused by undetected coronary heart disease with no obvious symptoms may also be called a “silent heart attack”. Silent precursors to an imminent myocardial infarction can be detected by observing segmental wall motion abnormalities in ultrasound images.

Finally, acute heart disease could barely be noticed when it is not occurring. Myocardial infarction is the lack of blood supply to the myocardium, which weakens cardiac activities[110]. Myocardial infarction has a high morbidity because many factors and triggers contribute to it, such as alcohol, physical exertion, and obesity. Myocardial infarction also has a high mortality. It cannot be effectively detected in current measuring procedures, because the short period of testing time cannot capture any signs of it unless it is occurring with symptoms.

We can summarize that previous efforts of wearable devices for non-invasive heart monitoring are mainly categorized into three fields: electrical probe, electromagnetic probe, and mechanical probe[51]. Specifically, the electrocardiogram sensor for local-field potential recording for analyzing cardiac rhythm[12,111]; the electrical impedance tomography sensor based on internal electrical

conductivity mapping mainly for distinguishing systole and diastole phases[16]; the electromagnetic sensor that measures stroke volume by relating the resonant frequency response of the sensor to permittivity changes caused by blood volume[112]; the passive vibration sensor based on mechanical waves usually designed for monitoring the heart rate[113]. However, those signals either are indirect or suffer from low spatial resolutions, from which many clinically important cardiac characteristics, such as the volume of heart chambers, the myocardium structure, and the ventricular ejection function, cannot be visually and accurately evaluated[114].

## 2.2 General requirements of imaging the heart

Echocardiography, as one of the most flexible diagnostic tools revealing the structural information of the heart, is widely adopted in hospitals[115]. However, current echocardiography focuses on short-term recordings of the heart to make a diagnosis, but transient symptoms or dysfunctions may not appear during the limited time of such recordings. Other methods based on imaging can barely provide reliable and thorough monitoring of cardiac functions, including magnetic resonance imaging[116], computed tomography[117], single-photon emission computed tomography[118], positron emission tomography[119], and optical coherence tomography[120]. Here we briefly outline the pros and cons of each method's working principles and the rationale behind selecting the ultrasound platform in this study (Supplementary Table 1). We desire a device that is wearable, can target the heart, and is capable of 2D or 3D imaging with a high spatial resolution and sufficient contrast. These requirements can be summed up in a single term: wearable heart imaging, which entails the following needs.

For a modality to be suitable in wearable devices, it must first be able to be packaged into a small, lightweight, and minimal form factor that is both comfortable and non-invasive. The device must ideally provide zero hindrance to the wearer, such that there is a negligible impact on their comfort or normal activities when wearing it. A sufficient penetration depth is required to target the heart non-invasively. Furthermore, the chosen modality must be robust, with no degradation in the image quality over time or large variances in image quality under different conditions encountered in daily life. Wearable devices are often desired to perform long-term monitoring, and to be frequently removed and reapplied to the wearer. Therefore, the device must also withstand the wear and tear of long-term use without a loss in image quality over time. Most importantly, the device must be safe for indefinite use–for example, the modality should not be based on ionizing radiation.

For a modality to target the heart, the major challenge to overcome is the high temporal resolution required. The heart may beat at rates of anywhere from 50 beats per minute at rest[121] to nearly 200 beats per minute during exercise[122], and as such, requires a modality that can achieve temporal resolutions of at least 33 ms[5,123] to image continuously in real-time. Modalities that lack

sufficient temporal resolutions can result in the lack of critical diagnostic information, as well as degradation of image quality due to motion artifacts.

Lastly, to make a device useful for performing a wide range of general examinations, the modality must be able to image in 2D or 3D with high spatial resolutions and good contrast. Most of the work on wearable devices has primarily targeted measures such as pulse acquisition and blood oxygen levels. While these are helpful signals, they cannot compare to the immense diagnostic value that direct 2D or 3D imaging of the heart provides. Imaging provides a great wealth of information, but its quality is crucial to being able to make these diagnoses. Poor image quality may often obscure key indicators of diseases, lead to false positives, or make it difficult to distinguish between different conditions.

Therefore, to summarize, the ideal modality should be robust, safe for long-term use, easily scaled down to wearable and portable form factors, imaging in 2D or 3D with high spatial/temporal resolutions, having a sufficient penetration depth to target the heart non-invasively, having a sufficient contrast, and having a high signal-to-noise ratio. However, it should be noted that some of these requirements have trade-offs. For example, by nature, imaging at high spatial resolutions will tend to sacrifice the temporal resolution, due to the larger amount of data being acquired and processed. Therefore, we can only select the most suitable modality that is able to balance all factors while meeting sufficient requirements.

## 2.3 Magnetic resonance imaging

Magnetic resonance imaging works by using a powerful magnetic field to align the protons in the body’s tissues with the field. Radiofrequency waves are then pulsed to disturb this alignment, followed by a subsequent release of secondary radio waves when the protons realign with the field. These signals are collected to form images[124]. Magnetic resonance imaging provides high tissue contrast and image quality in real-time with spatial and temporal resolutions in the range of 1.6 mm and 13-50 ms[5,125], respectively. In addition, it is non-ionizing because it uses a magnetic field and radiofrequency waves. However, magnetic resonance imaging has several obvious drawbacks that make it unsuitable for needs in this study. First, magnetic resonance imaging machines are extremely bulky and expensive and are not scalable to wearable form factors. Furthermore, the powerful magnetic field is an intrinsic requirement of the modality and can easily present a significant hazard in everyday life in addition to being incompatible with devices like pacemakers that are likely to be used by the target demographics in this study.

## 2.4 X-ray computed tomography

A computed tomography scanner consists of an X-ray source and a detector placed opposite of each other, which rotate around the subject to capture several images from multiple angles. The images are then used to reconstruct a 3D image of the subject[126]. Although computed tomography

is relatively low cost and provides high spatial resolutions of around 0.3 mm[6], the scanner contains many moving parts. This type of moving design is not suitable for wearable devices, and the physical constraint puts a strict limitation on the temporal resolution (~66 ms[6,127]) that makes it unsuitable for real-time cardiac imaging. Furthermore, the ionizing X-ray radiation makes it fundamentally unsafe for long-term wearing.

### 2.5 Emission tomography

Single-photon emission computed tomography and positron emission tomography make use of radiotracers injected into the body that radioactively decay over time as they travel through the body following their designated molecular targets. These signals can then be collected using a gamma camera to quantify those molecular targets and metabolic events[128-130]. This allows gathering unique types of information such as myocardial perfusion and cell metabolic activities[129]. In addition, positron emission tomography's temporal resolution is too slow (>2000 ms[131]) for cardiac imaging, while single-photon emission computed tomography's poor image resolution (~10 mm[132]) makes it largely impractical for more general diagnostic imaging. There is also a very low signal-to-noise ratio in these modalities[130]. Subjects are exposed to low levels of ionizing gamma radiation, and the tracers must be replenished over time as they decay, making these modalities unsuitable for wearable devices.

### 2.6 Optical coherence tomography

Optical coherence tomography uses light scattering within the 700-900 nm wavelength range, also known as the "therapeutic window", to image the human tissue[8]. These wavelengths have low absorbance and high scattering in tissues on top of being non-ionizing, making them useful for imaging tissues. In constructing the image, the time delay cannot be used to determine the location of the scattered signals because the speed of light is too fast. Thus, interferometry is used instead, in which the primary beam of light is split in half to target the tissue and a mirror simultaneously. As the distance of the mirror is varied, constructive interference occurs with signals coming from different locations within the tissue, allowing the position of the signals to be distinguished. Due to the short optical wavelength, optical coherence tomography can achieve extremely high spatial resolutions of up to 1 μm[8], and temporal resolutions of 2 ms[8]. In addition, dyes can be injected into the subject to add additional capabilities to optical coherence tomography. For example, the use of voltage-reactive dyes can make it possible to form activation and conduction velocity vector maps of the myocardium using optical coherence tomography[8]. Despite these advantages, optical coherence tomography is unsuitable due to the bulky optics required, and limited tissue penetration depth of 2-3 mm[8].

### 2.7 Why ultrasound?

Lastly, we look at the rationale for selecting the ultrasound modality in this study. Piezoelectric transducers generate ultrasound waves throughout the tissue. The same transducers

can then collect the backscattered signals and construct an image based on their strength and time delay at each pixel[27,133,134]. This “all-in-one” capability of ultrasound allows the device to become extremely compact and easily scaled down to wearable form factors[26]. Ultrasound waves have low absorption and backscattering and thus can penetrate over decimeters in the integumentary and musculoskeletal systems of the human body. Ultrasound has been able to achieve temporal resolutions of less than 1 ms[135]. The spatial resolution of ultrasound increases with the frequency. However, this comes at the cost of penetration depth as higher frequencies are more strongly attenuated by tissues[134,136]. In general, most conventional diagnostic ultrasound devices use sound waves in the range of 2-18 MHz[134] and can achieve spatial resolutions in the range of 0.4-2 mm[137]. Additionally, even for long-term use, ultrasound has largely been considered fully safe as long as the power output is kept at the minimum levels needed[138], below the safety threshold defined by FDA ($I_{SPTA} \leq 720$ mW/cm$^2$, $I_{SPPA} \leq 190$ W/cm$^2$)[139] . The mechanical energy itself is not toxic to the body.

In the case of arrhythmia, a wearable device with live B-mode and M-mode images is perfect for several reasons. First, heart beats are directly recorded and visualized in echocardiography by the wearable device, resulting in a high accuracy in calculating heart rates from B-mode videos or M-mode images. Second, arrhythmia is very unpredictable and may only happen when the heart is under high loads. The wearable device supports uninterrupted long-time monitoring in daily life and can capture any irregularity in heart beats. Third, the abnormal cardiac structure causing arrhythmia can be easily detected in B-mode images, which visualize the root cause of the disease and inform better therapeutic decisions. For example, the gross dilations of the right atrium and right ventricle are considered as an important indicator of arrhythmia[140].

The main drawback of ultrasound is that there is inherently a lot of noise in the signal, due to random scatterers in the tissue[141]. However, significant algorithmic progress has been made in this area to improve the image quality of modern ultrasound scanners. Especially given its versatile functions in clinical applications, ultrasound still remains extremely viable, as well as the best option as a wearable modality for imaging the heart.

As of the time of writing, there have been no other studies that have produced, specifically, wearable ultrasound heart imaging devices. A wearable device was made to target the heart[142], but did not have imaging capability. Therefore, the wearable heart imaging capability introduced in this study addressed a critical unmet need.

### 2.8 Comparability of the wearable ultrasound to clinical ultrasound

Traditional ultrasound probes require either constant holding and/or repositioning of the probe each time before taking a measurement. The wearable imager reduces the user dependency down to a single placement of the probe at the start, after which no operator is necessary because the

patch remains adhered in place. Furthermore, in regions where sonographers are a scarce resource, such an advantage could allow a single sonographer to attend to multiple patients at a time, as they would not have to physically hold the probe for each patient.

In spite of these advancements, the wearable imager is not fully immune to user-dependency either. Because ultrasound cannot penetrate bone, an ultrasound probe must always be correctly positioned such that the region of interest is within the imaging window with a clear line of sight unobstructed by bones. This user-dependence can be further minimized by 3D imaging or 2D plane-steering, which is an effective way to eliminate the user-dependency.

The images from the wearable imager, while comparable to the commercial probes, are not at the same level as those taken by a cardiologist using a clinical machine. The models of the commercial probe and the driving system we used were P4-2v and Vantage 256 from Verasonics company, which is widely known for manufacturing cutting-edge research ultrasound systems. Nonetheless, the Verasonics system performance is still not as good as clinical ultrasound systems manufactured by General Electric or Philips, which excel in designing ultrasound transmitting and receiving modes and customizing image post-processing algorithms for each imaging target. We acquired cardiac images taken by an experienced cardiologist using a clinical machine in the hospital (Supplementary Fig. 32).

However, those clinical ultrasound systems have fixed adaptors and algorithms that cannot be adapted to driving the wearable imager in this study. Therefore, we used Verasonics, a programmable research platform, to drive the patch. In Figure 3a, we compared B-mode cardiac images tested by the wearable imager and the commercial P4-2v probe. Those two devices were controlled by the Verasonics Vantage 256 machine with the same transmitting mode and image post-processing algorithms. In this case, we could make a fair performance comparison between the wearable imager and the commercial P4-2v probe.

The size and cost of the back-end hardware can be readily reduced by replacing it with a printed circuit board used for the commercial portable ultrasound probe. We will work on prototyping a printed circuit board with pulsing, receiving, and data transmitting units and integrating it with the wearable ultrasound imager. The printed circuit board will have a miniaturized footprint. Additionally, the entire system can be powered by a lithium-polymer battery with a voltage of only 3.7 V. The voltage can be amplified up to 200 V by a voltage regulator. We will eventually prototype the printed circuit board in a compact and multilayered structure and make it flexible or even stretchable.

Furthermore, the mechanical index, spatial resolution, and contrast-to-noise ratio can be further improved by using single crystal piezoelectric materials, adding matching layer and lens,

increasing the number of elements, implementing harmonic imaging, and shrinking the pitch further to eliminate the artifacts. The near-field is excessively bright and can be improved by suppressing the ring-down with backing layer materials of high attenuation coefficients. The sector angle of the sonographic window is limited and can be improved by shrinking the array pitch so that the ultrasound beam can steer to a large angle without generating grating lobe-based artifacts. New algorithms will be explored to maintain a high frame rate while at an expanded sector angle. We could also fabricate a large wearable imager so that it covers multiple positions simultaneously. This large array will eliminate the need to repositioning when imaging different sites. To achieve that, we will have to develop a new wiring strategy to individually address each transducer element in the large array.

**Supplementary Discussion 3: Configuration of the array**

3.1 The configuration of the array in this study

The device is composed of two orthogonal 1D arrays, which can be separated into two parts: four arms and a central area (Extended Data Fig. 1). Each arm consists of 13 elements, with each element containing a row of 6×1 sub-elements. The 6×1 sub-elements are shorted and controlled as one channel. We divided one element into 6×1 sub-elements to considerably improve the stretchability of the device, which could conform more intimately to the curved human skin and provide a better interfacial coupling condition. The central area consists of 36 individually addressable sub-elements, whose activation can be configured through customized algorithms as 6×1 elements in either arm of the array. Because the array pitch is less than one wavelength of the ultrasound wave, the device can be considered as two phased arrays with 32 elements in each.

The 88 channels were physically plugged into the control system, but not all of them were activated all the time. The activating sequence was controlled by the customized algorithm to reconstruct two sectorial images sequentially. Specifically, the 32 elements in one direction of the orthogonal array transmit and receive ultrasound waves first. After a sectorial B-mode image has been reconstructed, the system deactivated this direction and switched to controlling the other direction of the orthogonal array to form the second image. Different from how traditional 2D matrix probes work, this method is limited to only one pair of cross planes (one along each phased array; Extended Data Fig. 1). The 2D matrix probe reconstructs a whole 3D image first, and then extracts any pairs of cross planes from the whole 3D image. In other words, this method captures extra unneeded data, which is more time-consuming, leading to a lower frame rate than the method in this study. Because these standard echocardiographic views are orthogonal to each other and the other cross planes in between are of less interest, our method optimizes the frame rate without losing key data.

However, the disadvantage is that our method cannot do 3D imaging nor capture all cross planes from a single probe position. To overcome these disadvantages, our future work will focus on developing a wearable 2D matrix probe that can achieve 3D imaging and thus extract any pairs of cross planes.

### 3.2 The Mills cross array

The configuration of Mills cross arrays[143-146] is almost the same as that of our orthogonal array (Supplementary Fig. 34): two linear or phased arrays in orthogonal orientations with the central elements shared by two arrays. The three major differences between them are (1) the Mills cross array was rigid and the orthogonal array is stretchable; (2) the Mills cross array can reconstruct both 2D and 3D images[147], while our device can only conduct imaging in two 2D planes; (3) the manner of ultrasound transmission and receiving are different, as detailed in the following.

In the Mills cross array, one array serves for transmitting beamforming in the elevational direction and the other for receiving beamforming in the azimuthal direction (Supplementary Fig. 34). In this way, a 2D image can be reconstructed with focusing in the elevational direction. However, this method has a major limitation: the effective aperture in the azimuthal plane is very small (determined by the shared center of the cross array). Such a small aperture diminishes the reflected signals that go to the receive array, resulting in a low signal-to-noise ratio, and degrades the lateral resolution in the azimuthal direction, causing low-quality of reconstructed images[145]. On the contrary, performing the transmit and receive beamforming using one common phased array will not have this problem due to its long effective aperture in the azimuth plane, yielding 2D B-mode images with high qualities in this work.

### 3.3 The 1.5D/1.75D array

We did not create 1.5D or 1.75D arrays either because of three strong reasons. First, a 1.5D or 1.75D array will need more electrodes. Specifically, the 1.5D and 1.75D arrays would include 104 and 260 additional electrodes compared to the current probe, respectively[20]. This design configuration would require fabricating more than 10 layers of electrodes, which would make the top electrode excessively thick and compromise the device's mechanical compliance. Second, it would significantly lower the imaging frame rate as it would involve an additional beamforming process in the elevational direction. The frame rate is crucial for cardiac imaging because of the highly dynamic cardiac movement. A low frame rate reduces the number of data points in the left ventricular volume waveform, causing aliasing of critical features in the waveform of cardiac cycles, such as diastasis and isovolumetric contraction (Fig. 5b right). The outcomes are inaccurate stroke volume, ejection fraction, and cardiac output. The frame rates for 1.5D or 1.75D arrays are typically <10 Hz[148-150], which could not support high-fidelity waveform extraction from the video. Third, it is highly likely that there will be a strong grating lobe in the elevational direction due to the transmitting beamforming implemented by a small number of elements, which can induce

artifacts into the images[20]. Therefore, this work focuses on achieving cardiac 2D B-mode images using 1D phased array.

There is no focusing effect in the elevational direction for the orthogonal array in this work, which may diverge ultrasonic energies transmitted to deep tissues. To mitigate this problem, we used an advanced compounding transmission approach and high transducer performance with a large electromechanical coupling coefficient in this work to maximize the transmission and reflection energies in the azimuth and axial directions. This strategy compensates for the energy loss due to the lack of the elevational focusing, resulting in bright and clear cardiac images even at a depth of 14 cm. To further improve the elevational resolution, we will replace the linear arrays with 1.75D or 2D array[20] in future studies.

**Supplementary Discussion 4: Possible sources of electromagnetic interference**

The noise in the ultrasound signals had such an ultra-wide bandwidth that the most likely source was radio communication signals. The electromagnetic wave frequencies beyond the ultrasound signals (1-5 MHz) in this study could be removed by applying digital filers to the received signals. For electromagnetic wave frequencies within this range, e.g., the amplitude-modulated radio and short-wave radio, they could not be removed by digital filters but could be mitigated by adding a shielding layer. For the latter, we could attribute the noise to the following two possible sources:

(1) Electromagnetic noise received by the electrodes or transmission lines directly from the ambience[151]. Without the shielding layer, bare transmission lines and the electrodes can act as an antenna to receive those ambient electromagnetic waves.

(2) Electromagnetic noise coupled from the human body[152]. We observed an increasing noise amplitude when the device got close to the human body, indicating an electrical field approaching the human body. When electromagnetic waves pass by the human body, they will generate magnetically-induced electrical potential in the human body according to the Faraday's law of electromagnetic induction. In this case, the human body can be considered as a circuit that generates an additive electrical field to the ambient electromagnetic waves. Even though the conductivity of various parts of the human body is low compared with metal conductors, some parts of the human body can still form a loop and generate electrical potential. The potentials can be coupled to device signals as noise[153].

**Supplementary Discussion 5: Measurement variations of imaging metrics**

Taking the measurements of axial resolutions as examples, there were cases where the axial resolution of wearable probe was better than that of commercial probe due to the variation in measurements (position of the probe, rotation of the probe, etc.). Because the surface of the commercial probe is not flat, the degrees of freedom of the commercial probe will be five, as indicated by the red arrows in the Supplementary Fig. 18. The degrees of freedom of the wearable probe will be three, as indicated by the red arrows in the Supplementary Fig. 18. The variations can be introduced in many forms:

1) When the commercial probe is tilted in the side view (Supplementary Fig. 18a Side view), or the commercial and wearable probes are rotated in the top view (Supplementary Fig. 18 Top views), the cross section of the filament is enlarged (from a circle to an ellipse). This will cause a longer radiofrequency signal, similar to the effect of reduced bandwidth. A longer radiofrequency signal will increase the size of the bright region generated by each filament target, causing a worse axial resolution.
2) When the commercial and wearable probes are moved laterally in the front view (Supplementary Fig. 18 Front views), or the commercial probe is tilted in the front view (Supplementary Fig. 18a Front view) the filament targets will be off-axis from the center. Then the ultrasound beam intersecting the targets will be more diverged compared to the center beam. The diverged beam has a greater tendency to collect more signals from neighboring targets. This will also lead to a seemingly longer radiofrequency signal. As a result, this kind of positioning will also increase the bright region generated by each filament target and hence worsen axial resolution.
3) When the commercial and wearable probe are moved laterally in the side view (Supplementary Fig. 18 Side views), the probes get closer to the phantom boundary. The closer the probes are to the boundary, the higher the signals from the boundary are. Then the background of the whole image gets brighter. And it is possible that the background plus the noise in the radiofrequency signal will give a background gray value greater than half of that of the deep filament targets (the deeper the target, the dimmer the pixels in the image). This will lead to a higher pixel count, which leads to longer physical distance (Equation 2, Methods), resulting in a worse axial resolution according to the discussion on characterization of the wearable imager in the Methods section.

To reduce the impact of measurement variations, we repeated the same test five times. The axial resolutions from each test are shown in Supplementary Fig. 18. Even though the wearable imager sometimes shows a better axial resolution, the mean axial resolution of the commercial imager is superior due to its higher bandwidth. The error bars for all measurements in Figs. 2d, 2e and Supplementary Fig. 17 show the standard deviation of the measurements.

**Supplementary Discussion 6: Imaging procedures**

### 6.1 Imaging from different views

Four standard positions/orientations were used to obtain the best B-mode images of the heart. The first position was on the left side of the sternum, between the second and fourth intercostal spaces. A linear array device was pointing to the right shoulder. From this position, the imager could inspect the parasternal long axis view of the left ventricle. By rotating the device 90 degrees counterclockwise at the same position, with the device pointing to the left shoulder, a parasternal short axial view of the left ventricle could be obtained. The second position was between the fourth and fifth intercostal spaces. With the linear array device pointing to the left shoulder, the four chambers of the heart could be observed from the apex in this view, also known as the apical four-chamber view. By rotating the device 90 degrees counterclockwise at the same position and aiming towards the right shoulder, the device revealed the left ventricle, left atrium, and mitral valve, i.e., apical two-chamber view.

After the transducer received the response echoes that carry the location and anatomic information of the heart, the echoes were demodulated, decimated, and compressed logarithmically to eventually generate the B-mode image. A graphical user interface for real-time phased array imaging was made up of display windows and control panels for customized settings (Supplementary Fig. 33).

In the apical four-chamber view, all four chambers could be seen simultaneously, so that ventricular interdependence and septal wall abnormalities between the chambers (e.g., in cor pulmonale) could be assessed. We could also measure left and right atrial lengths and areas, as well as right ventricular diameter, length, and area.

Apical two-chamber view could be used to measure the left atrial length and area.

In the parasternal long axis view, detectable structures include the left atrium, left ventricle, mitral valve, aortic valve, interventricular septum, right ventricle, and left ventricular outflow tract[154]. The unique orientation of this view allows visualizing the full length of the mitral and aortic valve leaflets during their closure and excursion, which makes it especially useful for evaluating valvular functions. Measurements taken in the parasternal long axis view include the interventricular septum end-diastole thickness, the left ventricular internal diameter end-diastole (LVIDd), left ventricular internal diameter end-systole (LVIDs), and left ventricular posterior wall dimensions. Among the measurements, the LVIDd and LVIDs are especially valuable because they are correlated with the left ventricular volume. By increasing the imaging depth, the pericardial and pleural spaces can also be seen from this view.

The parasternal short axis view was particularly useful for evaluating the left ventricular wall motion. To do so, we adopted rings divided into a total of 17 segments to assist the mapping of local pathologies. The 17-segment model is a standard tool used in transthoracic echocardiography procedures. In the 17-segment model, the entire left ventricular wall is projected onto a 2D plane, forming a circular projection area made up of multiple concentrically nested rings with varied diameters. Each ring represents the myocardial wall from a different level of the left ventricle, corresponding to the decrease in the diameter of the left ventricular wall from base to apex (viewed from the parasternal short axis). The rings are further divided into a total of 17 segments, each of which receives its blood supply from a different coronary artery. Segmenting the left ventricular wall in this way provides a useful localization mapping that allows abnormal myocardial strain in any given segment, due to cardiac malfunctions such as ischemic heart diseases or myocardial infarction, to be traced back to its corresponding coronary artery of origin.

In the short-axis plane, the circular cross-section of the left ventricle was captured (the basal, mid-cavity, and apical views/slices), and the contractility and distensibility were accessible as the motion of the walls was along the plane. The basal slice captures the ring with the largest diameter (segments 1-6). The mid-cavity slice captures a smaller diameter ring nested inside the basal view (segments 7-12). Likewise, the apical slice is nested inside the mid-cavity slice (segments 13-16). Segment 17 is the apex. Therefore, the relative changes in the cross-sectional diameter, along with the uniformity of wall motions, could be easily assessed. Using these assessments, we can identify the specific segment of the left ventricular wall as pathological.

To quantitatively evaluate the segment displacement, we set a target area for each segment to facilitate further processing. We blurred the original images in the area to reduce the impact of speckles on the feature refinement, and then computed the edge information using a Canny operator based on the blurred image. By indexing the edge in the binary map, the myocardium displacement could be recorded. With these measurements, we could monitor the potential risk factors for myocardial infarction or akinesia, precisely localize ischemic heart diseases, and easily assign hypokinetic or akinetic regions of the left ventricular wall to their governing coronary arteries by tracking the relevant myocardium displacement[155].

### 6.2 Phase correction on nonplanar surfaces

Applying phase correction allows the wearable imager to cover a more diverse population, including obese people. Also, the phase correction is critical for providing accurate cardiac monitoring. Because we used a wide-beam compounding transmission in this study, the delay calculation for the aperture could vary a lot from traditional plane-wave compounding transmission[27,156]. As an intrinsic feature of wide-beam transmission, the focal point of the aperture was set at the opposite side to the imaging area, with the distance between the focal point and the center of the aperture kept constant during the transmission (Supplementary Fig. 13).

Assuming that a linear array of the wearable imager was attached to a planar surface, the transmission and time delay could be calculated following the general approach. Considering the center of the linear array located at $(0,0)$ by default, we can define the distance between the focal point and the center of the aperture as $df = \sqrt{x_{focal}^2 + z_{focal}^2}$, in which $z_{focal} < 0$. Also, the location of the $i^{th}$ transducer could be defined as $(x_i, 0)$, where $x_i = (i - 16.5) \times pitch$ because the device had 32 transducers in each imaging plane. Additionally, we defined the aperture size as $A$ and the angle departure from the vertical direction as $\theta$, where $\theta > 0$ when $x_{focal} > 0$. For a given position of a pixel $(x_p, z_p)$, the time of the wavefront to go to the pixel since the earliest trigger on the transducers was:

$$t_{\theta,p}^{e} \approx \frac{\sqrt{(x_p - df \times \sin\theta)^2 + (z_p - df \times \cos\theta)^2} - dt}{c} \quad (13)$$

where $dt = df \times \cos\theta$ when $|df \times \sin\theta| < \frac{A}{2}$ and $dt = \sqrt{(df \times \sin\theta - sign(\theta) \times \frac{A}{2})^2 + (df \times \cos\theta)^2}$ when $|df \times \sin\theta| \geq \frac{A}{2}$. And the time for the reflected wave to get back to the $i^{th}$ transducer was:

$$t_{i,p}^{r} = \frac{\sqrt{(z_p - z_i)^2 + (x_p - x_i)^2}}{c} \quad (14)$$

The total time delay since the trigger should be

$$t_{i,\theta,p}^{dt} = t_{\theta,p}^{e} + t_{i,p}^{r} \quad (15)$$

where $c$ was the speed of sound that we assumed to be constant in the medium. Considering the delay differences among transducers, the delay of trigger of $i^{th}$ transducer was:

$$t_{i,\theta}^{t} \approx \frac{\sqrt{(df \times \sin\theta - x_i)^2 + (df \times \cos\theta - z_i)^2} - dt}{c} \quad (16)$$

As a result, the delay of the $i^{th}$ transducer for beamforming at a given pixel was:

$$t_{i,\theta,p}^{total} = t_{i,\theta,p}^{dt} - t_{i,\theta}^{t} \quad (17)$$

since each channel started to receive data after transmission.

When the device was placed on a nonplanar surface, the time delay formula above was no longer valid (Supplementary Fig. 15). Assume the curvature radius of the nonplanar surface was $r$. The previous location the $i^{th}$ transducer would then move to $(x_i', z_i')$, and

$$x_i' = sign(x_i) \times r \times sin\,\varphi \quad (18)$$

$$z_i' = r \cdot (1 - cos\,\varphi) \quad (19)$$

where $\varphi = \frac{x_i}{r}$.

With the phase correction, the time of the wavefront to go to the pixel since the earliest trigger on the transducers would be changed to

$$t_{\theta,p}'^{e} \approx \frac{\sqrt{(x_p - df \times sin\,\theta)^2 + (z_p - df \times cos\,\theta)^2} - dt'}{c} \quad (20)$$

where $dt' = \sqrt{df^2 + r^2 + 2 \times df \times r \times cos\,\theta} - r$ when $|\varphi| < \frac{A}{2r}$ and $dt = \sqrt{(df \times sin\,\theta - sign(\varphi) \times |x_1'|)^2 + (df \times cos\,\theta - ssign(\varphi) \times |z_1'|)^2}$ when $|\varphi| \geq \frac{A}{2r}$.

Meanwhile, the time for reflected waves to get back to the $i^{th}$ transducer was changed to

$$t_{i,p}'^{r} = \frac{\sqrt{(z_p - z_i')^2 + (x_p - x_i')^2}}{c} \quad (21)$$

and the delay of triggering the $i^{th}$ transducer was changed to

$$t_{i,\theta}'^{t} \approx \frac{\sqrt{(df \times sin\,\theta - x_i)^2 + (df \times cos\,\theta - z_i)^2} - dt'}{c} \quad (22)$$

Finally, the delay of the $i^{th}$ transducer for beamforming at a given pixel after phase correction became:

$$t_{i,\theta,p}'^{total} = t_{\theta,p}'^{e} + t_{i,p}'^{r} - t_{i,\theta}'^{t} \quad (23)$$

In the receive beamforming, the value of each pixel could be computed as:

$$I_p = 20log_{10}\sum_{\theta=\theta_{min}}^{\theta=\theta_{max}}\sum_{i=1}^{\#\ of\ channel} RF_i(t'^{total}_{i,\theta,p} \times f_s) \quad (24)$$

where $RF_i$ is the radiofrequency signal collected for the $i^{th}$ transducer and $f_s$ is the sampling frequency. Any value larger than 255 would be cut off to adapt to the 8-bit display.

In Extended Data Fig. 2, for the images labeled with "Planar", we fixed the probe on a planar glass slide and used ultrasound couplant to compensate the gap between the probe and the skin. The images labeled with "Curved" were obtained with the probe naturally laminated on the curved skin surface. Exemplary images of the parasternal long axis view were collected on an obese subject, whose curvature radius at the imaging site was ~82 mm. The size and shape of the left ventricle were obviously distorted without the phase correction. The measured values of the stroke volume, cardiac output, and ejection fraction tend to be smaller pre-phase correction than the results post-phase correction, due to the distorted size and shape of the left ventricle.

### 6.3 Validation for long-term use

Motion artifacts plague ultrasound imaging[157-160]. To verify the performance of the wearable imager under daily circumstances, we attached the device to subject's chest and tested the imaging quality under different postures. In the experiments, there was no obvious deterioration of imaging quality, due to the intimate contact of the device to human skin (Extended Data Fig. 4), attesting the stable performance of the device.

We compared the imaging results along the four standard views with ultrasound gel and silicone as the couplant (Supplementary Fig. 20). The results showed that the contrast between different structures and details in the heart anatomy were well conserved when the couplant was changed from ultrasound gel to silicone. We recycled the device and repeated the tests on ten subjects and obtained reliable and reproducible results (Supplementary Fig. 24).

In addition, the biocompatibility of the device was also a potential concern for long-term use, which may adversely irritate the skin. We attached the device to the subject's chest with a commercial adhesive (e.g., Tegaderm) for 24 hours (Supplementary Fig. 22), during which the skin experienced all kinds of scenarios, including exercising and showering. After removing the device, no allergy was observed. The major issue was the reddish area around the device attachment location caused by peeling off the Tegaderm adhesive.

### 6.4 Simultaneous measurements of M-mode images and electrocardiogram

At the beginning of the test, the wearable imager was attached to the parasternal site to image along the parasternal short axis view. We triggered the recording of B-mode images and electrocardiogram (by a commercial device, Cyton Biosensing Board 8 channels) simultaneously. With the concurrent ending of the recording, the time (i.e., frame number in this case) of B-mode images and electrocardiogram could be aligned. Furthermore, we plotted the M-mode image by selecting pixels in one line in the B-mode images and correlating this array of pixels to the frame number.

## Supplementary Discussion 7: Stress echocardiography

### 7.1 Significance

Under a steady state of a healthy subject, the left ventricular volume changes consistently, so the stroke volumes, cardiac outputs, and ejection fractions (three of the most significant indicators of cardiac functions) do not vary significantly[161]. However, the heart state may change frequently and could be extremely intricate at times. Abnormal fluctuations exist in estimating the cardiac function of patients with heart diseases, and the symptoms could be very unpredictable, which also incur dramatic changes in the heart in a relatively short time. Symptoms of cardiovascular diseases like myocardial ischemia only emerge under stress, where the induced wall motion abnormalities are more noticeable[162]. Therefore, stress echocardiography is carried out. Valvular pathologies are easily observed by individually tailored stress echocardiography[163]. In addition, stress echocardiography is valuable for studying pulmonary circulation[164]. Stress echocardiography can also help determine limits for safe exercise before starting a cardiac rehabilitation program or recovering from a cardiac event, and sometimes help evaluate the cardiac status before heart surgery.

### 7.2 Limitations of existing procedures

The process of stress echocardiography sometimes suffers from inter-observer variabilities. Current evaluation approach requires manually tracing the contour of the left ventricle in the echocardiographic images and calculating its end-systolic and end-diastolic volumes for further analysis. Assessments are carried out typically based on only one cycle of heartbeat, even if the standard guidelines recommend tracking and averaging five cycles before making a conclusion[165,166]. It potentially brings huge variance and uncertain accuracy to diagnostic results, especially for those less-trained medical assistants in low-resource regions.

Stress echocardiography also requires extremely sophisticated procedures to find an appropriate imaging location/orientation in a short time[40]. The acquired views must be of the same regions of the myocardium before and after exercise[40]. Same regions in the ultrasonographic window are required to make sure the images are from the same or a very similar

location/orientation of the heart. Otherwise, the measurements will be incorrect, because the chamber volume varies in images from different locations/orientations.

In addition, the end-point when the patient should stop exercising is dependent on whether patients achieve the target heart rate, experience moderate angina/severe chest pain, or reach tolerable exercise limits. These subjective criteria may result in suboptimal cardiac testing outcomes.

Furthermore, patients may be vulnerable to certain diseases that show up only during exercise, which is not monitored by existing procedures. For example, myocardial ischemia does not show in the resting state but appears only in stress, resulting in hypokinesia, akinesia, and dyskinesia. These symptoms are usually mild but can develop to be acute and lethal if neglected. However, because the patient is not monitored during exercise, the initiation of myocardial ischemia is unknown, not only missing valuable data but also leaving the patient at risk.

### 7.3 Echocardiography by the wearable imager

Those limitations of existing echocardiography can potentially be addressed with the wearable imager demonstrated in this study. The aperture of the wearable imager in this work is relatively small. The entire array length is 12.7 mm, which is shorter than the intercostal space (~14.5 to 19.7 mm[167]). Therefore, imaging is not challenged by the ribs because we only image from one position at a time, rather than using a big patch to cover many locations simultaneously. We imaged in the parasternal axis views and apical views at different locations (Extended Data Fig. 3). In the parasternal long axis view, the wearable imager was attached to the chest of the subject continuously during the entire process. The heart rate was estimated to be average 70 beats per minute for the rest stage, which changes occasionally though, but still within a normal range. Then, the subject exercised on a cycling bike while the device continuously monitored the heart status. As the exercise began, the heart rate gradually rose. The subject exercised as hard as possible to reach the possible highest heart rate. The whole exercise duration took around 12 mins. After exercise, the heart rate slowed down back to normal.

The LVIDs and LVIDd were measured during different stages. Before exercise, the average LVIDd and LVIDs were 45 mm and 27 mm, respectively. During exercise, growing need of blood supply accelerated the heart beats, but the LVIDd and LVIDs were slightly dropping because a shorter pumping period allowed fewer muscle extensions. At the peak of exercise, LVIDd roughly dropped to 38 mm and LVIDs went down to 22 mm. After exercise, the LVIDd and LVIDs recovered to approximately 41 mm and 24 mm. respectively. Fractional shortening, the reduction of end-diastolic diameter during systole, is a measure of the cardiac muscular contractility. It was calculated as the difference between the LVIDd and LVIDs divided by the LVIDd.

The parasternal long axis view contains information mostly about the left ventricle and atrium, while the apical four-chamber view provides a more comprehensive window of all four chambers and is more precise for estimating the left ventricular volume. Because the apical four-chamber view requires the patient to be tilted in the lying side position so ultrasound can enter from the apex, we could not collect these data during exercise. In the apical four-chamber view, we could see that both end-systolic volume and end-diastolic volume were increasing after exercise. This process of heart restoration is sometimes defined as heart volume reversal[168]. Reasonably, exercise leads to increases in both contractility and afterload of chambers, resulting in a physiological decrease in chamber volumes immediately.

Changes in ventricular size under stress may provide useful information regarding cardiac functions. For example, the end-systolic volume reversal may provide complementary information for risk stratification of cardiac diseases[168]. A decreasing end-diastolic volume may be a critical indicator of hypovolemia resulting from poor oral intake, emesis, or myocardial loss during cancer treatment[169]. An unsteady end-systolic volume recovery also portends the possibility of diseases like septal defects[170] or valvular regurgitation[171].

**Supplementary Discussion 8: Acoustic coupling of wearable imager**

In this study, the 50-micron layer of silicone underneath the device is in a liquidous state (Silbione part A, A-4717, Factor II Inc.). It was not used to encapsulate the array but instead as an acoustic couplant and adhesive layer. The silicone layer is a structural material that improves the acoustic coupling and adhesive properties of the device without limiting its flexibility.

Rigid probes cannot provide meaningful B-mode images without couplants due to incompatible mechanical properties between the rigid probe and the soft human skin (Supplementary Fig. 21d left panel). The stretchable ultrasound patch is packaged by skin-like soft polymers, i.e., SEBS in this case. The mechanical compliance of the patch allows intimate conforming to the human skin without external holding, which reduces interfacial air gaps and enhances acoustic coupling between the transducer and human skin. Therefore, the patch can image the human body even without coupling materials.

With that being said, microsized air bubbles may still exist at the wearable patch/skin interface. To achieve the best acoustic coupling, we added uncured Silbione (part A, A-4717, Factor II Inc.), a thin layer of liquid silicone whose acoustic impedance (1.03 MRayl)[172] is very close to the human skin, as the couplant underneath the device. The couplant can eliminate microsized air bubbles and enhance the coupling between the device and the skin. In addition, traditional hydrophilic ultrasonic gels are volatile and cannot last for long periods of time. The silicone couplant does not

evaporate at room temperature. Therefore, it can provide a good acoustic coupling over the long term.

To illustrate the performance of the silicone couplant, we compared the image quality of the wearable patch on the human body with and without the silicone layer (Supplementary Fig. 21d). The cardiac anatomic structures are clearly displayed. The image with silicone couplant is brighter than that without, indicating a stronger signal-to-noise ratio due to better interfacial acoustic coupling.

To quantify the coupling performance of the liquidous silicone, we have used a single transducer to sense a specific target. Specifically, we excited the transducer with a thin layer of liquidous silicone as couplant, and then acquired the reflected signals. For comparison, we also did the same test using commercial ultrasound gel as couplant. We did the experiments on a commercial phantom (Aquaflex, Parker Laboratories) with a reflector 18 mm underneath the surface. The results show a comparable signal-to-noise ratio between commercial ultrasonic gel and liquidous silicone (Supplementary Fig. 21), indicating the outstanding acoustic coupling performance of the silicone.

In addition, we also imaged the heart from four different views with commercial ultrasound gel as the couplant. The resulting images were compared with Supplementary Fig. 20 generated with liquidous silicone as the couplant. Images from all views show that the contrast between different structures and details in the cardiac anatomy are highly similar, and no obvious structural differences are found. These results show the practicality and excellent performance of liquidous silicone-based couplant.

The acoustic attenuation of the silicone is negligible. The attenuation depends on the ultrasound frequency and the material thickness. Given the attenuation coefficient of ~0.11 dB/mm for liquidous silicone at 3 MHz[173], a 50 μm thick couplant only causes 0.005 dB attenuation, which has practically no negative effect on the acoustic properties of the wearable ultrasonic array.

Furthermore, the liquidous silicone has a high viscosity (60 Pa·s)[174], which makes the device less susceptible to sliding on the skin, ensuring a consistent imaging window on the same target during long-term continuous monitoring.

**Supplementary Discussion 9: Continuous cardiac performance monitoring**

9.1 Monitoring ejection fraction, cardiac output, and stroke volume simultaneously

Commonly monitored vital signs contain body temperature, respiration rate, peripheral blood pressure, and heart rate. The first three parameters cannot directly reflect the heart status, while the heart rate only tells how quickly the heart is pumping but does not reveal the actual

performance of the heart. Other parameters, like ejection fraction, cardiac output, and stroke volume should be explored for a more comprehensive and conclusive diagnosis.

Ejection fraction represents the fraction of the blood ejected from the left ventricle per cycle. It is an indicator of the left ventricle's overall systolic performance (Equation 23). Cardiovascular diseases lurk in an abnormal fluctuation of ejection fraction. Normal left ventricular ejection fraction should be $\geq$50%, and a reduced one may manifest itself as heart failure[175]: a moderately reduced ejection fraction is within 40-49%, and a reduced one is <40%. Chronically attenuated ejection fraction is undoubtedly a danger sign, and an unreasonably high ejection fraction also entails troubles like hypertrophic cardiomyopathy[176], a common cause of sudden cardiac arrest. As one of the most clinically significant indices of cardiac function, ejection fraction is key in differentiating systolic versus diastolic heart failure, and is well correlated with mortality in stable outpatients with coronary artery disease and heart failure[177,178].

Cardiac output is a volumetric blood flow rate and is an indicator of the tissue oxygenation (Equation 24). The cardiac output, the product of the heart rate and the stroke volume, the blood volume pumped from the left ventricle every minute[179], can aid in the diagnosis of heart failure conditions, monitor patient status during surgeries and intensive care, and evaluate the overall cardiac functions[180]. The cardiac output is widely monitored in anesthesiology and emergency care[181]. Measurement of cardiac output is specifically essential in unstable patients whose condition may undergo dramatic changes in a short time, as it indicates an overall systemic oxygen delivery and tissue perfusion[182]. Many pathologies besides cardiovascular diseases lead to changes in cardiac output. Abnormally decreased cardiac output could be a sign of heart failure caused by valvular heart diseases[179], but also could be intoxications like acute azotemia[183], indicating a severe dysfunction of the kidney. Aberrant high cardiac output may be a complication of sepsis[184], hyperthyroidism[185], or anemia[186]. When evaluating the cardiac output, oftentimes a patient may appear asymptomatic during resting conditions due to a wide range of physiological regulatory processes such as vasodilation and minor increases of heart rate within the physiological range. Therefore, maximal cardiac output measurements during exercise are also of particular interest for their ability to reveal underlying problems in otherwise normally asymptomatic subjects.

Stroke volume is the difference between the EDV and the ESV, representing the absolute blood volume ejected from the left ventricle in a single cycle. Stroke volume, when used together with cardiac output and/or ejection fraction, can provide a much more comprehensive overview of the cardiac status. For example, as mentioned above, cardiac output can often appear asymptomatic. In those cases, the presence of a lowered stroke volume may reveal underlying heart failure. In other cases, a patient might appear asymptomatic if only considering the ejection fraction. Then, the presence of a decreased stroke volume would be able to indicate diastolic heart failure.

There are several other examples demonstrating why monitoring any one of these parameters in isolation may lead to inaccurate diagnosis results. For example, ejection fraction may overstate cardiac functions in left ventricular hypertrophy, which can lead to heart failure. In this case, ejection fraction remains normal, but stroke volume and cardiac output are dropping[187-189], which is also well known as heart failure with preserved ejection fraction. Diagnosis only based on ejection fraction would be wrong, while a comprehensive analysis on ejection fraction combined with cardiac output and stroke volume can generate correct results. Conversely, diastolic and systolic dysfunctions of the left chambers sharply reduce ejection fraction[190], but the cardiac output can maintain in the normal range with compensation of increased heart rate. Besides cardiac diseases, some surgical procedures may also have impacts on cardiac functions that require monitoring these three indices simultaneously.

This scenario is also paralleled in the measurement of blood pressure, which is commonly done in current practice. Blood pressure reflects a composition of multiple contributing factors, for example, cardiac pre-load and vascular resistance. Much like the scenarios presented by stroke volume, ejection fraction, and cardiac output, a normal blood pressure reading could obscure underlying abnormalities in the cardiac pre-load, and vascular resistance if measured in isolation. Measurement of the left ventricular volume can serve as an indicator for these factors contributing to blood pressure. The cardiac pre-load is how much the myocardium is stretched prior to contraction, and is reflected in the EDV[191]. Vascular resistance can be estimated from cardiac output using lumped parameter models of the circulatory system[192,193]. The ability to monitor the volume of the left ventricle can thus provide insight into the contributing factors to blood pressure and reveal diseases that might otherwise be obscured.

Therefore, ejection fraction, cardiac output, and stroke volume are important parameters for evaluating cardiac performance[194], and together provide a comprehensive analysis of the blood delivery capabilities of the heart. Continuously monitoring these indices of the heart for the long term is of strong prognostic value and has great potential to decrease the mortality and morbidity of many cardiovascular diseases and conditions.

### 9.2 Monitoring the left ventricular volume by 3D imaging

From Equations 22 to 24, the ejection fraction, cardiac output, and stroke volume are directly related to the volume of the left ventricle (EDV and ESV). Thus, the most direct method to measure these indices would be to monitor the left ventricular volume. Ideally, the most accurate and direct approach would be to capture 3D images of the left ventricular chamber throughout the cardiac cycle and use those 3D images to calculate the volume. Common methods of capturing these 3D images include traditional ultrasound, computed tomography, radionuclide imaging, and magnetic resonance imaging. However, these common cardiac imaging techniques have a host of limitations, including device bulkiness, low temporal resolutions, and long-term toxicity to the body.

Furthermore, in many cases, it is desirable to continuously monitor cardiac output in the operating theater to prevent complications during surgery such as shock[195], which is not viable for the aforementioned techniques given these drawbacks.

In addition, the calculation based on manual image labelling suffers from interobserver variability. Specifically, when labelling the left ventricular dimensions, the endocardial border requires to be traced continuously from one side of the mitral annulus to the other side. However, because the endocardial is hypoechoic, no sharp boundary can be easily seen in the image, yielding large discrepancies of left ventricular dimensions between clinicians' observations[42]. The discrepancies can be further magnified in calculating stroke volume, cardiac output, and ejection fraction, which results in an inaccurate diagnosis and introduces uncertainties in subsequent treatment regimens. In addition, less severe abnormality suffers from greater interobserver variability, where early symptoms will easily slip away if not well measured by two or more experienced echocardiographers.

### 9.3 Monitoring the left ventricular volume by 2D imaging

The next most direct method of obtaining the left ventricular volume would be to approximate the 3D volume of the left ventricle through 2D imaging. This approach faces similar problems due to the use of imaging but is computationally faster and can achieve higher temporal resolutions than 3D imaging.

While 2D imaging with ultrasound is a widely used approach for its convenience, traditional ultrasound has a bulky housing and requires the probe to be manually held in place. Point-of-care ultrasound probes transcend the limitations of traditional medical imaging[11] and promote precision medicine for household use, but they still require an external force to maintain a stable coupling with the skin. Otherwise, the image window will change and generate unfair comparisons, which results in misdiagnoses. In addition, their use highly depends on the clinicians' experience, which is prone to generating inter-observer variabilities and operational errors[196]. Robotic arms have been applied in such a case, but the higher cost and the even more bulky design are the new problems[197], making it inaccessible in most cases and impractical for continuous and long-term measurements.

### 9.4 Monitoring the left ventricular volume by model estimations

Therefore, the traditional approaches to continuously monitoring stroke volume, cardiac output, and ejection fraction do not tend to employ imaging, but instead use models to estimate these parameters from other indirect measurements. Here, we provide a review of some of the major relevant non-imaging methods.

(a) Fick's method

According to the Fick principle, the ratio between the rate at which oxygen is absorbed into the blood and the rate at which blood is delivered through the body, is directly represented by the difference in blood oxygen content between mixed venous blood and arterial blood. This principle is summarized by[180]:

$$CO = \frac{VO_2}{C_a - C_v} \quad (25)$$

where $CO$ is cardiac output, $VO_2$ is the amount of pure gaseous oxygen consumed per unit time, and $C_a$ and $C_v$ are oxygen content of arterial and mixed venous blood, respectively. Typically, $VO_2$ is measured using a spirometer, while $C_a$ and $C_v$ require catheters to be inserted into the patient. $C_v$ is measured from the pulmonary artery or vena cava, while $C_a$ is often measured from the brachial or femoral artery[180].

While Fick's method can be highly accurate, in the range of 5 to 10%[180], and is often used as a benchmark for other methods, it is highly invasive and requires catheterization. Furthermore, to take accurate readings, the patient's cardiac output and oxygen consumption must be stable for several minutes at a time[180]. It is also inconvenient for infants, or during surgery, because a sufficient blood volume is required for this technique[180].

(b) Indicator dilution techniques

Indicator dilution is a variation of the Fick principle and works in a similar way. Rather than using oxygen as an indicator, other indicators can be injected into the bloodstream at a single point, and their concentration is analyzed downstream. The most common indicators used in dilution are indocyanine green dye (dye dilution), or cold saline (thermodilution).

In the case of dye dilution, the dye concentration is measured using a densitometer, based on the optical density of the blood. This is then plotted over time, and the area under the curve can be related to the cardiac output and the amount of dye initially injected using the Stewart-Hamilton equation[198]:

$$CO = \frac{V_{indicator}}{\int_0^\infty C(t)dt} \quad (26)$$

where the numerator is the initial amount of indicator injected, and $C(t)$ is the concentration of the indicator measured downstream over time.

In reality, because the dye is not removed from the bloodstream by the kidneys sufficiently fast, the concentration curve peaks multiple times as the dye recirculates[180]. Therefore, the curve must be extrapolated so that it returns to zero concentration.

For thermodilution, the Stewart-Hamilton equation is modified to account for the heat transfer between blood and saline[180,198]:

$$CO = \frac{K * V_{indicator} * (T_{blood} - T_{indicator})}{\int_0^{\infty} T_{measured}(t)dt} \quad (27)$$

$$K = \frac{c_{indicator}\rho_{indicator}}{c_{blood}\rho_{blood}} \quad (28)$$

where $c$ is the specific heat, $\rho$ is the density, $V_{indicator}$ is the volume of indicator initially injected, $T_{blood}$ and $T_{indicator}$ are the initial temperatures of the blood and indicator, respectively, and $T_{measured}(t)$ is the temperature measured downstream over time.

The injected saline lowers the temperature of the blood as it travels through the bloodstream, followed by warming as it is mixed and diluted in the blood. This is measured using a thermistor.

Indicator dilution is unsuitable for continuous monitoring as the temporal resolution is too low. One must wait for roughly 1 minute for the indicator to circulate and fully dilute[180]. In addition, the densitometer requires a lengthy calibration process[180]. Unlike the Fick method, there is no self-sustaining constant flux of indicator, so periodic reinjection is required. Over time, this can increase the risk of embolism, infection, and fluid imbalances[180].

These methods can also be error-prone. For example, thermodilution techniques will have limited accuracy given unstable body temperature, but this may be uncontrollable in certain cases, like liver transplantation surgery[199]. In addition, arterial pulse waves could also be transferred to cardiac measurements, but this is also unreliable for monitoring on trends because it cannot compensate for circulatory changes. Also, the Stewart-Hamilton model itself assumes ideal fluid flow. Many of the assumptions of ideal fluid flow are violated by the human circulatory system, such as single inflow and single outflow tracts, complete mixing of the fluids, steady flow, and no recirculation of indicator[198].

Additionally, in dye dilution, the downslope of the concentration curve must be extrapolated extensively due to recirculation of the dye, limiting the accuracy. In thermodilution, one must carefully maintain the temperature of the injectate, or else it may cause the temperatures to deviate. Thermodilution is also unreliable in low cardiac output situations, where it tends to overestimate

the cardiac output. Under low-flow conditions, the indicator has more time to equilibrate with the surrounding tissues, leading to diminished measured temperature changes and a smaller area under the curve[200], because heat exchange is assumed to only occur between blood and saline.

(c) Conductance catheterization

In conductance catheter measurements, a catheter with multiple electrodes segmented along its length is inserted into the left ventricular chamber. The blood within the chamber is segmented into different volumes stacked together, with their boundaries defined by the left ventricular wall and the equipotential surfaces through the electrodes[201]. Using the dielectric and conductive properties of the blood, each separate volume of blood can then be treated as a resistor and capacitor in parallel[201]. The height is the distance between electrodes. The cross-sectional area is the cross-sectional area of the blood inside the left ventricle[201]. This cross-section varies over time throughout the cardiac cycle. The conductance, 1/R, can be modeled by[201]:

$$\frac{1}{R(t)} = \frac{\sigma V(t)}{L^2} \quad (29)$$

where $\sigma$ is the conductance of the blood, $V$ is the volume of the segment, and L is the length between electrodes. From there, the volumetric contribution of each segment to the stroke volume can be found[201]:

$$\Delta V = \frac{L^2}{\sigma}\left(\frac{1}{R_{be}} - \frac{1}{R_{ee}}\right) \quad (30)$$

where $R_{be}$ and $R_{ee}$ are the resistances at the beginning and end of ejection, respectively.

The summation of each segment then gives the total stroke volume, and from there, ejection fraction and cardiac output may also be determined.

This method has the disadvantage of being highly invasive, requiring catheterization of the left ventricle. The conductivity of blood must also be calibrated. This can lead to errors, as the conductivity of blood changes throughout the cardiac cycle (see the Bioimpedance section). The model also assumes the equipotential surfaces to be parallel and the blood in the ventricle to be the only conducting objects, which may deviate from the real situation[201].

(d) Radionuclide angiography

Traditionally, gamma cameras used in radionuclide angiography were too slow to perform continuous monitoring of left ventricular volume, ejection fraction, and cardiac output. However, now there are small and lightweight scintillation probes, which can perform radionuclide

angiography right at the bedside. A radionuclide indicator is injected into the circulation, and the radionuclide count density is measured throughout the cardiac cycle[202,203]. The maximum count density throughout the cycle represents end-diastole, while the minimum represents end-systole[202]. In this way, the ejection fraction may be determined with the following equation[202,203]:

$$EF = \frac{end\ diastolic\ counts - end\ systolic\ counts}{total\ diastolic\ counts - background\ counts} \quad (31)$$

Count averaging is used (roughly 5 heartbeats) to increase the reliability of the measurement and raise the count density[202]. The drawback of this approach is namely the ionizing radiation, which is safe in shorter timeframes, but unsuitable for extended monitoring. Because count averaging is used, irregular activities such as fibrillation or ectopic activities can affect the accuracy[202].

(e) Velocity measurements

Cardiac output can also be found by measuring the blood velocity at a vessel, while knowing its diameter. Ways of measuring the blood velocity include Doppler ultrasound and electromagnetic flow probes. In Doppler ultrasound, the changes in the signal frequency are related to the blood flow velocity[26]:

$$\frac{Fd}{Fs} = \frac{u}{x} \quad (32)$$

where $Fd$ is the frequency shift, $Fs$ is the source frequency, $u$ is the velocity, and $x$ is the sound speed. Thus, by measuring the frequency shift, the flow velocity can be measured.

In electromagnetic flow probing, a probe is implanted and slipped around a blood vessel. According to Faraday's law of magnetic induction, a conductor (i.e., the blood in this case) moving through a magnetic field generates a voltage that is proportional to the velocity[180]:

$$e = BLu \quad (33)$$

where $e$ is the induced voltage, $B$ is the magnetic flux density, $L$ is the spacing between electrodes, and $u$ is the flow velocity.

Velocity measurements must first be calibrated by measuring the blood vessel diameter before it can be converted to cardiac output[192]. In the case of Doppler, the angle of the probe to the blood flow must additionally be identified[180]. While studies have found Doppler to be inaccurate in measuring cardiac output, especially in children, its strength is in monitoring the trends and its capability of detecting rapid changes[192]. This makes it useful for monitoring surgical or fluid

administration procedures in clinical settings. In addition, Doppler is one of the few non-invasive methods that measure cardiac output.

Electromagnetic flow probes face significant drawbacks in that they require the blood vessel to be fully exposed through surgery such that the probe can be positioned around the blood vessel[180]. The accuracy of this method also depends strongly on how well the surgical exposure is done[180]. Overall, it is an unfavorable method in most situations.

(f) Pulse contour analysis

In pulse contour analysis, the arterial pulse wave is recorded to produce a measurement of cardiac output. The area under the pulse wave curve is related to the stroke volume, and therefore the area under the curve times the heart rate can be related to the cardiac output[192]. To derive the relationship for reliable monitoring, a three-element Windkessel model of the aorta's mechanical characteristics and the peripheral resistance of the body was used to represent the circulation[192,193]. To calibrate the model, the relationship between the aortic pressure and cross-sectional area must be derived, so that the vessel compliance can be estimated[193]. Studies have also further developed the model to be able to identify the stressed and unstressed states of the left ventricle and additionally calculate the ejection fraction[204].

Due to the significant amount of calibrations required for this approach, the accuracy is limited. In addition, as with most models, ideal conditions must be satisfied for the model to work well. Indeed, current commercial devices for measuring cardiac output via pulse contour analysis demonstrate a lack of ability to properly account for peripheral circulatory changes[192]. The pulse contour analysis method can be unreliable during surgeries, such as during liver transplantation, where cirrhosis patients have high cardiac output and low fluctuating peripheral resistance[192].

(g) Bioimpedance

Bioimpedance techniques relate the changes of electrical impedance across the thorax to the cardiac cycle to monitor the cardiac performance. Bioimpedance is also one of the few non-invasive techniques for monitoring cardiac output. Classical methods of processing the impedance signals were dubious in their accuracy and whether they measured the blood flow of the heart at all[192]. Patients with conditions such as pulmonary edema, where excessive lung water was present, repeatedly had poor results in validation studies of bioimpedance[192].

Recently, more reliable methods of processing the bioimpedance signals have been developed. Electrical velocimetry is based on the idea that red blood cells are randomly oriented when there is no blood flow in the aorta, and subsequently become aligned when the aortic valve opens. This alignment produces a change in the conductivity of the blood.

The overall bioimpedance measured can be modeled with the following equation:

$$Z(t) = Z_0 + \Delta Z_R + \Delta Z_C \quad (34)$$

where $Z_0$ is the quasi-static background impedance, $\Delta Z_R$ is the artifact from respiration, and $\Delta Z_C$ is the impedance change due to the cardiac cycle.

From the impedance curve of $\Delta Z_C$, one can determine the acceleration of the blood flow based on the time taken to align the red blood cells. An average velocity can then be estimated and converted to stroke volume and cardiac output based on body mass.

In adults, electrical velocimetry is just within the 30% limits of agreement[205], but is not reliable in children, likely due to the body mass-based assumptions[192].

In summary, these methods tend to have several sources of error due to their reliance on models and assumptions. The lack of imaging-derived left ventricular volume measurements causes them to be reliable only under ideal conditions and to include extraneous signal sources in their measurements. The use of imaging is not viable in long-term continuous monitoring either, with the currently available devices. Non-imaging techniques can monitor for slightly longer periods of time, but are generally too invasive and inconvenient, with many requiring catheterizations and injections, cannot be used in everyday life or during exercise, and are also not viable solutions for long-term monitoring. In addition, those invasive approaches require expertise in the placement and manipulation of the catheters to get the correct results. Only well-trained clinicians can handle this measurement modality[97]. As a result, many of these techniques are typically only used within the intensive care unit or during an operation, where their benefits sufficiently outweigh the drawbacks. Thus, there is a significant technological gap that can be filled by a wearable, versatile, and non-invasive imager that can do direct imaging-derived measurements of the left ventricular volume.

### 9.5 Anatomical considerations of imaging posture

In the apical four-chamber view, we could only collect data during recovery but not during exercise (Fig. 5). This is not to do with a technological limitation, but rather an intrinsic anatomical fact. Extended Data Fig. 4 has shown that the apical four-chamber view is best captured in the lying side position, which has limited the continuous monitoring during exercise. From our supplementary data (Supplementary Fig. 28) and literatures[206,207], it is generally accepted that the apical four-chamber view is best captured in the lying side position and will be partly blurred/blocked in other positions. The lying side position is the clinically required position to obtain a qualified apical four-chamber view[206,207].

The positional requirements for the apical four-chamber view are very strict for two reasons. First, only when the patient is in the lying side position does the ultrasound beam enter the heart's apex at a straight angle (Supplementary Fig. 28). Other positions (e.g., standing) will yield incorrect measurements of chamber dimensions and thus cardiac indices.

Second, we compared the apical four-chamber images from a commercial imager taken by an experienced cardiologist and from a wearable imager taken by a trained amateur, both with the subject in the standing position (Supplementary Fig. 28). The cardiac chamber morphologies cannot be fully reconstructed in either image. The right ventricle and right atrium have been totally shadowed by the lungs. While we can roughly see the morphology of the left ventricle and left atrium, the image quality is poor. The left ventricular endocardium cannot be visible, and the left atrium is malformed, because part of the ultrasound array has been blocked by the lungs and only a small fraction of the elements play an effective role in the imaging process[207] (Supplementary Fig. 28). It results in images with low spatial-resolutions and low contrast-to-noise ratios.

On the contrary, lying in the lying side position brings the relative orientations of the heart and the transducer array out of the lung shadow. Therefore, the inability to collect high-quality and morphologically representative apical four-chamber images in the standing position is more of an intrinsic anatomical fact rather than a limitation of the commercial imager or the wearable imager. Thus, we imaged the heart in the apical four-chamber view only with the lying side position.

However, the apical four-chamber view is not the only source of information in echocardiography. We have also presented continuous monitoring of the parasternal long axis view throughout the whole process (before exercise, during exercise, and during recovery; Fig. 4), as the anatomy does not limit this view as strictly as it does for the apical four-chamber view. The M-mode recording in Figure 4b is extracted from the B-mode recording. Extended Data Fig. 5 shows that the patch is capable of taking B-mode images during exercise and illustrates how the M-mode is extracted. The chamber motion is noticeable, and the change of heart rate is also computed in Figure 4c. Abundant information, such as left ventricular internal diameter end diastole/systole, the fractional shortening ratio, and the anatomic integrity of the left ventricle are also determined. In addition, Figure 4d and Figure 4e are presenting the details from different stages, demonstrating the capability of wearable imager in the continuous monitoring during exercise.

In addition, monitoring the cardiac status can be valuable during many surgeries and other interventions to prevent further complications[208]. Given that the lying side position is the standard position during the echocardiography measurement for ejection fraction, our device can be directly applied to numerous situations where monitoring of cardiac function is essential and lying side position is easy to reach. For example, during general anesthesia as well as the postoperative period,

there is always the risk of cardiac events for patients who undergo noncardiac surgeries, such as myocardial infarction and heart failure[208-212]. Therefore, the monitoring of the cardiac function is valuable in this context especially for the patients with risk factors[208]. During the beginning of general anesthesia, the lying side position can be easily reached, thus providing a preliminary evaluation of the cardiac function preoperatively with our device.

In this work, two-dimensional cardiac images can generate an approximate left ventricular volume by the Modified Simpson's rule, which is widely accepted in clinical diagnosis even though it comes with approximation and inaccuracy. Future work can follow up on developing solutions for imaging in the parasternal long axis view during exercise/stress testing. For example, we can fabricate a 2D matrix probe to reconstruct images three-dimensionally. The cardiac volume and other related parameters can be directly and more accurately computed from three-dimensionally reconstructed images.

**Supplementary Discussion 10: Detailed left ventricle working processes**

A new cardiac cycle starts from the end of left ventricular relaxation in the last cycle. When the pressure in the left ventricle drops below that in the left atrium, the pressure difference pushes the mitral valve to open, so blood flows from the left atrium to the left ventricle. The inflow of blood triggers the first volume increment of the left ventricle, which is called rapid inflow. The blood inflow builds up the pressure in the left ventricle and at the same time releases the pressure in the left atrium. As the pressure difference decreases and the inflow rate slows down, the volume of the left ventricle hits the first equilibrium, and the mitral valve tends to close. This period is the interval between passive blood flow and active blood flow caused by atrium contraction, which is named diastasis. Afterward, the left atrium starts to contract to build the second pressure difference. The pressure difference again enforces the mitral valve to open and causes the second stage of blood inflow to the left ventricle. Simply, the process of this pressure generation is called atrial systole or atrial contraction. At the end of this process, the volume of the left ventricle reaches its peak as the second equilibrium.

Then, the left ventricle starts to contract to raise the pressure inside. Because the mitral valve and aortic valve keep closed during the contraction, the volume of the left ventricle holds. Therefore, this contraction is called isovolumetric contraction. The mitral valve is a one-way valve, so there is no blood countercurrent back into the left atrium and only the aortic valve opens after the isovolumetric contraction. The blood in the left ventricle is then ejected into the aorta, which is called rapid ejection. The rapid ejection results in an obvious drop in the left ventricular volume and pressure so there is a sharp slope in the curve. Like the rapid inflow, as the pressure difference between the aorta and left ventricle vanishes, the aortic valve closes. Following the ejection, a small portion of blood in the left ventricle remains. Because the aortic valve is also a one-way

valve to keep the unidirectional blood flow, the left ventricular volume keeps stable during relaxation, which is also named isovolumetric relaxation. At the end of the isovolumetric relaxation, next cardiac cycle commences.

The precise monitoring of cardiac events can provide more insights into pathologies of cardiac dysfunctions. For example, the ejection rate based on the volume curve can reveal the myocardium contractility and stenosis of aortic valves.

**Supplementary Discussion 11: Neural network for continuous ultrasound imaging**

11.1 Developing the deep learning model

We surveyed different deep learning models in the literature and made the selection based on their popularity and relevance to this study. Currently, widely-adopted image segmentation deep learning models can be classified into two categories: U-Net models, which are known for fast and precise biomedical image segmentation with multiple variants[213], and Fully Convolutional Networks (FCN) models, an end-to-end deep learning method, which first uses a series of fully connected convolutional layers to find useful features from the input image and then use an upsampling layer to restore the output image size to the same as the input image size[214].

Specifically, U-Net models are based on the well-established U-Net architecture for fast and precise biomedical image segmentation[213]. U-Net architecture[213] has gained wide adoption. Many researchers have proposed variant models based on the U-Net architecture. In this study, in addition to the original U-Net model, we also worked with its variants, including Attention-U-Net (U-Net model with an attention gate)[215], U-Net++ (U-Net with redesigned skip pathways, dense skip connections, and deep supervision)[216], NAS-UNet (Neural Architecture search with a U-Net architecture)[217], U-Net+ResNet50 (U-Net with ResNet encoders)[218], and U-Net mini (a simplified structure of the original U-Net[219]).

The FCN model is widely used for semantic segmentation[214]. FCN gained wide adoption for its outstanding performance and no restriction on the input image size. Specifically, an FCN model first uses a series of fully connected convolutional layers to find useful features from the input image and then uses an upsampling (backwards strided convolution) layer to restore the output image size to the same as the input image size. If we return the output directly after solely using the upsampling layer, we call the network FCN-32 based on the number of upsampling operations. It is believed that spatial location information could be lost when going deep in the neural network. One solution for this is to fuse the predictions of multiple stages of the convolution layers. FCN-8, for example, fuses the predictions of the final three layers, and therefore only needs to upsample for eight times. Unless otherwise specified, all of our models used vanilla encoder as auto-encoder.

We also experimented with other types of auto-encoders, such as VGG and ResNet, in which cases we specify the auto-encoder names in the model name.

To ensure accuracy of the pre-processing of input images, we performed frame-level segmentation/labelling of the left ventricle under the guidance of a cardiologist, and followed the Modified Simpson's rule[220] for volume calculation. For the commercial imager, we collected totally 500 frames on static subjects, 221 out of which are manually labelled for training. For the wearable imager, we collected overall 500 frames on the static subjects and 5858 frames on the moving subjects, 201 and 2029 out of which, respectively, are manually labelled for training.

We used data augmentation techniques to expand the limited labelled dataset. We applied rotation, scaling, and gaussian noise to the data to augment the dataset. Specifically, we generated four additional images for each of the labelled images by rotating the original data clockwise/counterclockwise by 5 degrees, rescaling it by a factor of 1.2, and applying gaussian noise to it. The results indicated that the data augmentation slightly improved the performance of the deep learning model (Supplementary Fig. 27).

We evaluated the models' performance quantitatively using Mean-Intersection-Over-Union (mIoU), which is one of the most widely used evaluation metrics for benchmarking object detection[221]. Specifically, intersection-over-union (IoU) is used to compare the similarity between two shapes[221] and is calculated using:

$$IoU = \frac{Area\ of\ intersection}{Area\ of\ union} \quad (35)$$

The mIoU is then calculated by taking the mean of IoU for all images in a dataset. In this study, we calculated mIoU by taking the mean of IoU of each pair of the deep learning model predicted image segmentations and the manually labelled image segmentations. Specifically, for each "(predicted-image, labelled-image)" pair, its IoU equals to the number of pixels within the left ventricle in both images divided by the total number of unique pixels within the left ventricle by either of the two images. The evaluation results for different models are listed in Supplementary Fig. 26 and Supplementary Table 5.

During exercise, small amounts of the recorded B-mode images were corrupted when the heart was blocked by the deep-breathing lung. To solve this problem, we applied an imputation algorithm to complement the missing parts in these corrupted images to generate a continuous waveform (Supplementary Fig. 29).

## 11.2 Detailed analysis of the neural network

Wearable sensors can generate continuous images. A milestone in the roadmap of developing wearable sensors is to integrate deep-learning algorithms to process the continuous images automatically. There are some commercial systems[91] and research studies[3,90] on developing algorithms or deep-learning models for quick calculation of ejection fraction and cardiac output, but the data output is still discontinuous because the commercial probe needs to be operated manually and can only produce intermittent data points. Integrating the wearable patch with deep-learning models can continuously output a large amount of actionable information and clinical insights, which have not been achieved in the literature.

For the deep learning component, our primary goals are (1) to identify image segmentation models to automatically extract the left ventricular volume from B-mode images; (2) to integrate the best-performing models in our research settings and analyze their performance. In the following sections, we provide more analysis and discussions about training paradigm comparison and model selection, data collection and processing (containing sample sizes), model implementation and parameter tuning strategy (containing learning rate), model performance, and future directions.

(1) Training paradigm comparison and model selection

Researchers have made tremendous progresses in improving automatic image segmentation using various types of machine learning models in recent years. Most researchers focused on using supervised learning models[222,223], which are models that learn from a training dataset with “correct” ground-truth labels. Many researchers have reported promising results using supervised learning models for medical image segmentation[3,223]. Although supervised learning models achieve high accuracy in image segmentation tasks, their weakness is in the expensive training cost because they acquire the training set through manual labeling in most cases.

In this study, we mainly focused on integrating supervised deep learning models into our research settings because segmentation results with a high accuracy are a prerequisite for extracting accurate cardiac performance metrics from the images. The weakness of supervised learning can be considerably mitigated by few-shot learning and reinforcement learning (detailed discussions are provided in future directions below). Among various supervised learning image segmentation models, those gaining the most popularity[224] are Convolutional Neural Networks, which are deep learning models that directly learn from raw pixel data by adaptively assigning weights to different areas/pixels of the input[225]. Specifically, in the field of medical image segmentation, fully Convolutional Neural Networks have achieved superior results compared to traditional Convolutional Neural Networks models in many studies[213]. Therefore, we chose to work with different variations of FCN models and U-Net models that are fully Convolutional Neural Networks performing particularly well within medical research settings[222,223,226]. We have

discussed each of the specific variations of these models in the Methods and Supplemental Information.

(2) Data collection and processing

The deep learning models in this work were implemented with readable, consistent, and well-structured codes, high-quality datasets, and reproducible results. The sample size is listed in Supplementary Table 4. All data were used by all models. Notably, we worked with two types of datasets in this study. The first type was "static" datasets, which contained left ventricle ultrasound B-mode images we collected from static subjects. The second type was "after exercise" datasets, which contained left ventricle ultrasound B-mode images we collected from subjects after intense physical exercise. The "after exercise" datasets were more challenging to process because (1) the size of the left ventricle was continuously and rapidly changing, leading to a higher variability in the dataset; (2) it contained noisier images, in which the left ventricle was sometimes blocked by the lung because the subjects were breathing heavily. To the best of our knowledge, this study represents the first to continuously segment cardiac ultrasound images with intensively changing left ventricular volumes. After collecting the data, we cropped the image size to be 512 × 512 pixels and manually segmented them to create the ground-truth as the training dataset. Furthermore, we used data augmentation techniques such as rotating, scaling, and adding gaussian noises to expand the size of the training dataset.

(3) Model implementation and parameter tuning strategy

We implemented the models based on open-source codes listed in Supplementary Table 3. The final hyperparameters after careful tuning in this study are also listed in Supplementary Table 3. Specifically, when training the models, we selected adaptive moment estimation (Adam) as the optimizer for all models. We found Adam to be the best optimizer among other optimization algorithms because it not only trains the best-performing models but also converges faster and requires less time for tuning. As an adaptive optimizer, Adam adaptively changes learning rates for different parameters, and, therefore, the models' performance was not much affected by the initial learning rate. Thus, we applied each model implementation's default learning rate in most cases.

Because most of the neural network models in this study were very deep, batch normalization was applied to make the networks faster and more stable. Specifically, when training deep neural networks, an update in the training process could change parameters in all layers, causing them to have different distributions after each update. This makes the optimization process like chasing a "moving" target and thus causes the network to become unstable and slow to train. We applied batch normalization to solve this problem. Specifically, each Convolutional Neural Network layer was connected with a batch normalization layer. The batch normalization layers standardized the network layer's output by applying a transformation that makes each layer's output mean close to

0 and standard deviation close to 1. This will effectively keep each layer's input distribution unchanged, and thus stabilize the network and speed up the optimization process.

(4) Model performance

We have compared the models' performance both quantitively and qualitatively (Extended Data Figs. 6 and 7, and Supplementary Fig. 26). Specifically, we found that images segmented by FCN-32 model achieved not only the highest mIoU but also the highest image fidelity. We achieved automatic image segmentation using the FCN-32 model.

One of the reasons that FCN-32 achieved better results than U-Net models was that FCN-32 did not have skip-connections. As introduced in the Supplementary Fig. 25, FCN-32's structure included a series of fully-connected convolutional layers and a single upsampling layer at the end. Compared with FCN models, U-Net models had more upsampling layers and concatenated outputs of previous layers using skip connections. Although these differences made U-Net models more robust to scale variations, there might be risks that the connections brought back some unnecessary features. Because all of our data were of similar scales, the robustness to scale variations was not a priority in our dataset. Therefore, the additional robustness brought by the skip-connections in U-Net models was unable to make significant improvements to the model. On the other side, the skip-connections and additional up-sampling layers possibly brought back redundant features that harmed the model's performance. We found FCN models usually output one singular region of left ventricle, while U-Net models sometimes output a few small detached external pieces (Extended Data Fig. 6). Additionally, the edges of U-Net's segmentations also appeared to be "fuzzier" than those of FCN's, proving that FCN was better at removing redundant features (Extended Data Fig. 6). Similarly, although the fusing step in FCN-8 could restore some location information, it could also bring back redundant information that was already filtered by previous layers, and hence made FCN-8's performance slightly worse than FCN-32's.

(5) Future directions

The FCN-32 model with the current dataset was sufficient for image segmentation in this study, which facilitated the frame-by-frame output of those cardiac indices. The FCN-32 had good generalization capability regarding different left ventricular volumes and different image noise levels. As for the application to new transducers and perspective data on new subjects in future studies, the generalizability of the current model could be further improved by expanding the training dataset or optimizing the network. For example, few-shot learning[49], an advanced version of supervised learning, can achieve promising results with the model trained on only a few labeled images for each subject[227]. Using this model, it is easier to expand the cohort size to achieve a higher data diversity by collecting images from a small number of subjects. Another potential strategy is reinforcement learning[50], which is another training paradigm alongside supervised learning and unsupervised learning. It can optimize models' training and data augmentation

approaches so that those models can be better tuned and less overfitted[226], thus improving the models' generalizability. By integrating those strategies, the FCN-32 model will potentially be adapted to a larger population with nosier data.

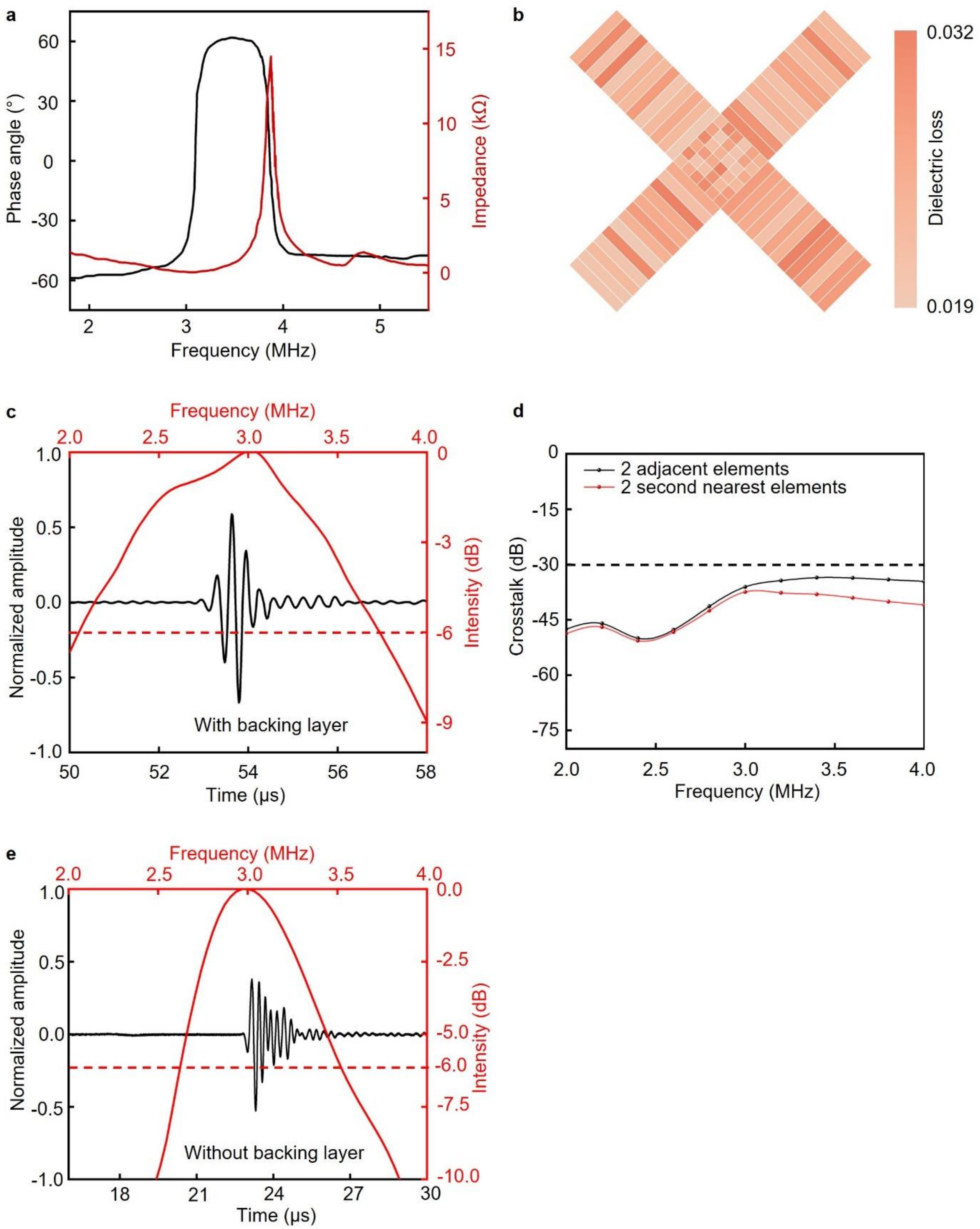


**Supplementary Fig. 1 | Characterization of the transducer array. a**, Electrical impedance spectrum with the amplitude and phase angle. It shows the 3 MHz resonance frequency and 3.87 MHz antiresonant frequency. The calculated electromechanical coupling coefficient is 0.67. **b**, Map of the dielectric loss of all transducer elements in the orthogonal imager. **c**, The pulse-echo

response and corresponding frequency spectrum of the transducers, showing a wide bandwidth of ~55% and a central frequency of 3 MHz. **d**, Crosstalk between a pair of adjacent elements and a pair of second nearest neighbors, which is lower than the standard -30 dB indicated by the dashed line. **e**, The pulse-echo response and corresponding frequency spectrum of the transducers, showing a wide bandwidth of ~30% and a central frequency of 3 MHz.

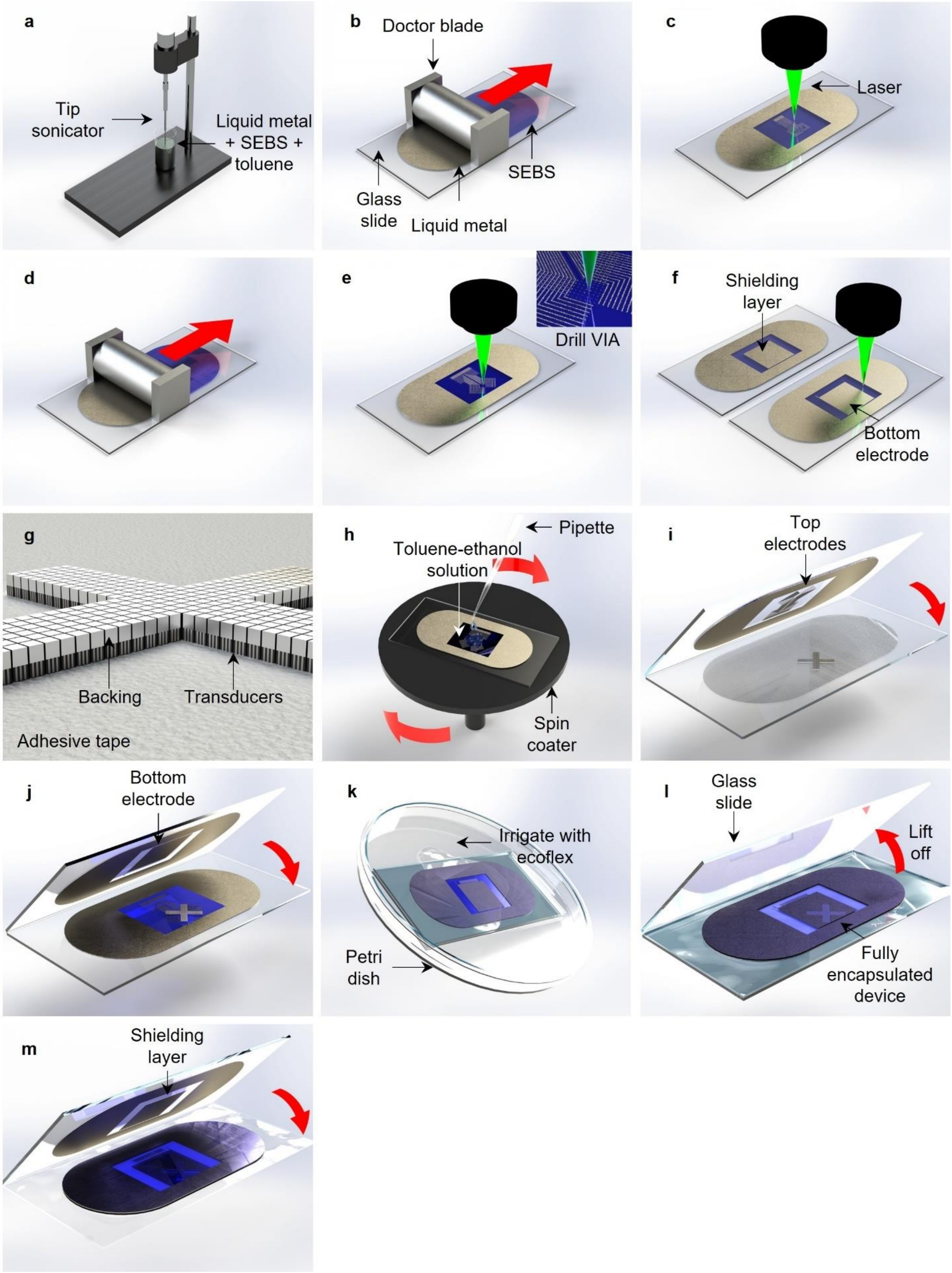


1798 **Supplementary Fig. 2 | Fabrication processes of the wearable imager. a**, Sonicate liquid metal

in toluene with SEBS to homogenize the materials. **b**, Print the liquid metal composite on a SEBS substrate using a doctor blade. **c**, Pattern the liquid metal-based composite electrode using laser ablation. **d**, Print a subsequent layer of liquid metal insulated with a layer of SEBS on the previous electrode. More layers of electrodes can be fabricated by repeating this step. **e**, Drill vertical-interconnect-accesses (VIAs) using laser ablation to allow electrical connection between the top electrodes and transducers. **f**, Pattern the shielding layer, bottom electrode, and alignment mask using laser ablation. **g**, Dice the transducer array together with the backing layer. **h**, Spin coat toluene-ethanol solution onto the electrodes to allow adhesion between electrodes and transducers. **i**, Bond the top electrodes to the transducer array. **j**, Bond the bottom electrode to the transducer array. **k**, Irrigate the gap between the two glass slides with Ecoflex to encapsulate the device. **l**, Lift off the glass slides to release the device. **m**, Soften the shielding layer and bond it to the device.

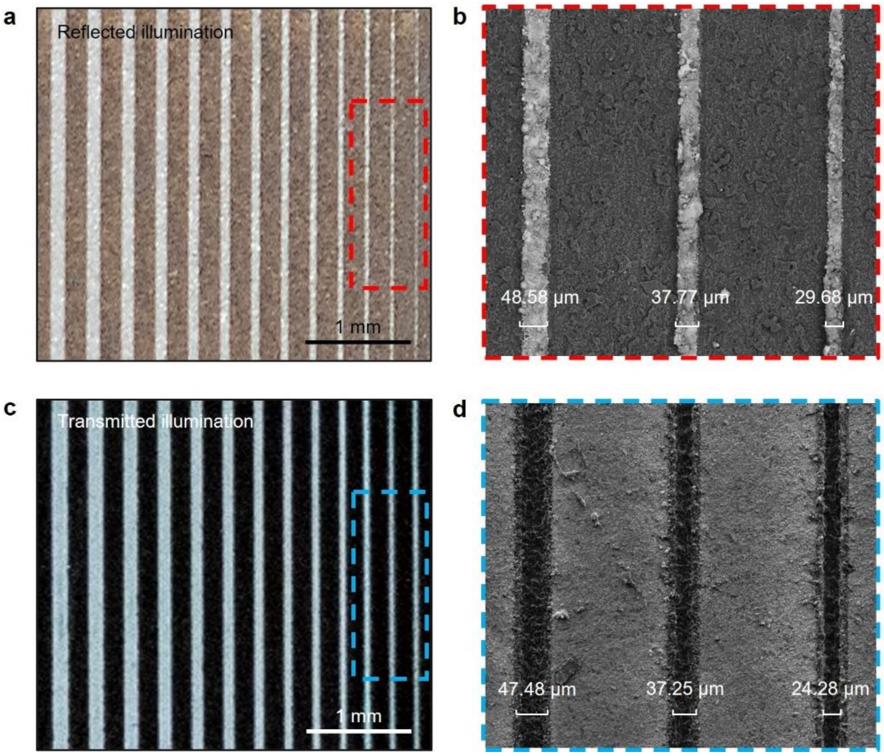


**Supplementary Fig. 3 | Images showing the fabrication resolution of the liquid metal composite electrodes. a**, Optical image with reflected illumination and **b,** scanning electron microscope, showing the minimum width of the liquid metal composite electrodes. **c**, Optical image with transmitted illumination and **d**, scanning electron microscope, showing the narrowest grooves on the liquid metal composite electrodes patterned by laser ablation.

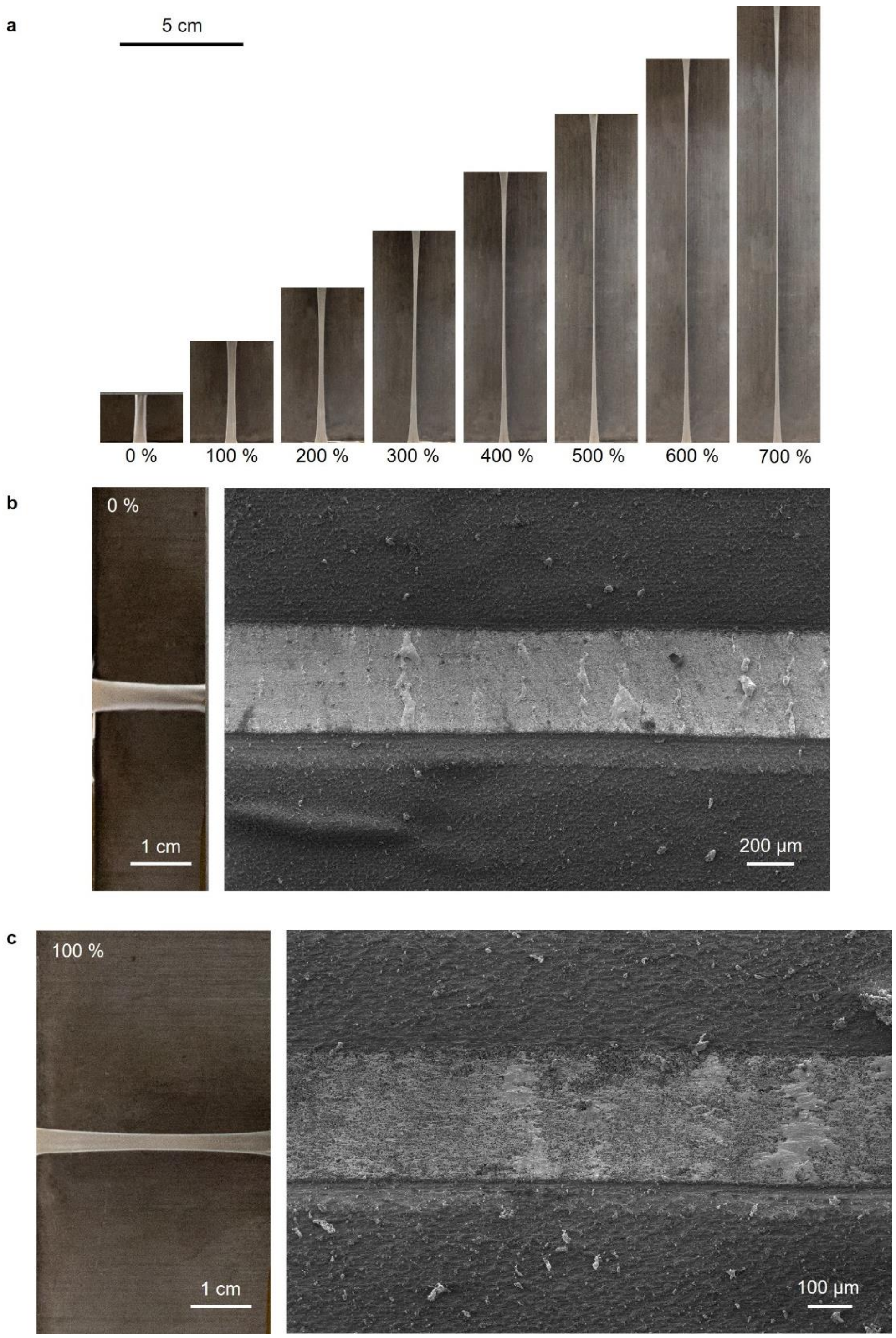


**Supplementary Fig. 4 | Mechanical testing of the liquid metal composite electrodes. a**, The high stretchability of the electrodes allows ~700% maximum strain. Optical and scanning electron microscope images of the liquid metal composite electrodes **b**, before and **c**, after uniaxially stretched for 100 % strain. There are no visible cracks in the electrode after stretching, indicating its excellent intrinsic stretchability.

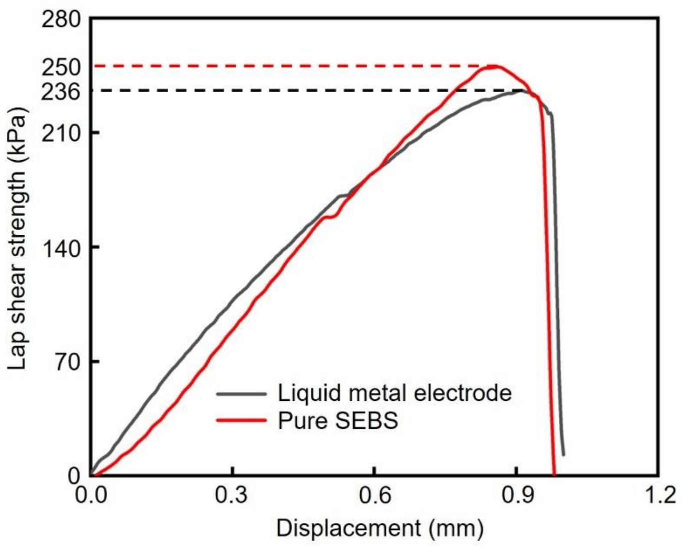


1821 **Supplementary Fig. 5 | Results of lap shear strength tests.** Both liquid metal electrode and pure
1822 SEBS are characterized. The curves peak values represent the bonding strength.

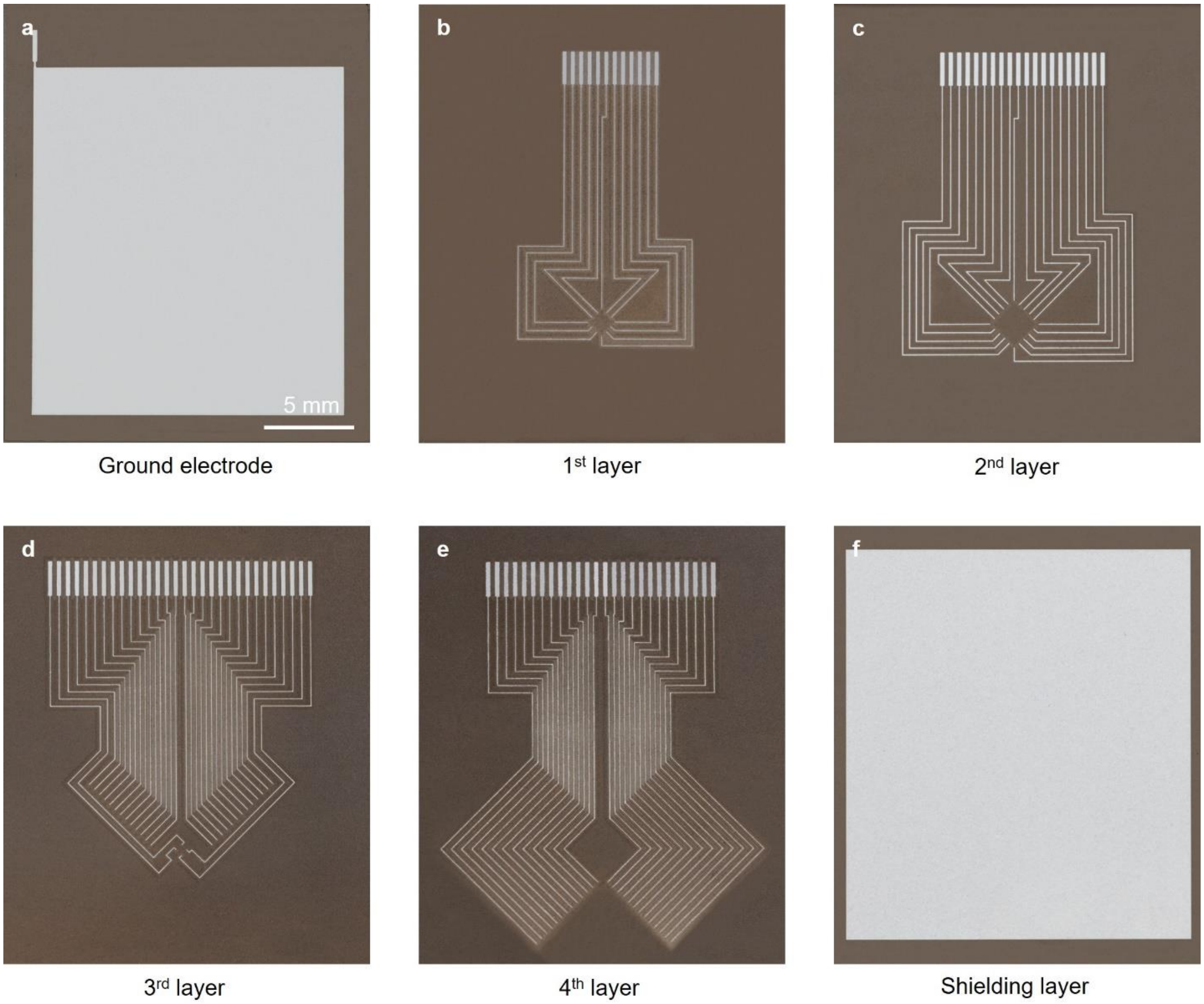


**Supplementary Fig. 6 | Optical images of the multilayered liquid metal composite electrodes.** **a**, Ground electrode. **b**, The first layer. **c**, The second layer. **d**, The third layer. **e**, The fourth layer. **f**, The shielding layer. All images share the same scale bar.

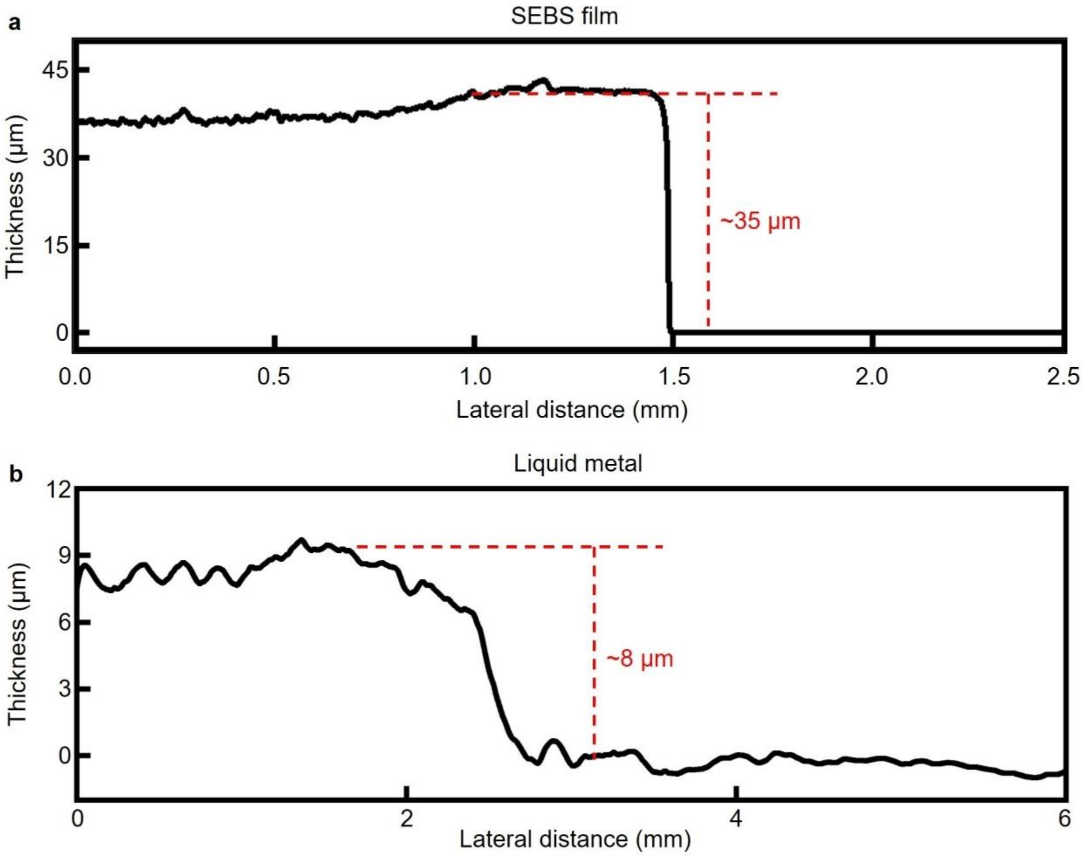


**Supplementary Fig. 7 | The thickness of the SEBS substrate and the printed liquid metal composite.** The thicknesses of **a**, the SEBS film and **b**, the liquid metal composite layer were measured by a Dektak profilometer. The thin thicknesses of the substrate and the electrode contribute to the overall low form-factor of the wearable imager.

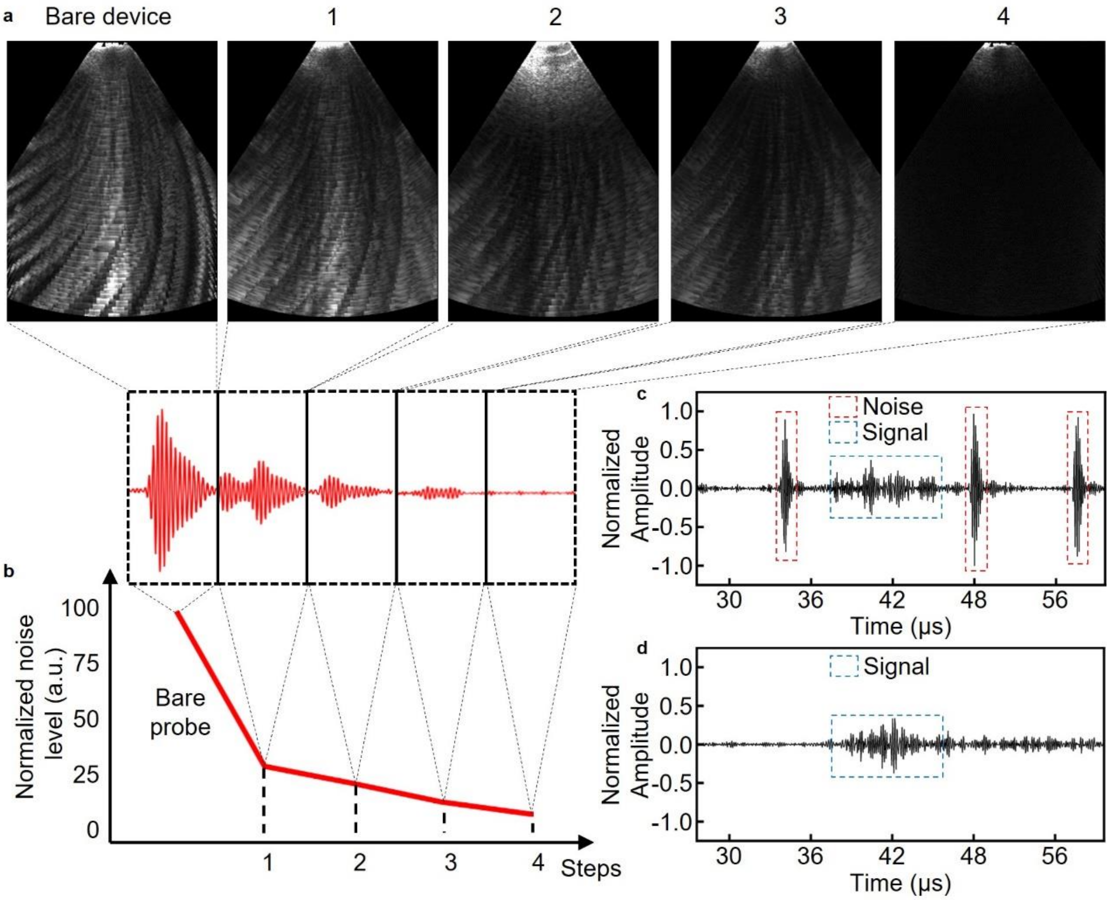


**Supplementary Fig. 8 | Characterization of noise levels after applying different strategies sequentially. a**, B-mode images and **b**, noise amplitude after Step 1: Shielding electrode. Step 2: Electrical matching between the transducer and the pulser. Step 3: Ground wire modification by adding an inductor and capacitor in series to the ground wire. The modification rendered the ground wire to be more resistive at around the transducer center frequency and drain noise at around the transducer center frequency to the ground better. Other noise can be effectively removed by filters. Step 4: Signal accumulation, which is a built-in function provided by Verasonics. The signal accumulation overlaps recent frames to counteract the running noise. We normalized the noise level by dividing all noise levels by the highest noise level for better comparison. **c**, Signals from the cardiac myocardium mixed with noises without shielding. **d**, Signals with much reduced noises after shielding.

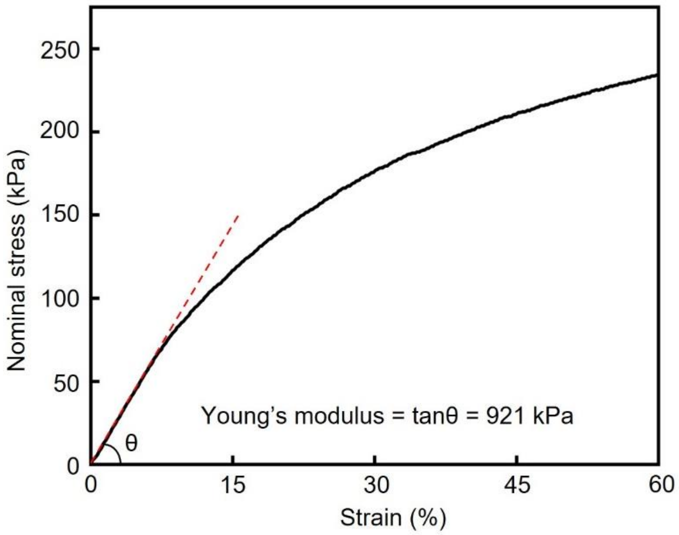


**Supplementary Fig. 9 | Stress-strain curve of the entire device.** The cardiac imager was stretched uniaxially, from which the Young's modulus of the entire device was calculated to be 921 kPa. It showed the device had a similar modulus to the human skin (420 to 850 kPa)[24]. The test was performed under nominal stress, which equals to force divided by initial cross-sectional area of the entire device.

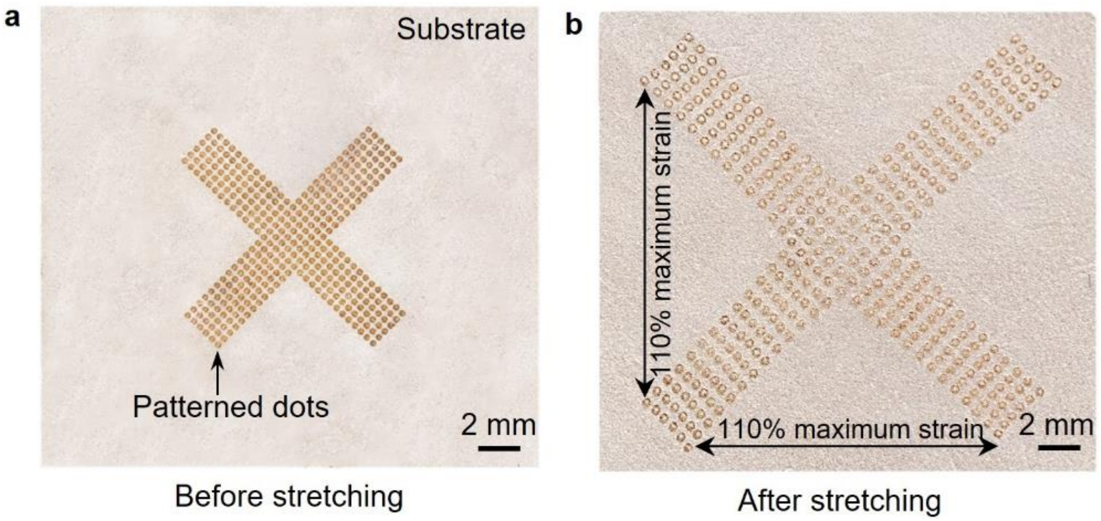


**Supplementary Fig. 10 | Biaxial mechanical testing of the entire device.** Because the shielding and ground layers are opaque, we patterned dots on the SEBS substrate to represent the location of each transducer element. Optical images show the device **a**, before and **b**, after stretching. The spatial distribution of arrayed elements is comparable to that obtained through simulation in Figure 1e, verifying the accuracy of the simulation.

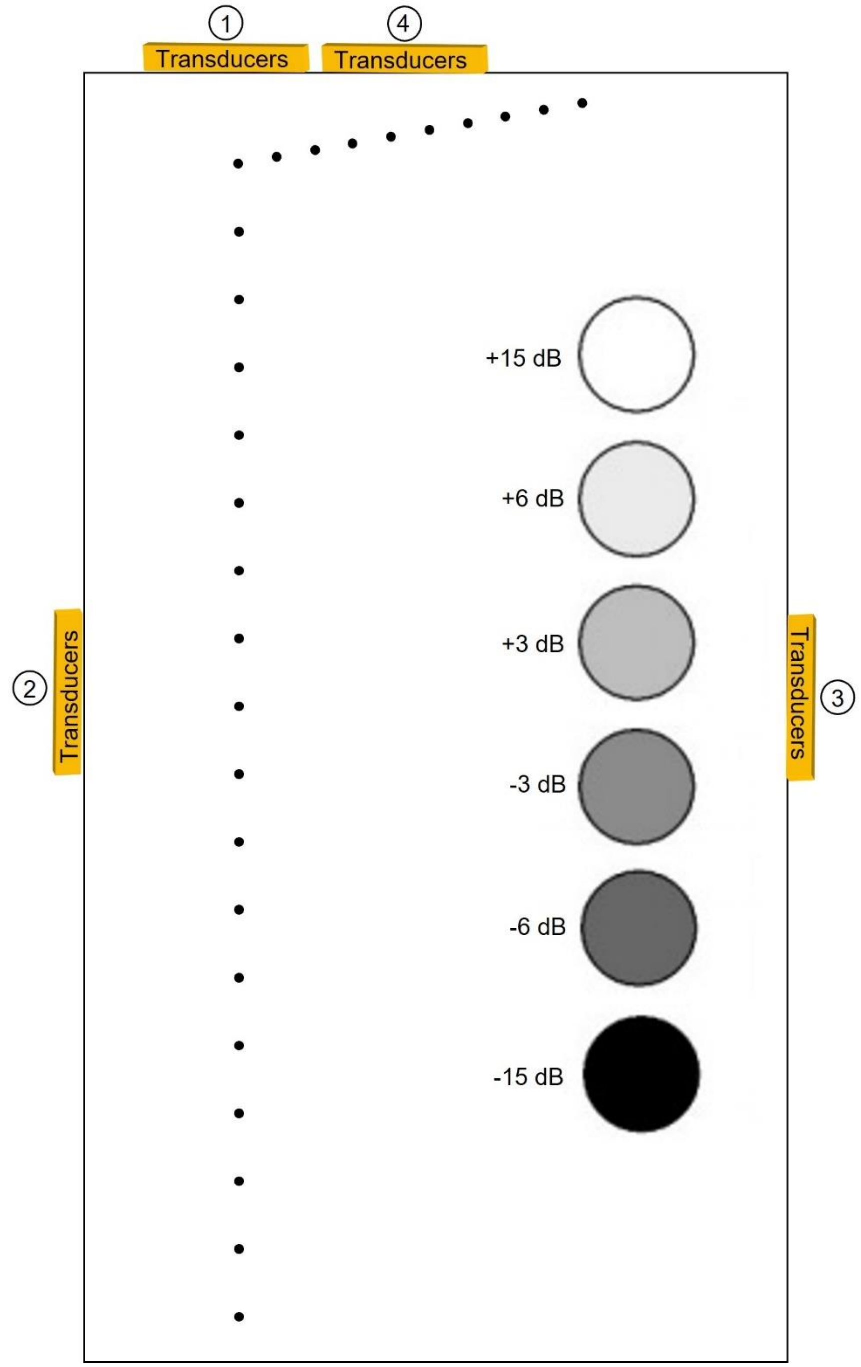


**Supplementary Fig. 11 | The structure of the phantom for device characterizations.** We used a commercial phantom (CIRS 539) to characterize multiple properties of the wearable imager. The signal-to-noise ratio, axial, lateral and elevational resolutions at different depths, and axial accuracy were tested when the device was put at the position 1. The lateral accuracy, as well as axial and lateral resolutions at different lateral distances, were tested when the device was put at the position 2. The dynamic range, contrast-to-noise ratio, and contrast resolution were tested when the device was put at the position 3. The dead zone was tested when the device was put at the position 4.

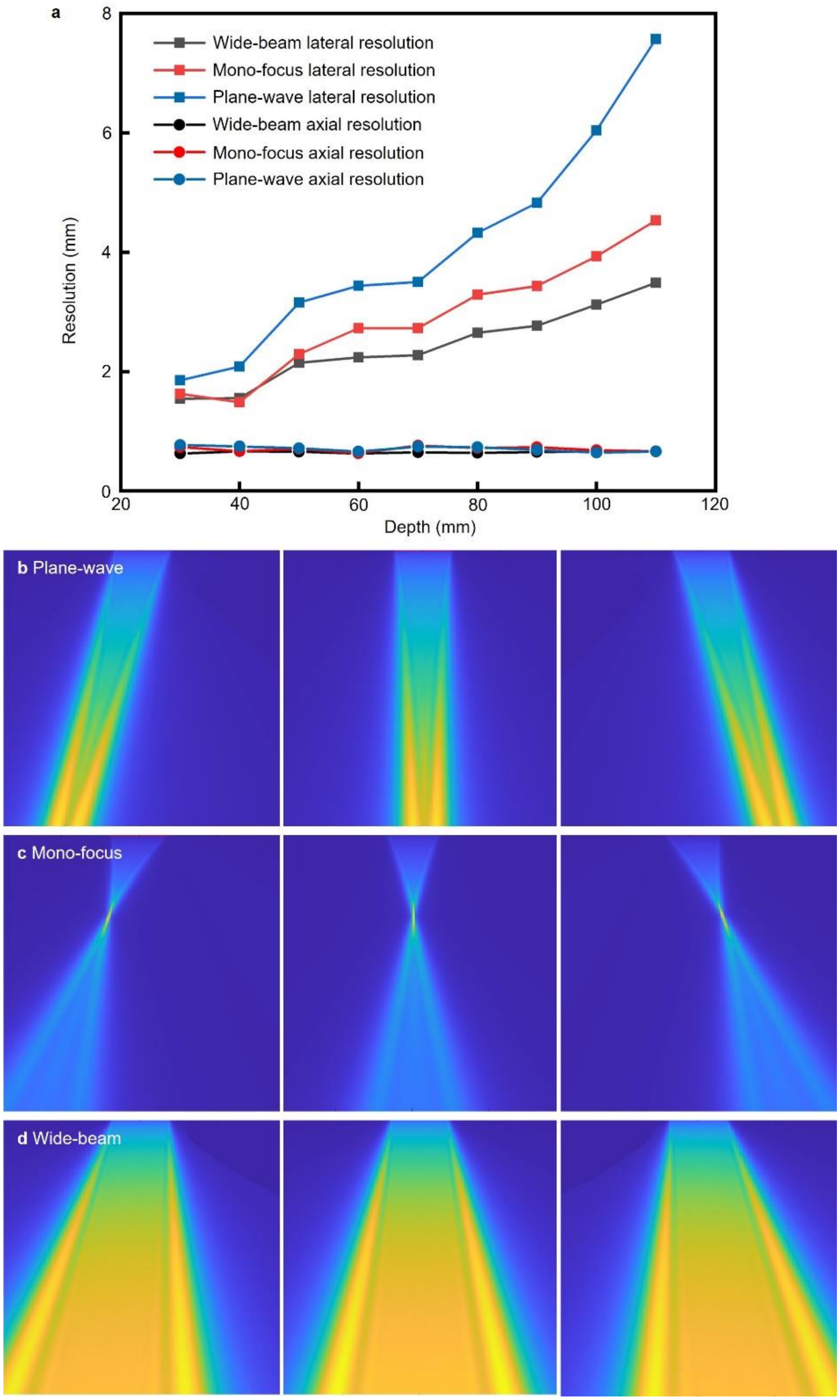


**Supplementary Fig. 12 | Characterization of resolutions and acoustic fields with different transmission methods and angles. a**, The lateral and axial resolutions of wide-beam compounding, mono-focus, and plane-wave transmission[156,228]. The wide-beam compounding

transmission has the best resolutions among all three. Acoustic fields of **b**, plane-wave, **c**, mono-focus, and **d**, wide-beam compounding transmission methods, with transmission angles of -21°, 0°, and 21°. The plane-wave strategy produces the worst resolutions. This is because this mode only transmits a single plane-wave, resulting in a low echoic signal-to-noise ratio, and poor spatial resolutions. The mono-focus strategy yields a greater signal-to-noise ratio, and better spatial resolutions. However, the resolution will deteriorate outside the focal zone. The wide-beam compounding transmission has a stronger and more uniform acoustic wave intensity over a larger area than the other two.

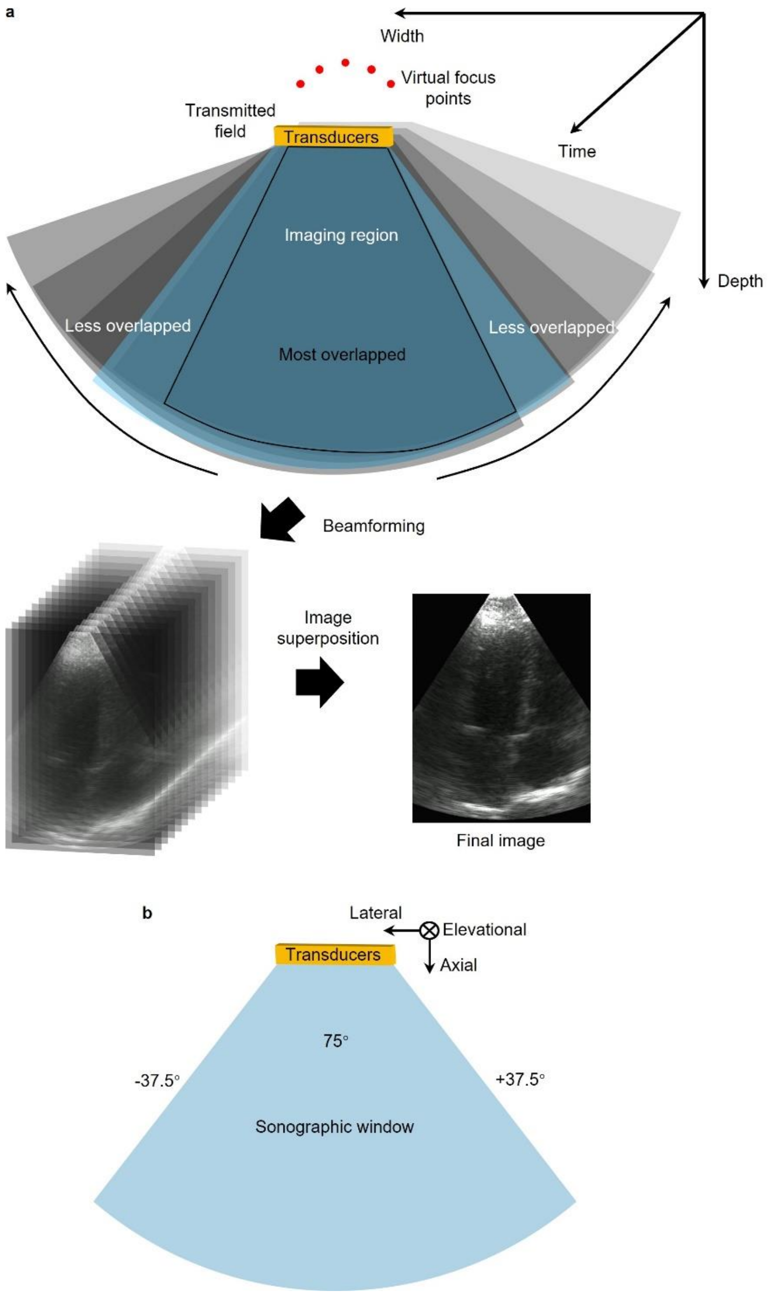


**Supplementary Fig. 13 | The mechanism of wide-beam compounding B-mode imaging. a,** Multiple frames are first acquired with multiple transmissions at different angles. The multiple-angle scan compensates the low echoic energies from regions away from the center, expanding the insonation area from being rectangular to sector-shaped. The enhanced echoic energy improves the resolution at high steering angles. The frames are collected at the same rate as the high pulse repetition frequency. The final image is obtained by the superposition of acquired frames, which achieve synthetic focusing with improved resolution over the entire ultrasonographic window. Additionally, the superposition helps eliminate the random noise in the images. **b**, Schematics of the acoustic field simulation setup. All key parameters are labeled and set the same as the practical imaging procedure.

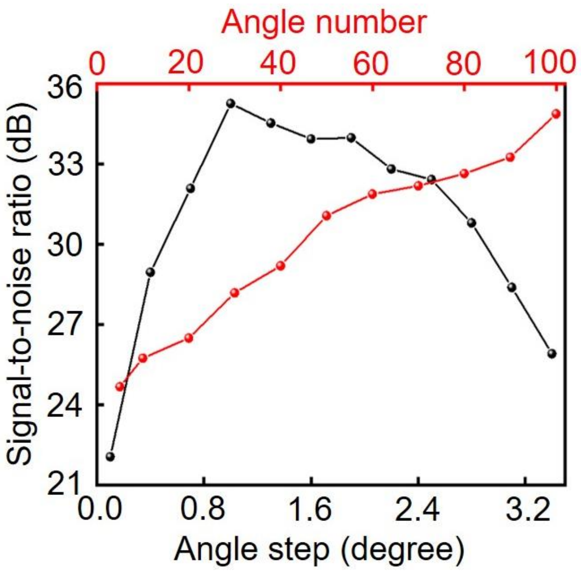


**Supplementary Fig. 14 | Signal-to-noise ratio as a function of step size and number of steering angles of the wide-beam compounding imaging.** The image's signal-to-noise ratio firstly rises and then falls with the angle step size but increases monotonically with the angle number. When the angle step size initially increases, the most constructive interference of multiple acoustic fields is in the region of interest, ensuring the highest signal-to-noise ratio for the image reconstruction. As the angle step size keeps increasing, the overlap between individual acoustic fields decreases, resulting in a reduced signal-to-noise ratio. The signal-to-noise ratio increases as the number of angles grows, because all individual acoustic fields are more or less coherently integrated to reconstruct images. However, an excessive number of angles sacrifices the imaging temporal resolution. We used 96 steering angles with a 1° step, which gives adequate penetration depth and spatial resolutions while maintaining an acceptable frame rate of 20-30 Hz.

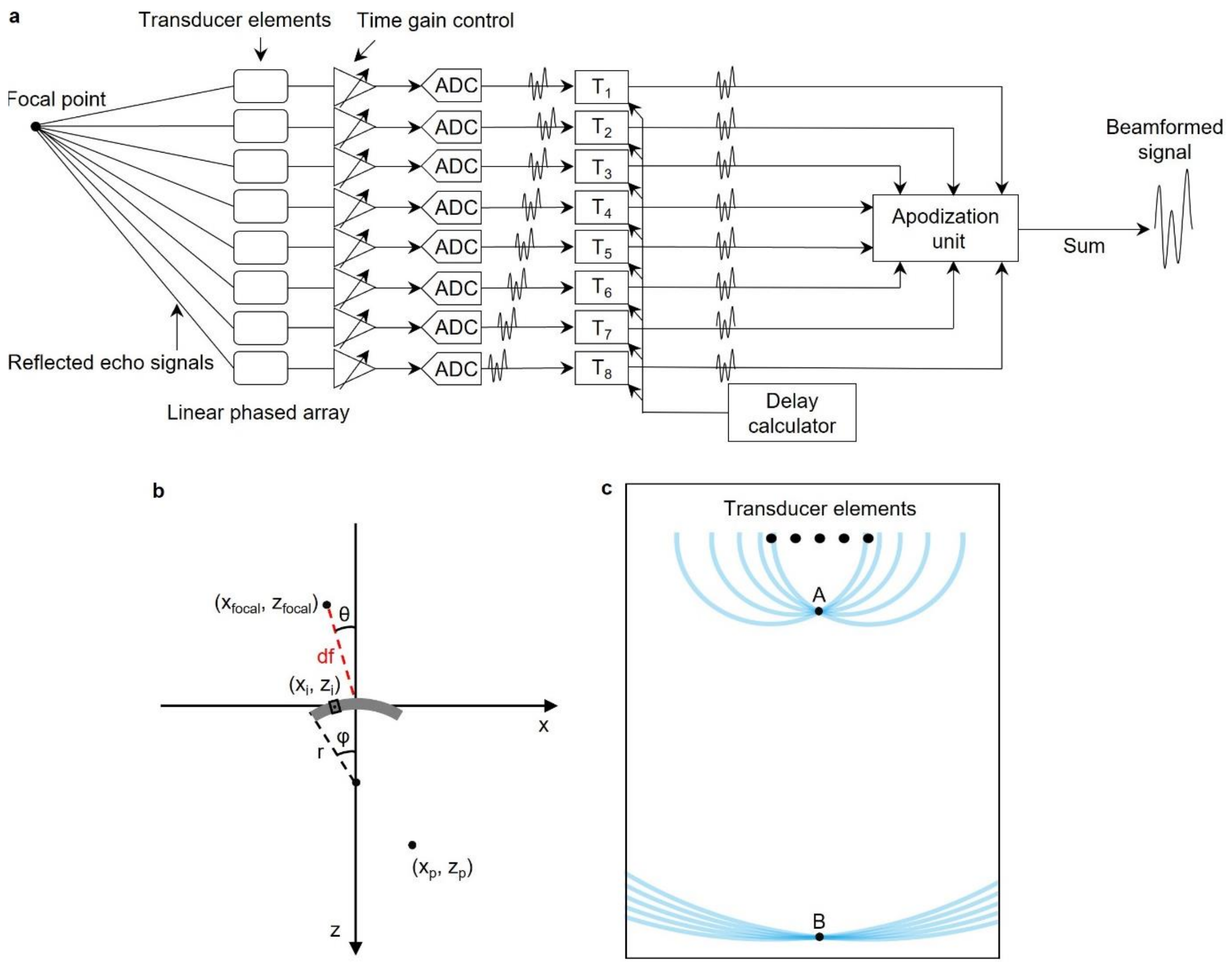


**Supplementary Fig. 15 | The flow chart of receive beamforming. a**, The reflected echoes are received by the transducer elements, whose signals are amplified by the time gain control to enhance the weak signals from deep objects. The amplified signals are then converted to digital signals by an analog to digital converter (ADC), and then sent into a delay calculator for phase difference correction and signal alignment. Direct summing of the synchronized signals may result in significant side-lobe artifacts. Therefore, adaptive apodization assigns varying weights to the various signals, which are eventually summed together as beamformed signals with an enhanced signal-to-noise ratio. **b**, Schematic calculation for phase correction. ($x_{focal}$, $z_{focal}$) is the focal point. ($x_i$, $z_i$) is the $i^{th}$ transducer. ($x_p$, $z_p$) is the pixel of interest. θ is the steering angle. df is the focal depth. r is the curvature radius. φ is the angle departure of the $i^{th}$ transducer from z-axis on the curvature. **c**, Schematic receive beamforming of ultrasound signals. There are two beamformed signals A and B. The lateral diffusion in A is less than that in B, which indicates a better lateral resolution of A. In other words, the closer the imaging area to the transducer elements, the better the lateral resolution.

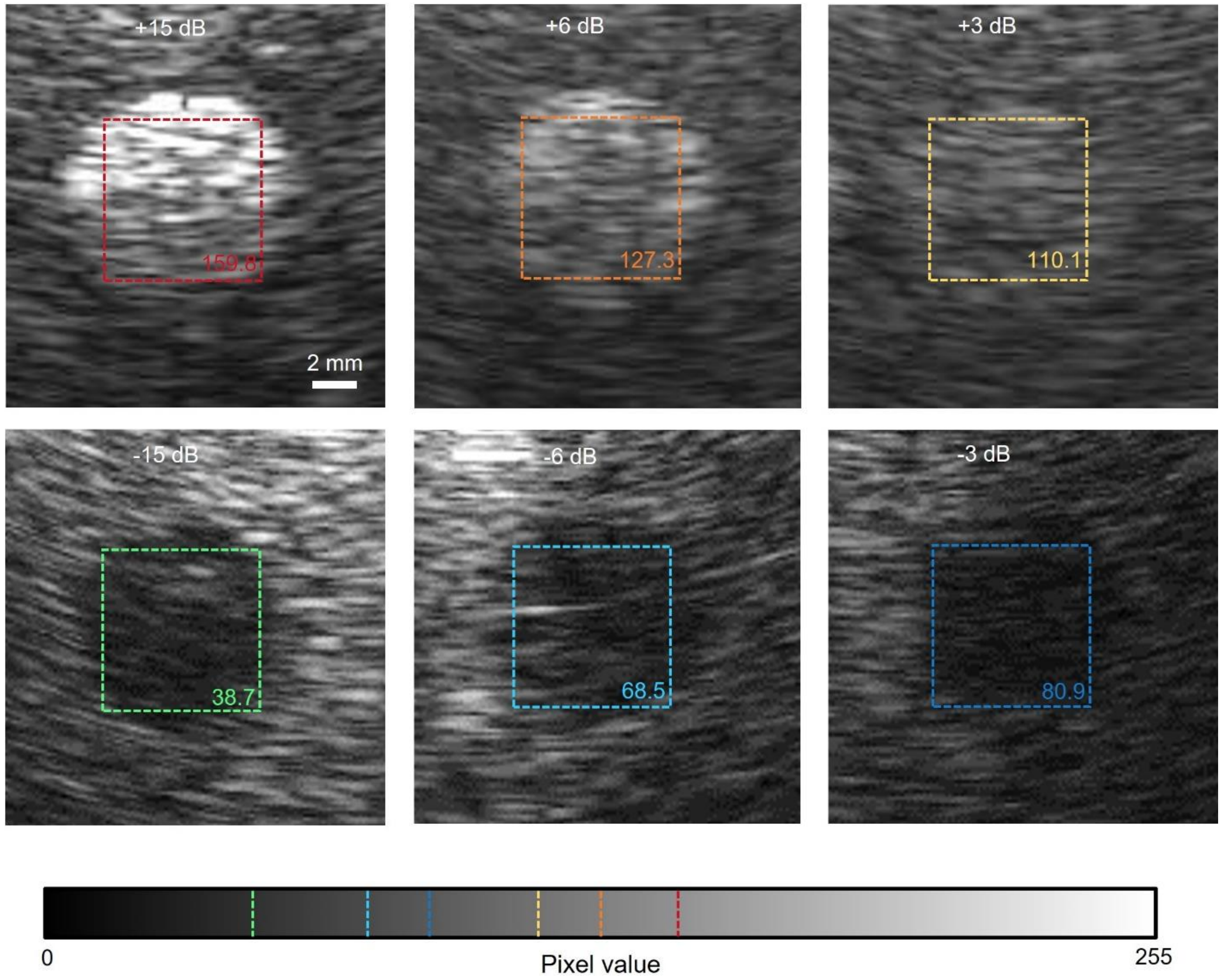


**Supplementary Fig. 16 | Gray scale B-mode images of phantoms and selected windows for calculating the dynamic range.** Red: The +15 dB contrast gives an average pixel value of 159.8. Orange: The +6 dB contrast gives an average pixel value of 127.3. Yellow: The +3 dB contrast gives an average pixel value of 110.1. Green: The -15 dB contrast gives an average pixel value of 38.7. Cyan: The -6 dB contrast gives an average pixel value of 68.5. Blue: The -3 dB contrast gives an average pixel value of 80.9. These pixel values are labelled on the pixel scale bar at the bottom. The dynamic range is thus calculated to be 63.2 dB, which is well above the 60 dB threshold usually used in medical diagnosis to give adequate details of the echo patterns in the images.

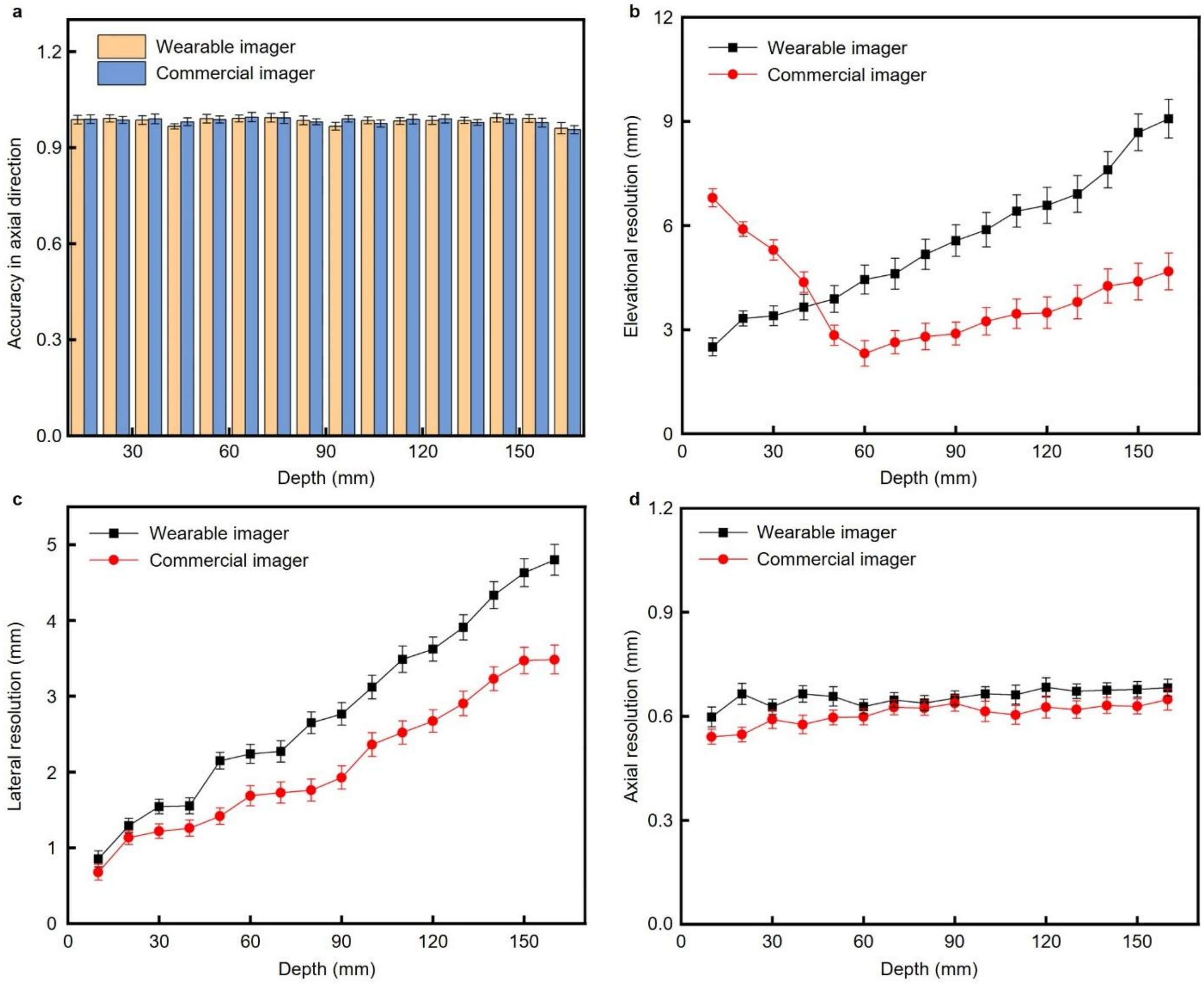


**Supplementary Fig. 17 | Detailed comparison of the imaging metrics between the wearable and the commercial imagers. a**, Accuracy of the detected scatter positions as a function of the scatter depth. **b**, Elevational resolution as a function of depth. **c**, Lateral resolution as a function of depth. **d**, Axial resolution as a function of depth. Each test was repeated for five times. Each column or point is defined by the mean and the standard deviation (the error bar) of the results from five tests.

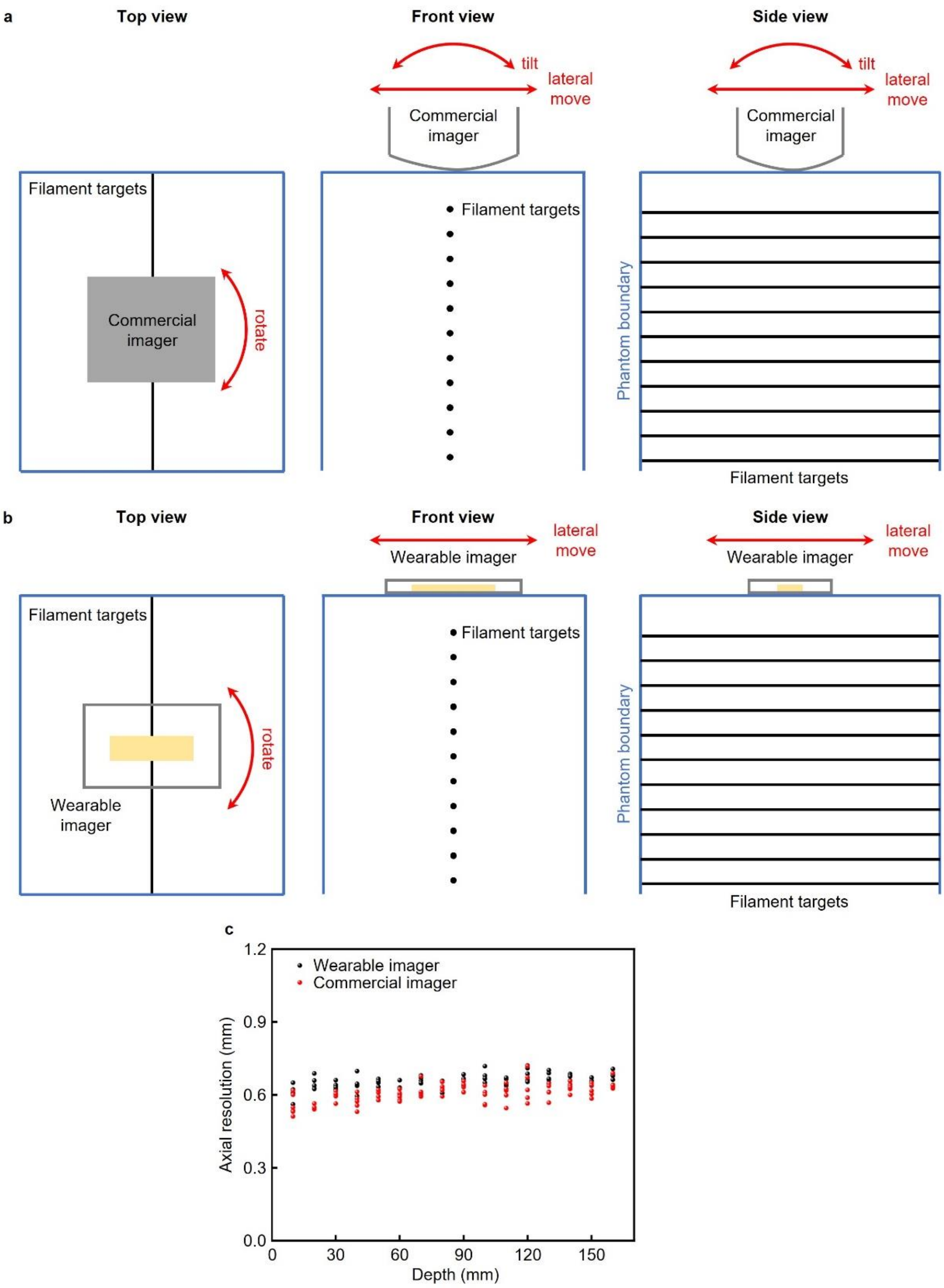


**Supplementary Fig. 18 | Schematic experimental setups of resolution tests.** The setups are for **a**, the commercial imager and **b**, the wearable imager. The degrees of freedom of each imager are

labeled in red arrows. **c**, The axial resolutions of the wearable imager and the commercial imager at each depth were measured five times. The five degrees of freedom of the commercial imager and the three degrees of freedom of the wearable imager introduced measurement variations. The higher the degrees of freedom, the higher the possibility of worsening the measured axial resolutions. Even though the commercial imager bandwidth is larger than that of the wearable imager, some data points of the commercial imager are worse than those of the wearable imager because of the measurement variations.

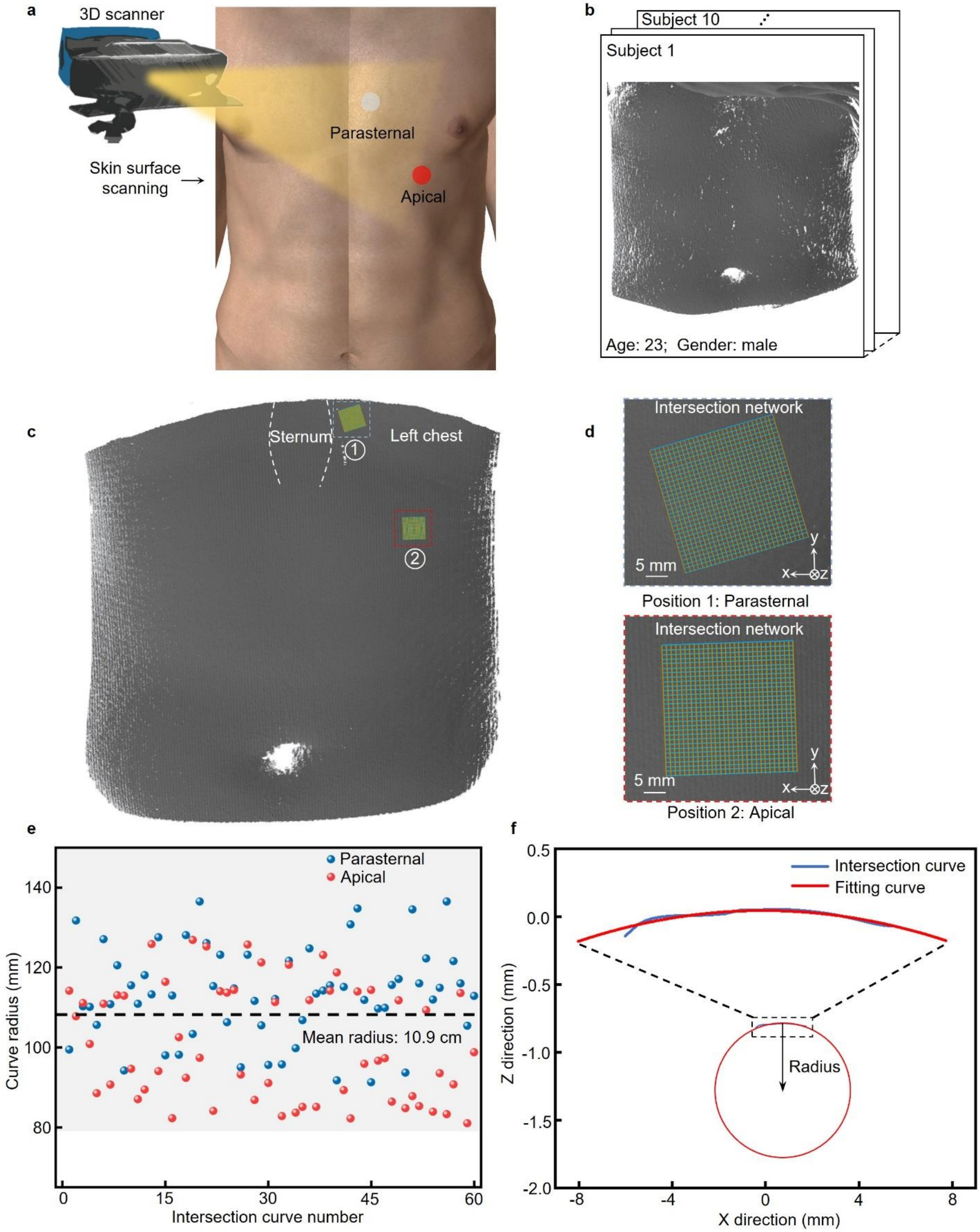


**Supplementary Fig. 19 | Processes of evaluating the surface curvature for phase correction.** **a**, Scan the imaging sites on the subject using a 3D scanner. **b**, Obtain 3D surface reconstruction from the scanning. **c**, Select the two sites of interest in this study and build intersection networks

in the Catia software. **d**, Zoomed-in schematics of the two intersection networks from a reconstructed 3D surface. **e**, Collect the average curve radii from curves in the intersection networks. **f**, Fit every intersection curve with a smooth curve. Select the intersection curve whose fitting radius is the closest to the mean radius of all curves from the site. The fitting curve is then used to correct the phased distortion induced by the surface curvature of human body.

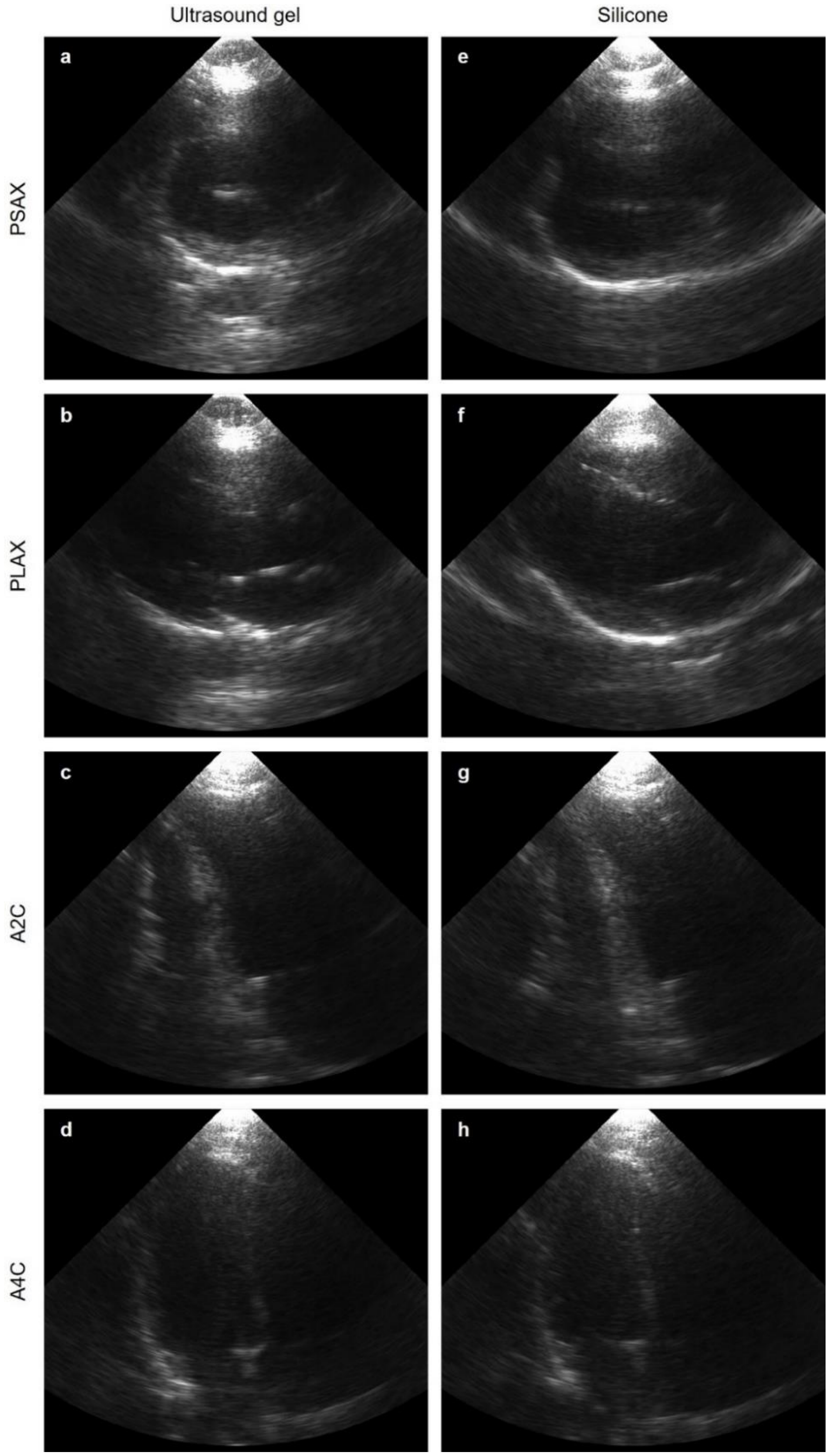


**Supplementary Fig. 20 | B-mode images collected with different couplants. a-d**, The PSAX, PLAX, A2C and A4C views collected with evaporative ultrasound gel. **e-h**, The PSAX, PLAX, A2C and A4C views collected with non-evaporative silicone. No obvious structural differences are found in the comparison. PSAX: parasternal short axis view; PLAX: parasternal long axis view; A2C: apical two-chamber view; A4C: apical four-chamber view.

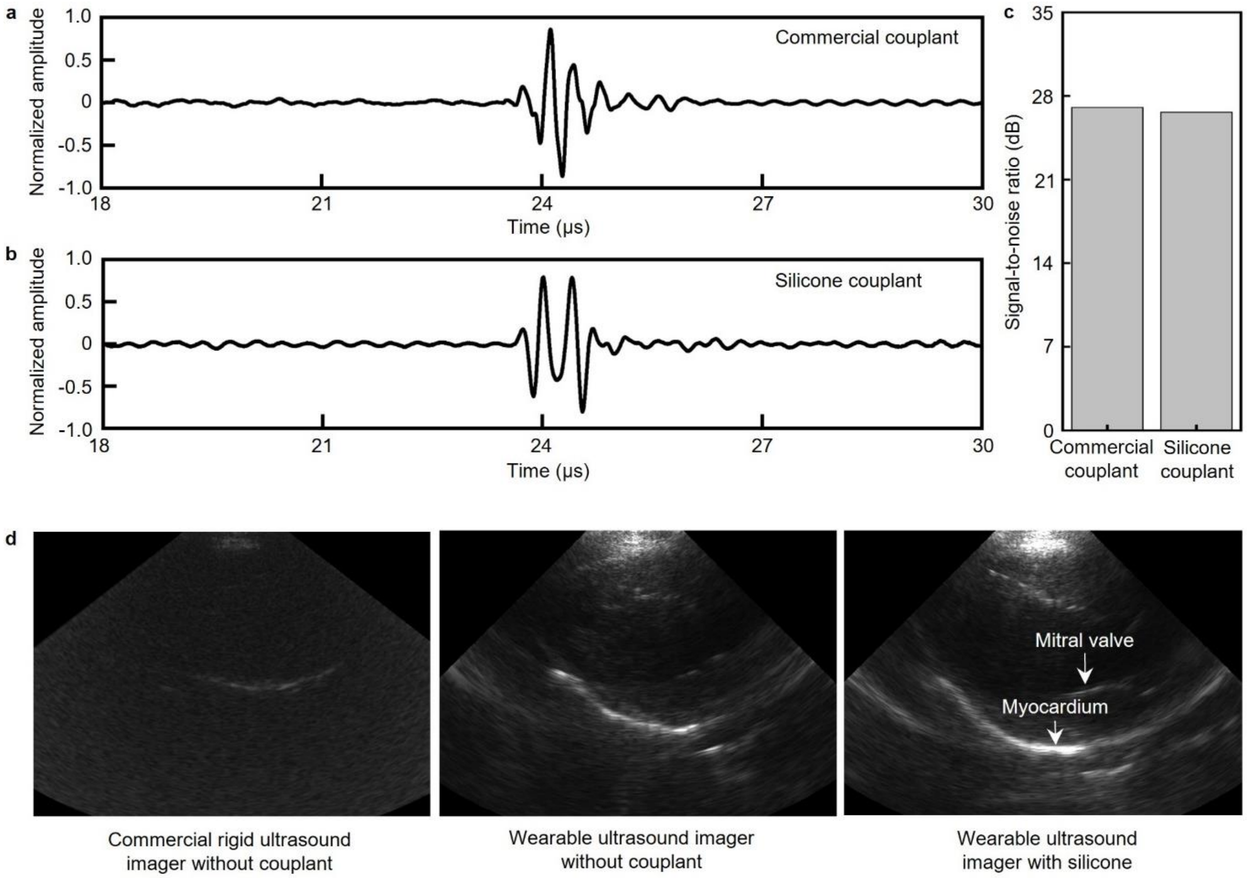


**Supplementary Fig. 21 | Quantitatively evaluation of different coupling conditions.** Comparison of the coupling performance between commercial ultrasonic gel and liquidous silicone couplants. Received signals of the single transducer with **a**, commercial ultrasonic gel, and **b**, liquidous silicone couplant. **c**, The two couplants result in comparable signal-to-noise ratios of the received signals. **d**, Cardiac images taken under different coupling conditions. The left panel shows the image from a commercial rigid ultrasound imager without couplant. The middle panel shows the image from a wearable ultrasound imager without couplant. The right panel shows the image from a wearable ultrasound imager with silicone couplant.

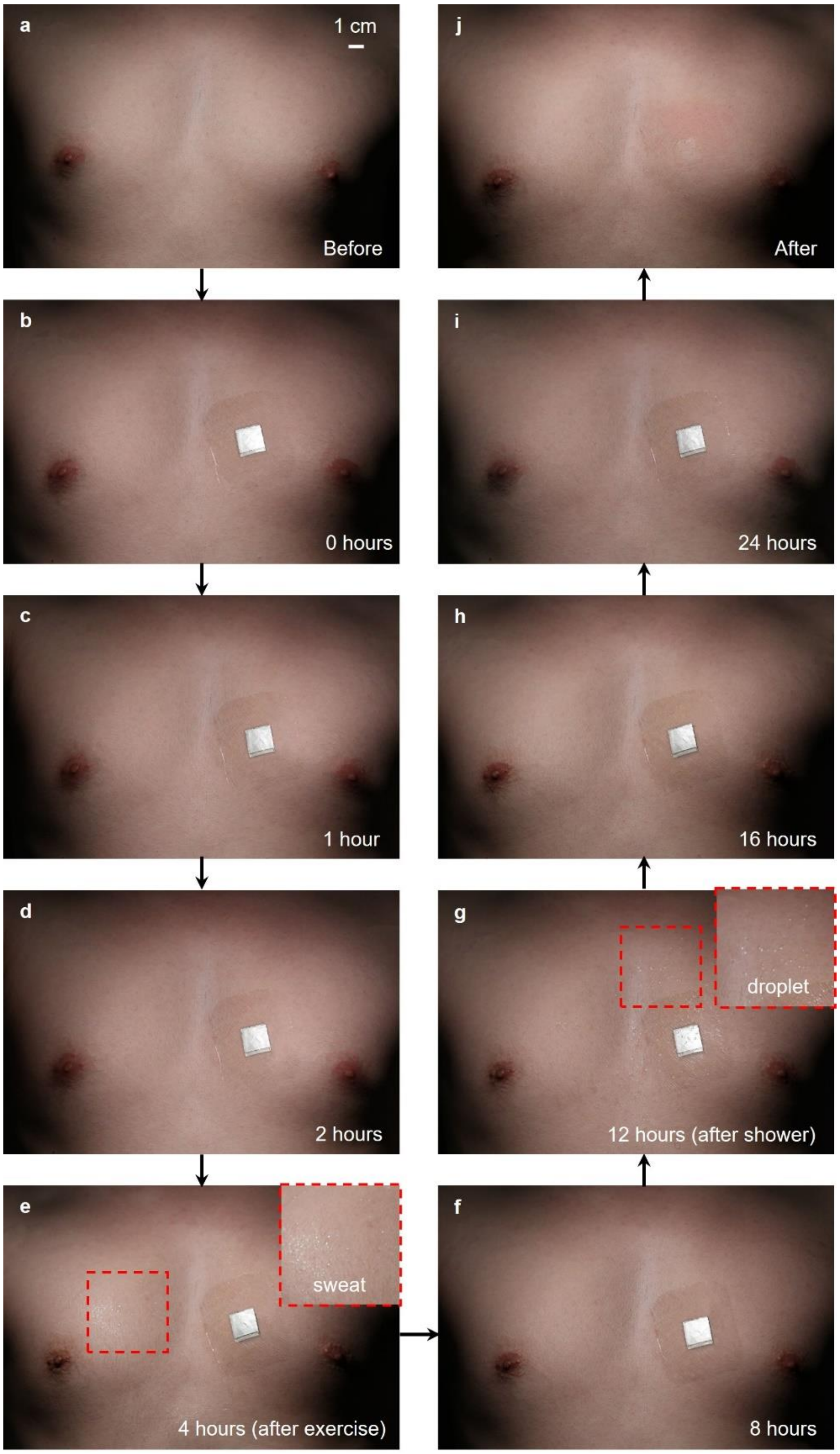


**Supplementary Fig. 22 | Optical images of attaching the wearable imager to the chest for long-term.** Optical images of the chest **a**, before attaching the probe, **b-i**, 0, 1, 2, 4, 8, 12, 16, and 24 hours after the attachment, and **j**, after detaching the imager from the human body. Sweat droplets can be seen in the zoomed-in inset in **e**, after the subject finished working out. Water droplets can be seen in the zoomed-in inset in **g**, after the subject finished showering.

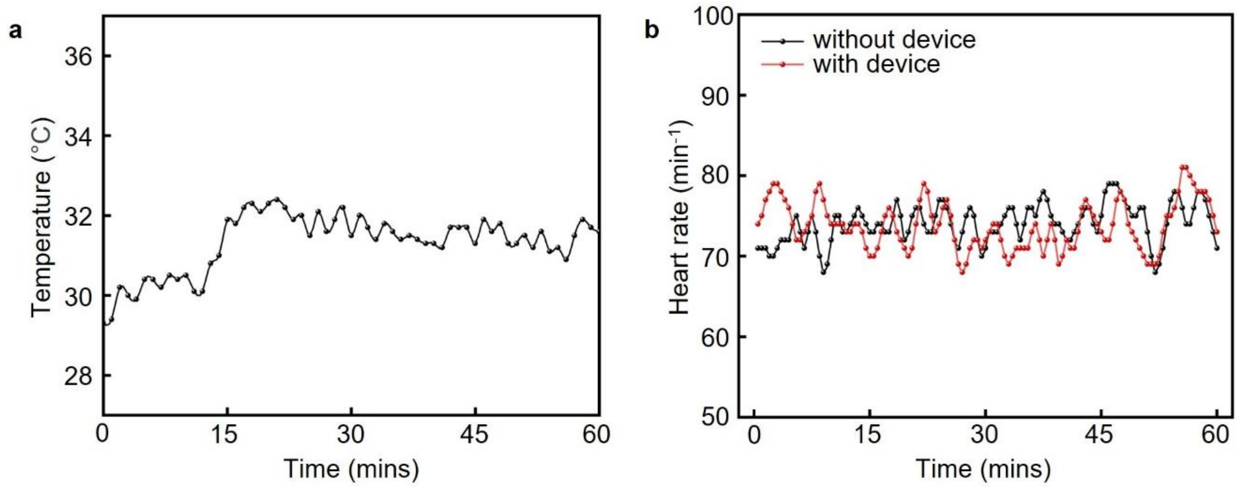


**Supplementary Fig. 23 | Continuous surface temperature and heart rate monitoring for 1 hour. a**, Recording the surface temperature of the device by a thermal camera every minute for 1 hour. The highest temperature is ~33 °C, which is harmless to the human body. **b**, Monitoring the heart rate using a oximeter every half minute with and without the device attachment. No obvious difference is observed, showing the safety of the wearable cardiac imager for long-term monitoring.

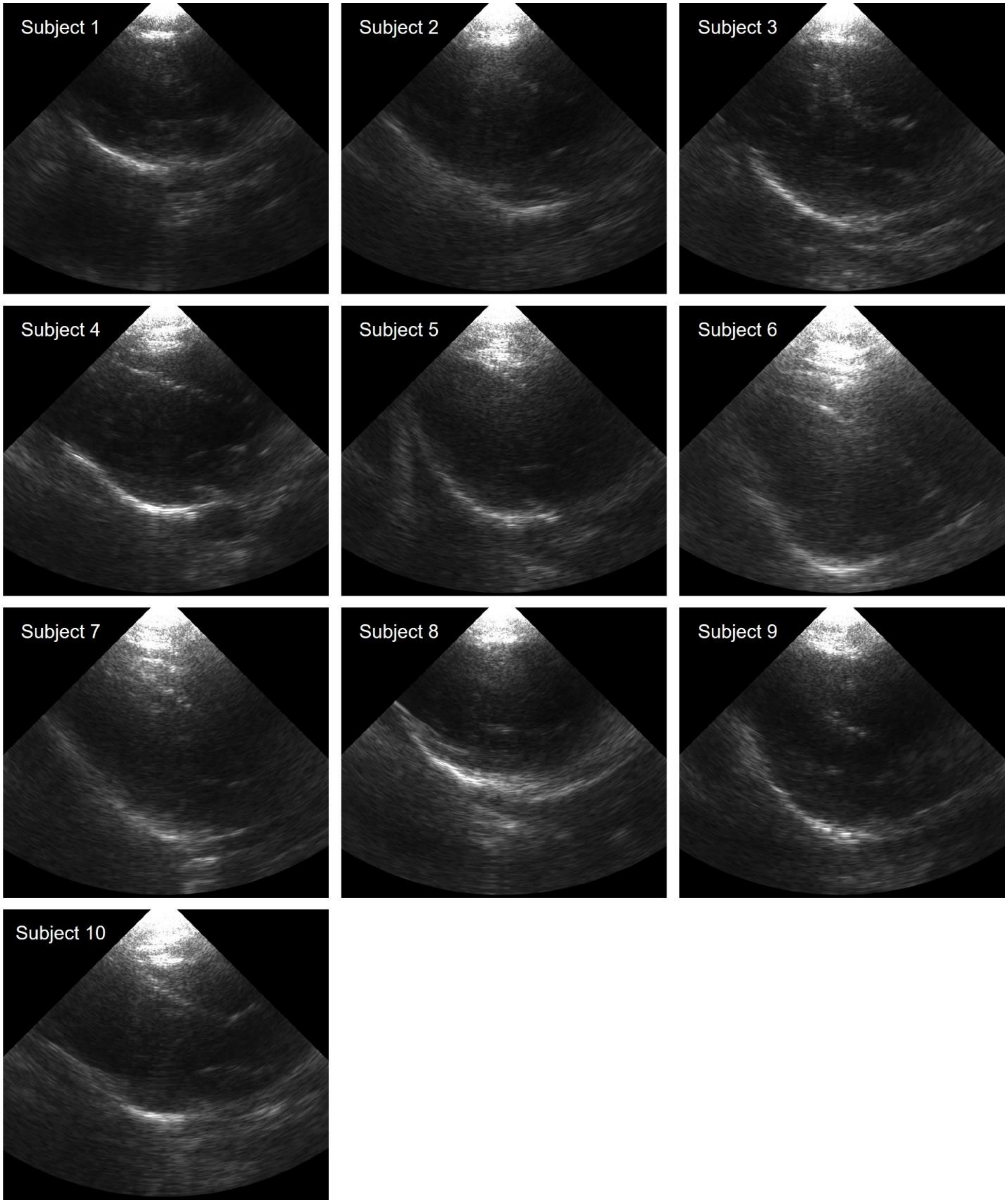


**Supplementary Fig. 24 | Images of the parasternal long axis view from 10 subjects using a recycled device.** Expanding the testing cohort size validates the reproducibility and reliability of the wearable imager.

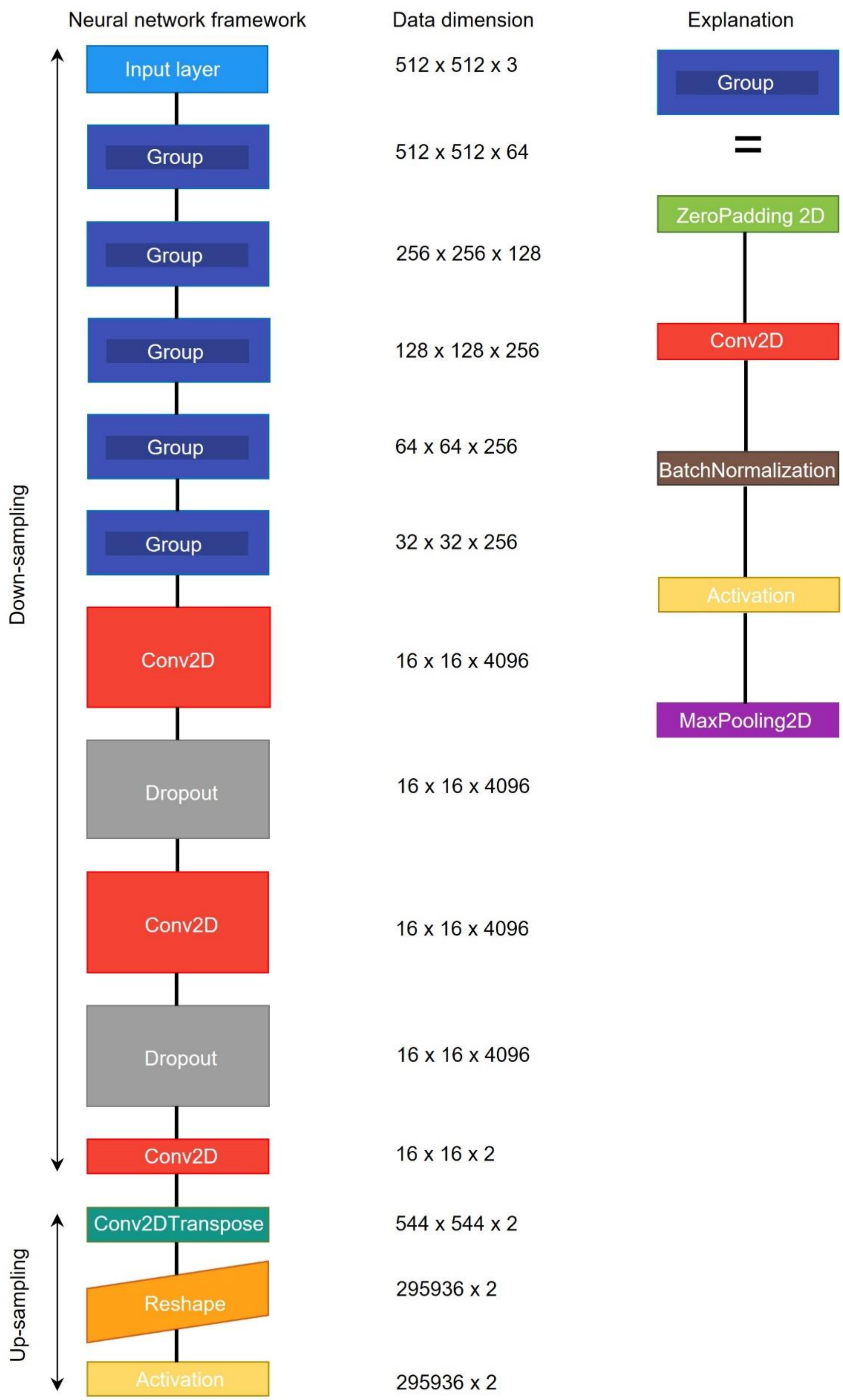


1965 **Supplementary Fig. 25 | The structure of the FCN-32 neural network.** The FCN-32's structure

includes a series of fully-connected convolutional neural network (CNN) layers and an upsampling layer at the end. We used the AlexNet structure for the downsampling process. The input layer is first connected to five groups of connected CNN layers, then connected to two additional CNN-dropout bilayers, and finally connected to an upsampling layer to restore to its original size. Specifically, the five CNN groups have similar structures but different dimensions. The input of each group is first zero-padded and sent into a 2D convolutional layer. Then, we used batch normalization to standardize the CNN's output and activate it with a Rectified Linear Unit and downsample it with max-pooling.

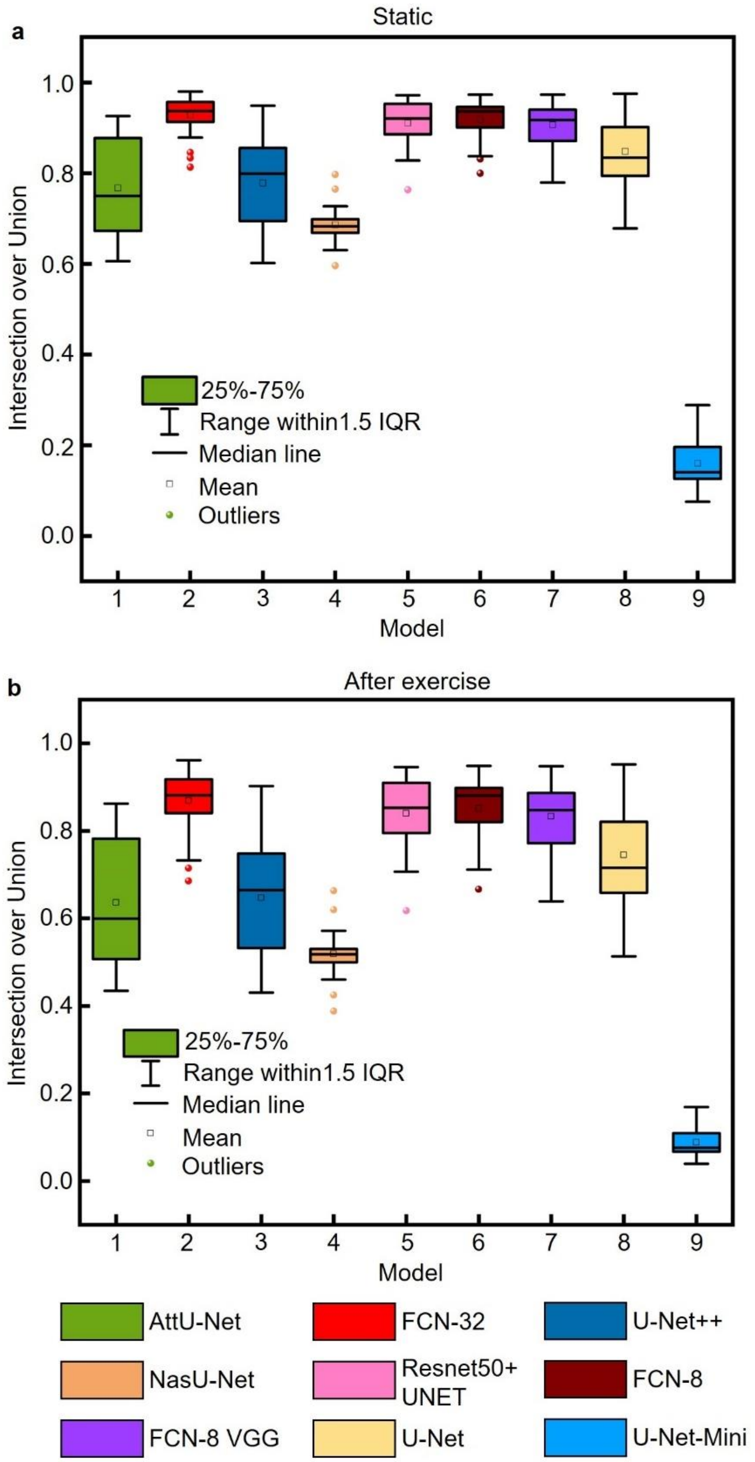


**Supplementary Fig. 26 | The comparison of the intersection over union among different models used in this study.** The comparison is made **a**, before and **b**, after exercise. For a pair of predicted images and manually-labelled ground truth image, its intersection over union equals to the number of pixels that are classified as within the left ventricle in both images divided by the total number of unique pixels that are classified as within the left ventricle by either of the two images. The figure shows each model's Intersection over Union on a testing dataset. The FCN-32

performs the best with the highest intersection over union and its variation is among the lowest. 1.5 interquartile range (IQR) is a common rule in statistics to differentiate the outliers. Data points outside of this range are regarded as outliers.

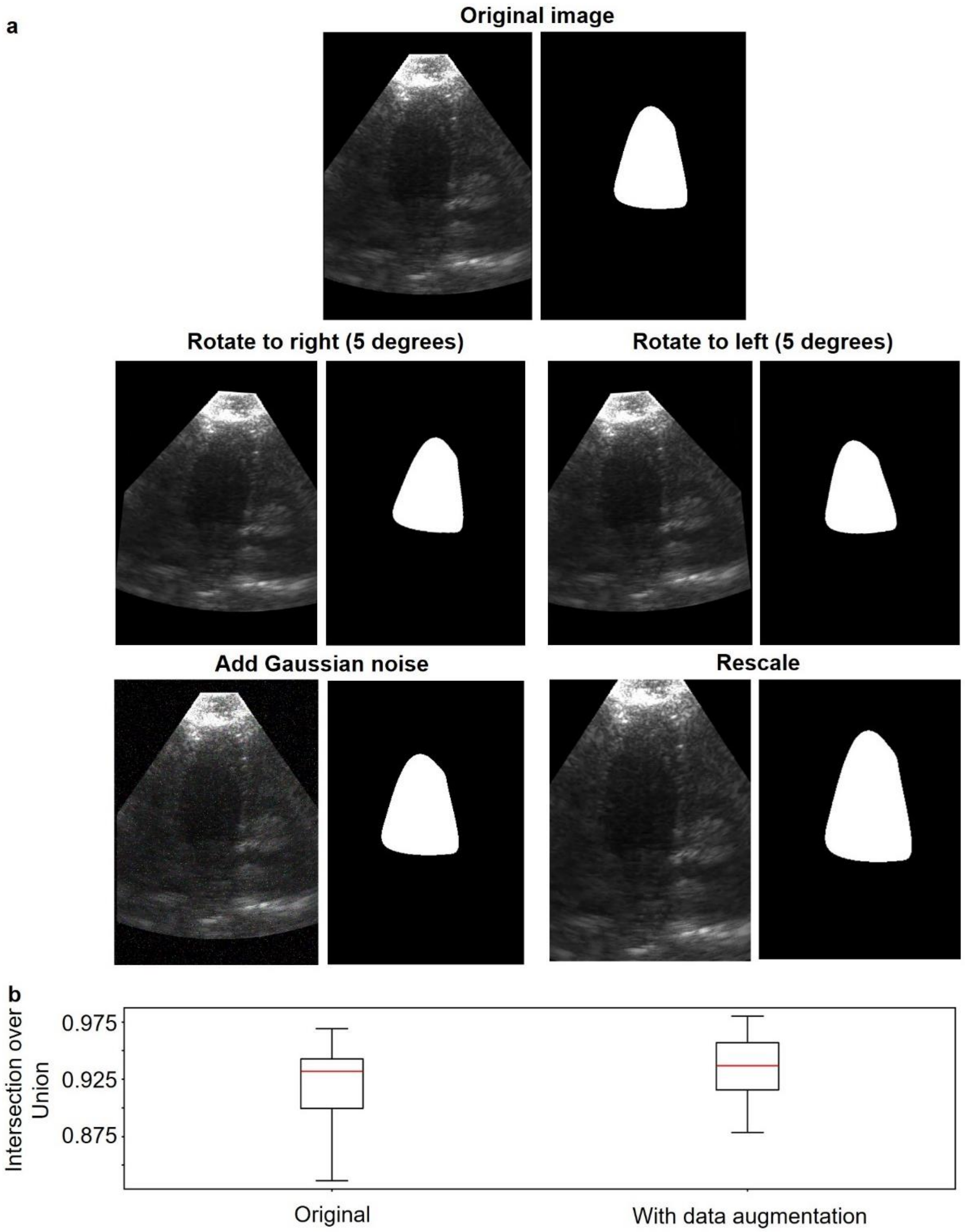


**Supplementary Fig. 27 | The types and results of data augmentation. a**, Four types of data augmentation and corresponding segmentation results. We applied rotation, scaling, and gaussian noise to the data to augment the size of the dataset. **b**, The data augmentation increases the average and reduces the variation of mean intersection over union.

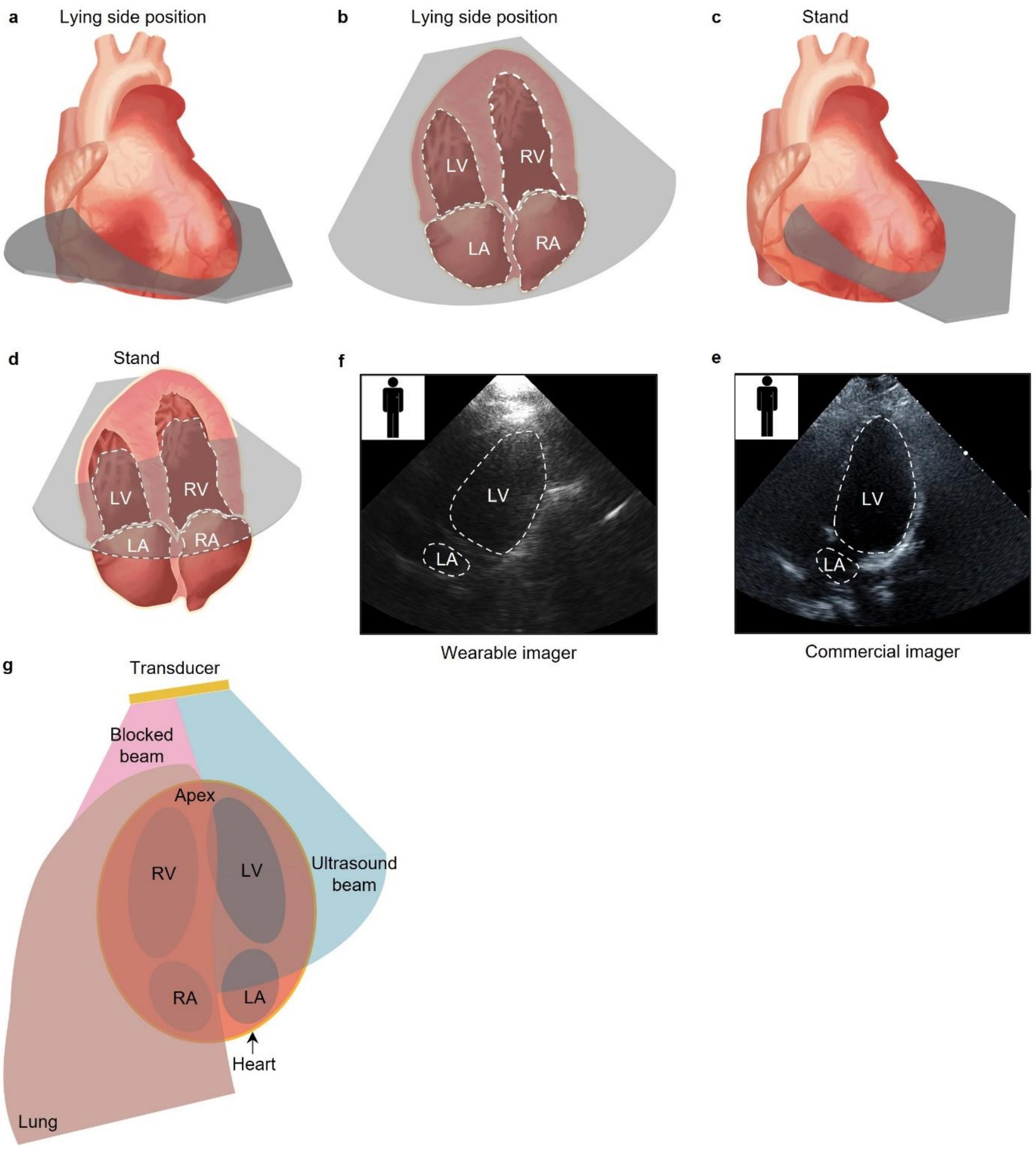


**Supplementary Fig. 28 | Imaging from apical four chamber view with different positions. a**, and **b**, showing schematics of the apical four chamber view in the lying side position. The ultrasound beam enters the heart directly from the apex, and the morphology of four chambers can be fully scanned (white dash box in **b**), which will result in accurate calculation of left ventricle volume and cardiac indices. **c**, and **d**, showing the apical four chamber view in the standing position. The ultrasound beam enters the heart obliquely from the apex, causing incomplete imaging of four chambers (white dash box in **d**). **e**, showing an image from a commercial imager tested by an experienced cardiac sonographer. **f**, showing an image from a wearable ultrasound imager by a trained amateur. Left ventricle (LV) and left atrium (LA) can be roughly seen in both

images. **g**, Because a part of the transducer array was blocked by the lung, only the unblocked part could send ultrasound to the LV and LA, which led to a low signal-to-noise ratio and a lower image quality in the standing position than that in the lying side position. Both the right ventricle and right atrium were mostly obscured due to lung shadow.

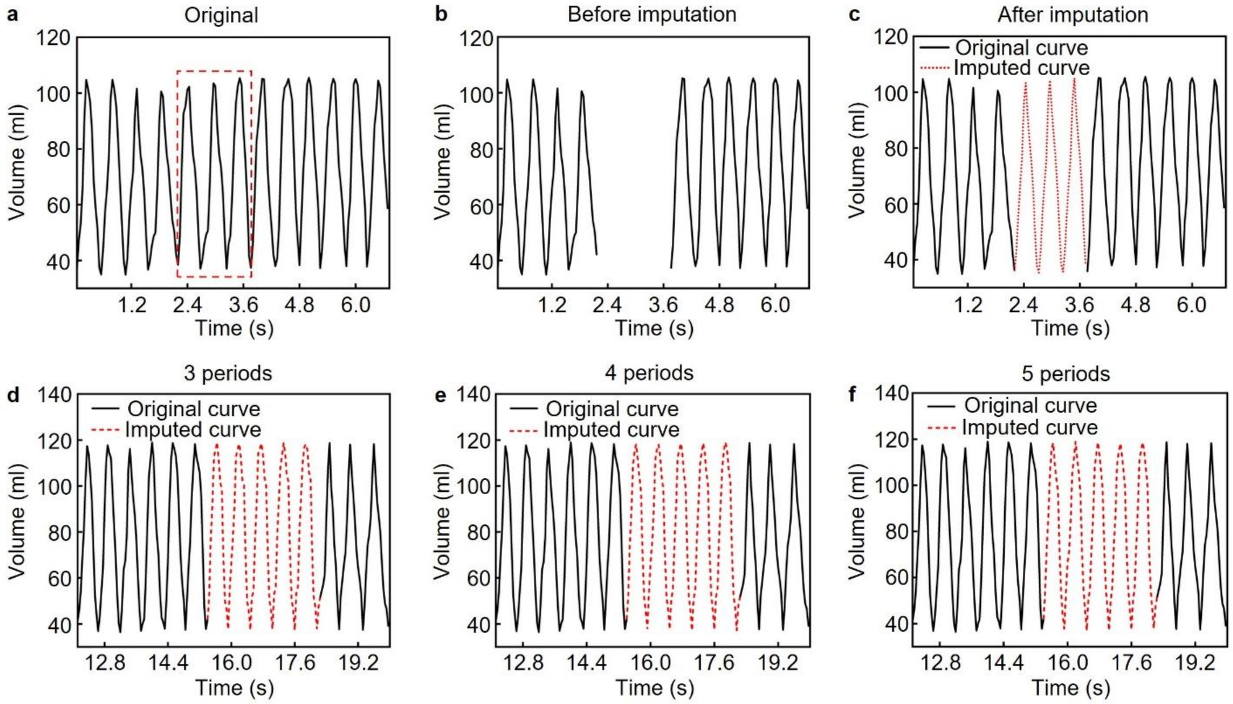


**Supplementary Fig. 29 | Validation of the image imputation algorithm.** To validate the reliability of this image imputation algorithm, we manually erased three periods from **a**, an originally continuous volume wave to get **b**, the erased result before imputation. After imputation, **c**, the completed new wave has a good agreement with the original one with a 0.93 Pearson correlation coefficient. To define the hyperparameter N, we compared different imputation results with **d**, N=3, **e**, N=4, and **f**, N=5. The results show the differences between the generated waves from various N numbers are negligible. We decide to use N=3 in practice for its simplicity and algorithm efficiency.

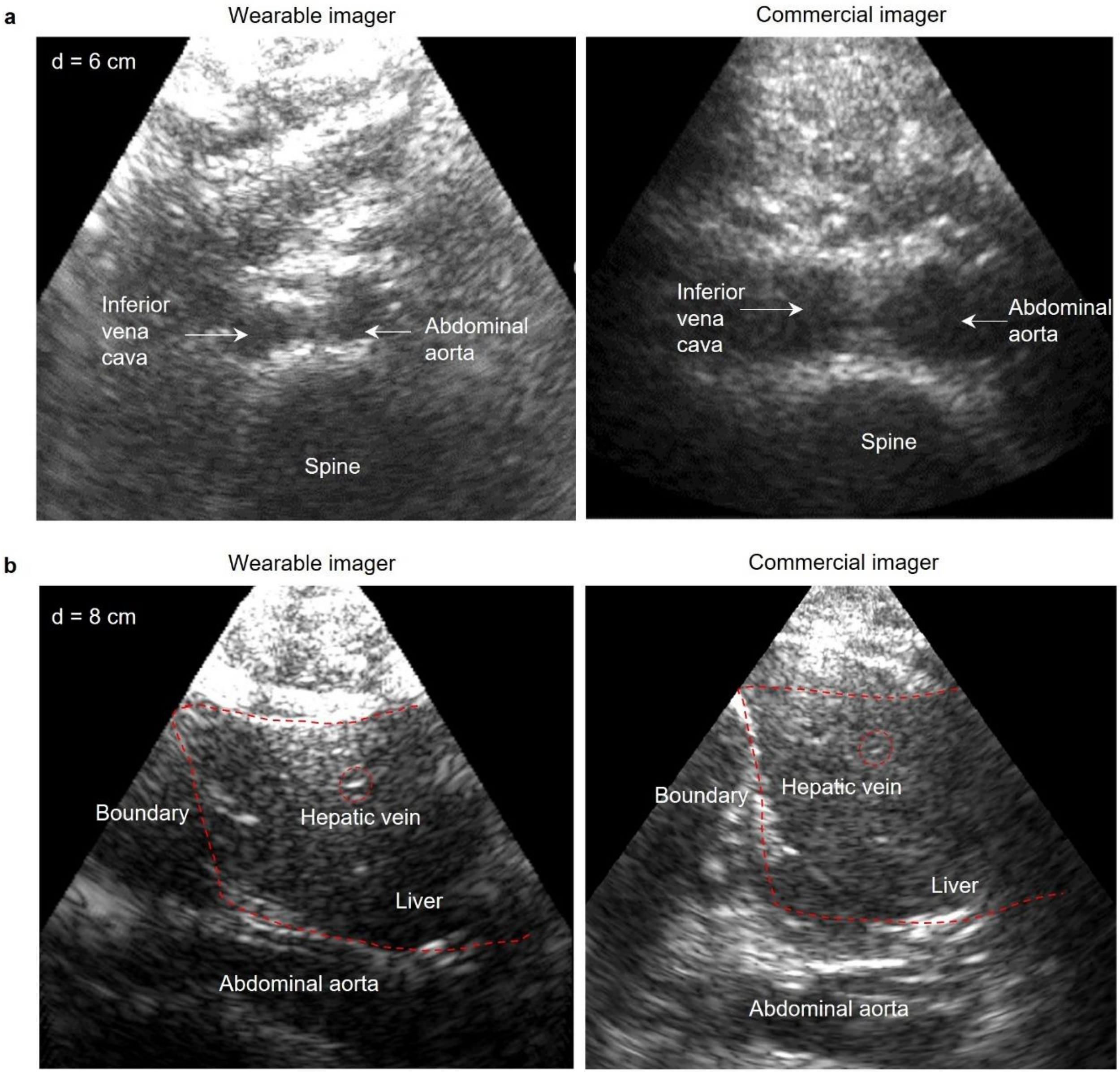


**Supplementary Fig. 30 | B-mode images of the abdominal area and liver from the wearable and the commercial imagers. a**, B-mode images of the abdominal area with a depth of ~6 cm. Similar structures including the inferior  vena cava and abdominal aorta can be recognized in both images. **b**, B-mode images of the liver with a depth of ~8 cm. The complete boundary and fine structures such as the hepatic vein can be observed in both images.

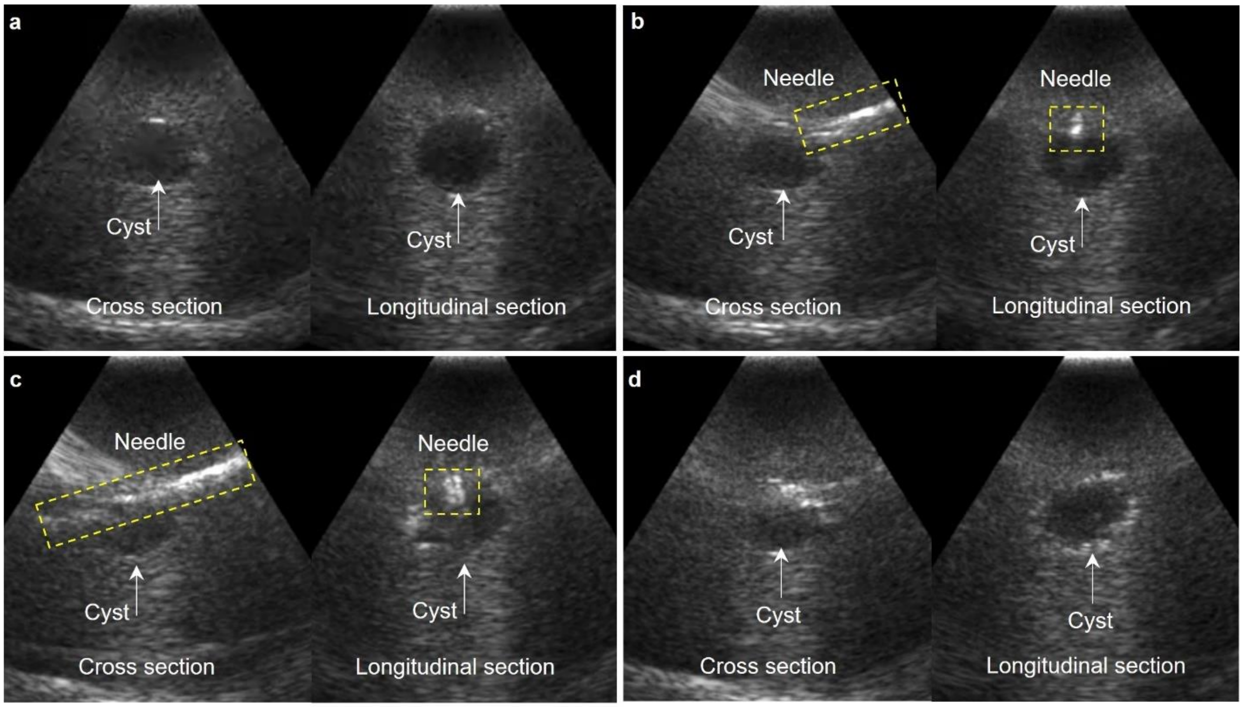


**Supplementary Fig. 31 | B-mode images of biopsy tests on a commercial phantom (CIRS 052).** **a**, The cross section and longitudinal section of the area of interest before inserting the biopsy needle. **b**, The cross section and longitudinal section of the area of interest after inserting the biopsy needle. **c**, The cross section and longitudinal section of the area of interest after releasing the inner stylet. **d**, The cross section and longitudinal section of the area of interest after removing the biopsy needle. The positions and the behaviors of the biopsy needle are clearly recorded in two orthogonal orientations simultaneously by the wearable imager. The uniquely enabling capability of the wearable imager is to forgo the need for an operator to constantly hold the device.

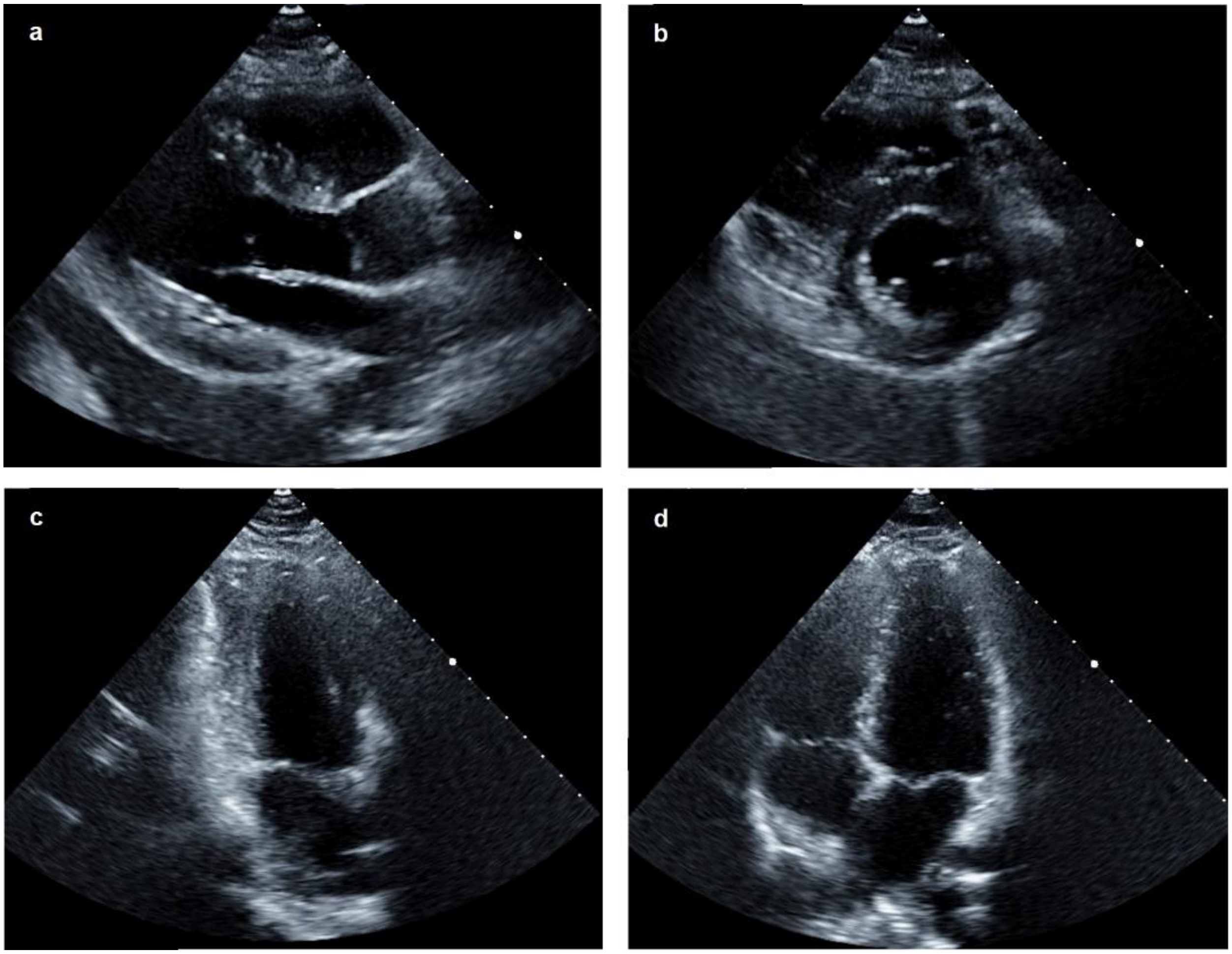


**Supplementary Fig. 32 | B-mode images of cardiac anatomies tested by an experienced cardiac sonographer.** B-mode images from **a**, parasternal long axis view, **b**, parasternal short axis view, **c**, apical two-chamber view, and d, apical four-chamber view using a clinical ultrasound machine.

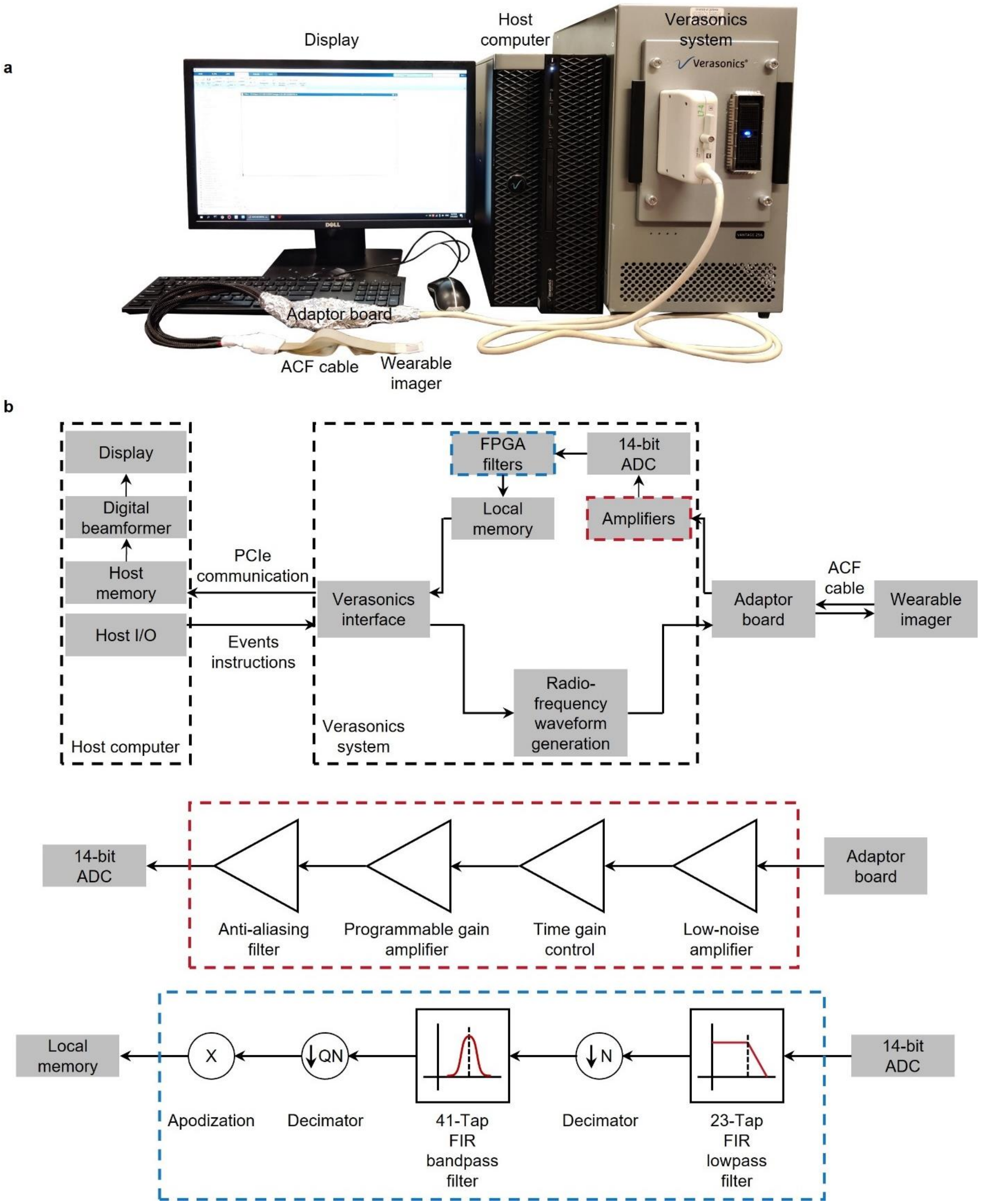


**Supplementary Fig. 33 | Photograph and schematics of the imaging system. a**, Experimental setup of the imaging system. Key components have been labelled. **b**, Working flow chart of the system. Red dashed box: Analog signal conditioning path. The low-noise amplifier pre-amplifies the raw signal with high fidelity to facilitate the following conditioning. The time gain control is a

programmable amplifier that can selectively amplify electrical signals induced by echoes from different depths. The intensity loss from deep regions is compensated in the time-gain control. The programmable gain amplifier allows the overall pixel level to be instantly adjustable when imaging. The anti-aliasing filter is a low-pass filter that cuts off the high-frequency component beyond the Nyquist frequency to make unambiguous analog-to-digital sampling. The corner frequency of the anti-aliasing filter is determined before each run according to the sampling rate. Blue dashed box: Digital signal conditioning path. The 23-tap FIR filter is programmed based on the transducer's center frequency and filters out signals with frequencies over four times of the central frequency. The decimator decimates the signals by N times, where N depends on the central frequency and sampling rate to lower the required bandwidth for data transmission. The 41-tap FIR bandpass filter only allows data near the center frequency to pass, which refines the signals. Another decimator downsamples the data stream according to the setting of the bandpass filter and sampling rate to maximize the transmission efficiency. Finally, apodization is applied to the data in each channel to fulfill the requirement for gradual aperture tapering. ACF: anisotropic conductive film; ADC: analog digital converter; FPGA: field-programmable gate array; PCIe: peripheral component interconnect express; FIR: finite impulse response; I/O: input/output.

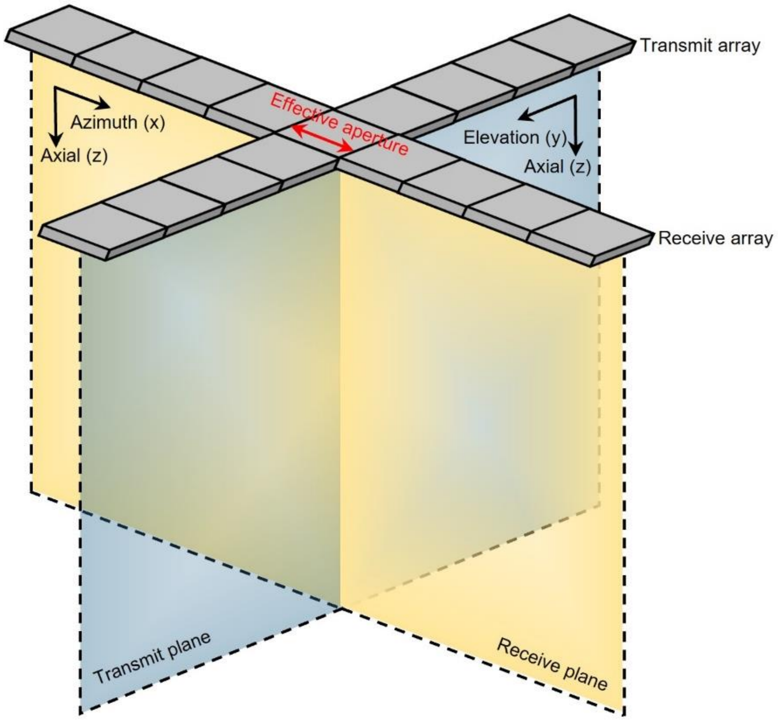


**Supplementary Fig. 34 | Configuration of a Mills cross array.** It includes a transmit array and a receive array. Transmit and receive beamforming applied by both arrays helps focus in the elevational direction. But the signal-to-noise ratio and the lateral resolution are limited in the reconstructed image due to the small effective aperture.

| Parameters / Modality | Spatial resolution | Temporal resolution | Radioactivity | Invasiveness | Mapping | Citation |
|---|---|---|---|---|---|---|
| Magnetic resonance imaging | 1.6 mm | 33.3 ms | No | No | 3D | [5] |
| X-Ray computed tomography | 0.3 mm | 50 ms | Yes | No | 3D | [6] |
| Single photon emission computed tomography | 10 mm | 37.5 ms | Yes | No | 3D | [7] |
| Positron emission tomography | 2 mm | 2000 ms | Yes | No | 3D | [7] |
| Optical voltage map | 1 mm | 0.25 ms | No | No | 2D | [8] |
| Optical coherence tomography | 0.001 mm | 85 ms | No | No | 3D | [9] |
| Ultrasonography | 1 mm | < 1 ms | No | No | 3D | [10,11] |

**Supplementary Table 1 | Summary of existing imaging methods for the heart.** Spatial resolution, temporal resolution, radioactivity, invasiveness, and mapping capability are evaluation parameters in this study. Comprehensive analysis of these parameters of cardiac imaging technologies helps us understand standards of medical imaging technologies, which serve as guidance for developing the wearable cardiac imager.

| **Model** | **Bonding strength (kPa)** | **Reference** |
|---|---|---|
| Histoacryl | 2 | [22] |
| Tisseel | 6 | [22] |
| Bioglue | 8 | [22] |
| 3M Electrically Conductive Adhesive Transfer Tape 9703 | 13 | [229] |
| Coseal | 18 | [22] |
| 3M Electrically Conductive Adhesive Transfer Tape 9712 | 20 | [230] |
| 3M Electrically Conductive Adhesive Transfer Tape 9713 | 22 | [231] |
| 3M Electrically Conductive Adhesive Transfer Tape 9719 | 27 | [232] |
| 3M EMI Copper Foil Shielding Tape 1181 | 27 | [233] |
| 3M 3313 Copper Foil Tape | 228 | [234] |
| Liquid metal electrode | 236 kPa | This work |
| Pure SEBS | 250 kPa | This work |

**Supplementary Table 2 | Comparison between the bonding strength of the liquid metal electrode, pure SEBS, and commercial adhesives.** The bonding strength between the electrode and the transducer element is stronger than many commercial adhesives, preventing the electrodes from delamination under various deformations.

| Model type | Training paradigm | Input Image Resolution | Learning rate | Optimizer | Code availability |
|---|---|---|---|---|---|
| **U-Net** | Supervised learning | 512 × 512 | $10^{-3}$ | Adam | [235] |
| **FCN-32** | Supervised learning | 512 × 512 | $10^{-3}$ | Adam | [236] |
| **FCN-8** | Supervised learning | 512 × 512 | $10^{-3}$ | Adam | [237] |
| **Attention-U-Net** | Supervised learning | 512 × 512 | $10^{-5}$ | Adam | [238] |
| **U-Net++** | Supervised learning | 512 × 512 | $10^{-4}$ | Adam | [239] |
| **FCN-8 VGG** | Supervised learning | 512 × 512 | $10^{-3}$ | Adam | [240] |
| **VGG-U-Net** | Supervised learning | 512 × 512 | $10^{-3}$ | Adam | [241] |
| **Resnet50-U-Net** | Supervised learning | 512 × 512 | $10^{-3}$ | Adam | [242] |

**Supplementary Table 3 | Model parameters and code availability.** Key parameters of all implemented models include model type, training paradigm, input image resolution, learning rate, optimizer, and code availability. The models' hyperparameters were carefully tuned and were implemented based on readable, consistent, and well-structured open source code.

| | Static, commercial probe | Static, wearable patch | After exercise, wearable patch (without data augmentation) | After exercise, wearable patch (with data augmentation) |
|---|---|---|---|---|
| **Size of labeled data** | 221 | 201 | 2029 | 10145 |
| **Size of unlabeled data** | 279 | 299 | 3829 | 3829 |

**Supplementary Table 4 | Sample sizes for all models.** All models were trained with all data collected in static and after intensive exercise.

| Model Name | Mean Intersection over Union (static) | Mean Intersection over Union (after exercise) |
|---|---|---|
| Attention-U-Net | 0.77 | 0.64 |
| U-Net++ | 0.78 | 0.65 |
| Nas-U-Net | 0.69 | 0.52 |
| Resnet50-U-Net | 0.91 | 0.84 |
| FCN-8 | 0.92 | 0.85 |
| FCN-8 VGG | 0.91 | 0.83 |
| U-Net | 0.85 | 0.74 |
| U-Net-mini | 0.16 | 0.09 |
| **FCN-32** | **0.93** | **0.87** |

**Supplementary Table 5 | Mean Intersection over Union among different models.** The table displays each model's mean Intersection over Unions on a testing dataset when the subject is static and after exercise. The evaluation of Intersection over Union is based on the comparison of a pair of predicted image and ground truth image. Specifically, the Intersection over Union equals to the number of pixels in the overlaid area of both images divided by the number of pixels in the combined area of both images. Mean Intersection over Union is the average Intersection over Union across all images in the testing dataset. The FCN-32 model has the highest mean Intersection over Union among all models.

**Supplementary Video 1. Cardiac long and short axis views imaged by an orthogonal array.**

**Supplementary Video 2. Cardiac apical four- and two- chamber views imaged by an orthogonal array.**

**Supplementary Video 3. Continuous cardiac imaging during rest, exercise, and recovery.**

**Supplementary Video 4. Left ventricle segmentation results by FCN-32.**

**Supplementary Video 5. Imaging guided biopsy on a phantom by an orthogonal array.**

## References


1 Levick, J. R. *An Introduction to Cardiovascular Physiology* (Butterworth-Heinemann, 1991).

2 Yazdanyar, A. & Newman, A. B. The burden of cardiovascular disease in the elderly: morbidity, mortality, and costs. *Clin. Geriatr. Med.* **25**, 563-577, vii (2009).

3 Ouyang, D. *et al.* Video-based AI for beat-to-beat assessment of cardiac function. *Nature* **580**, 252-256 (2020).

4 Jozwiak, M., Monnet, X. & Teboul, J. L. Monitoring: from cardiac output monitoring to echocardiography. *Curr. Opin. Crit. Care* **21**, 395-401 (2015).

5 Frahm, J., Voit, D. & Uecker, M. Real-Time Magnetic Resonance Imaging: Radial Gradient-Echo Sequences With Nonlinear Inverse Reconstruction. *Invest. Radiol.* **54**, 757-766 (2019).

6 Commandeur, F., Goeller, M. & Dey, D. Cardiac CT: Technological Advances in Hardware, Software, and Machine Learning Applications. *Curr. Cardiovasc. Imaging Rep* **11**, 1-12 (2018).

7 Angelidis, G. *et al.* SPECT and PET in ischemic heart failure. *Heart Fail. Rev.* **22**, 243-261 (2017).

8 Efimov, I. R., Nikolski, V. P. & Salama, G. Optical imaging of the heart. *Circ. Res.* **95**, 21-33 (2004).

9 Gargesha, M., Jenkins, M. W., Wilson, D. L. & Rollins, A. M. High temporal resolution OCT using image-based retrospective gating. *Opt. Express* **17**, 10786-10799 (2009).

10 Wang, R. Y. *et al.* High-resolution image reconstruction for portable ultrasound imaging devices. *Eurasip J. Adv. Sig. Pr.* **2019**, 1-12 (2019).

11 Baribeau, Y. *et al.* Handheld Point-of-Care Ultrasound Probes: The New Generation of POCUS. *J. Cardiothorac. Vasc. Anesth.* **34**, 3139-3145 (2020).

12 Zimetbaum, P. J. & Josephson, M. E. Use of the electrocardiogram in acute myocardial infarction. *N. Engl. J. Med.* **348**, 933-940 (2003).

13 Alihanka, J., Vaahtoranta, K. & Saarikivi, I. A new method for long-term monitoring of the ballistocardiogram, heart rate, and respiration. *Am. J. Physiol.* **240**, R384-392 (1981).

14 García-González, M. A., Argelagós-Palau, A., Fernández-Chimeno, M. & Ramos-Castro, J. in *Comput. Cardiol. 2013.* 461-464 (IEEE).

15 Elgendi, M. On the analysis of fingertip photoplethysmogram signals. *Curr. Cardiol. Rev.* **8**, 14-25 (2012).

16 Isaacson, D., Mueller, J. L., Newell, J. C. & Siltanen, S. Imaging cardiac activity by the D-bar method for electrical impedance tomography. *Physiol. Meas.* **27**, S43-50 (2006).

17 Schiller, N. B. *et al.* Recommendations for quantitation of the left ventricle by two-dimensional echocardiography. American Society of Echocardiography Committee on Standards, Subcommittee on Quantitation of Two-Dimensional Echocardiograms. *J. Am. Soc. Echocardiogr.* **2**, 358-367 (1989).

18 Hu, H. *et al.* Stretchable ultrasonic transducer arrays for three-dimensional imaging on complex surfaces. *Sci. Adv.* **4**, eaar3979 (2018).

19 Wang, C. *et al.* Monitoring of the central blood pressure waveform via a conformal ultrasonic device. *Nat. Biomed. Eng.* **2**, 687-695 (2018).

20 Shung, K. K. *Diagnostic Ultrasound* 1-232 (CRC press, Boca Raton, 2005).

21 Huang, Z. L. *et al.* Three-dimensional integrated stretchable electronics. *Nat. Electron.* **1**, 473-480 (2018).

22 Wu, S. J., Yuk, H., Wu, J., Nabzdyk, C. S. & Zhao, X. A Multifunctional Origami Patch for Minimally Invasive Tissue Sealing. *Adv. Mater.* **33**, e2007667 (2021).

23 Wu, H., Shen, G. & Chen, Y. A radiation emission shielding method for high intensity focus ultrasound probes. *Biomed. Mater. Eng.* **26 Suppl 1**, S959-966 (2015).

24 Chen, Q. P. *et al.* Ultrasonic inspection of curved structures with a hemispherical-omnidirectional ultrasonic probe via linear scan SAFT imaging. *Ndt. & E. International* **129** (2022).

25 Wang, C. *et al.* Bioadhesive ultrasound for long-term continuous imaging of diverse organs. *Science* **377**, 517-523 (2022).

26 Wang, C. *et al.* Continuous monitoring of deep-tissue haemodynamics with stretchable ultrasonic phased arrays. *Nat. Biomed. Eng.* **5**, 749-758 (2021).

27 Montaldo, G., Tanter, M., Bercoff, J., Benech, N. & Fink, M. Coherent plane-wave compounding for very high frame rate ultrasonography and transient elastography. *IEEE Trans Ultrason Ferroelectr Freq Control* **56**, 489-506 (2009).

28 Ghavami, M., Ilkhechi, A. K. & Zemp, R. Flexible transparent CMUT arrays for photoacoustic tomography. *Opt. Express* **30**, 15877-15894 (2022).

29 Xiao, Y., Boily, M., Hashemi, H. S. & Rivaz, H. High-Dynamic-Range Ultrasound: Application for Imaging Tendon Pathology. *Ultrasound Med. Biol.* **44**, 1525-1532 (2018).

30 Zander, D. *et al.* Ultrasound Image Optimization ("Knobology"): B-Mode. *Ultrasound Int Open* **6**, E14-E24 (2020).

31 Kempski, K. M., Graham, M. T., Gubbi, M. R., Palmer, T. & Lediju Bell, M. A. Application of the generalized contrast-to-noise ratio to assess photoacoustic image quality. *Biomed Opt Express* **11**, 3684-3698 (2020).

32 Huang, X., Lediju Bell, M. A. & Ding, K. Deep Learning for Ultrasound Beamforming in Flexible Array Transducer. *IEEE Trans. Med. Imaging* **40**, 3178-3189 (2021).

33 Cerqueira, M. D. *et al.* Standardized myocardial segmentation and nomenclature for tomographic imaging of the heart. A statement for healthcare professionals from the Cardiac Imaging Committee of the Council on Clinical Cardiology of the American Heart Association. *Circulation* **105**, 539-542 (2002).

34 Feigenbaum, H. Role of M-mode technique in today's echocardiography. *J. Am. Soc. Echocardiogr.* **23**, 240-257; 335-247 (2010).

35 Devereux, R. B. *et al.* Standardization of M-mode echocardiographic left ventricular

anatomic measurements. *J. Am. Coll. Cardiol.* **4**, 1222-1230 (1984).

36 Armstrong, W. F., Pellikka, P. A., Ryan, T., Crouse, L. & Zoghbi, W. A. Stress echocardiography: recommendations for performance and interpretation of stress echocardiography. Stress Echocardiography Task Force of the Nomenclature and Standards Committee of the American Society of Echocardiography. *J. Am. Soc. Echocardiogr.* **11**, 97-104 (1998).

37 Rerych, S. K., Scholz, P. M., Newman, G. E., Sabiston, D. C., Jr. & Jones, R. H. Cardiac function at rest and during exercise in normals and in patients with coronary heart disease: evaluation by radionuclide angiocardiography. *Ann. Surg.* **187**, 449-464 (1978).

38 Little, W. C. & Applegate, R. J. Congestive heart failure: systolic and diastolic function. *J. Cardiothorac. Vasc. Anesth.* **7**, 2-5 (1993).

39 Hill, J. & Timmis, A. Exercise tolerance testing. *BMJ* **324**, 1084-1087 (2002).

40 Marwick, T. H. Stress echocardiography. *Echocardiography* 491-519 (2018).

41 Hammermeister, K. E., Brooks, R. C. & Warbasse, J. R. The rate of change of left ventricular volume in man. I. Validation and peak systolic ejection rate in health and disease. *Circulation* **49**, 729-738 (1974).

42 Pellikka, P. A. *et al.* Variability in Ejection Fraction Measured By Echocardiography, Gated Single-Photon Emission Computed Tomography, and Cardiac Magnetic Resonance in Patients With Coronary Artery Disease and Left Ventricular Dysfunction. *JAMA Netw Open* **1**, e181456 (2018).

43 Ghorbanzadeh, O. *et al.* Evaluation of Different Machine Learning Methods and Deep-Learning Convolutional Neural Networks for Landslide Detection. *Remote Sensing* **11**, 196 (2019).

44 Bland, J. M. & Altman, D. G. Statistical Methods for Assessing Agreement between Two Methods of Clinical Measurement. *Lancet* **1**, 307-310 (1986).

45 Matheijssen, N. A. *et al.* Assessment of left ventricular volume and mass by cine magnetic resonance imaging in patients with anterior myocardial infarction intra-observer and inter-observer variability on contour detection. *Int. J. Card. Imaging* **12**, 11-19 (1996).

46 Fritzsche, R. G., Switzer, T. W., Hodgkinson, B. J. & Coyle, E. F. Stroke volume decline during prolonged exercise is influenced by the increase in heart rate. *J Appl Physiol* **86**, 799-805 (1999).

47 Pashaei, V. *et al.* Flexible Body-Conformal Ultrasound Patches for Image-Guided Neuromodulation. *IEEE Trans Biomed Circuits Syst* **14**, 305-318 (2020).

48 Kenny, J. S. *et al.* A novel, hands-free ultrasound patch for continuous monitoring of quantitative Doppler in the carotid artery. *Sci. Rep.* **11**, 7780 (2021).

49 Sung, F. *et al.* in *Proc. IEEE Comput. Soc. Conf. Comput. Vis. Pattern Recognit.* 1199-1208.

50 Kaelbling, L. P., Littman, M. L. & Moore, A. W. Reinforcement learning: A survey. *J. Artif. Intell. Res.* **4**, 237-285 (1996).

51 Lin, M. Y., Hu, H. J., Zhou, S. & Xu, S. Soft wearable devices for deep-tissue sensing. *Nat. Rev. Mater.*, 1-20 (2022).

52 Jeong, S. H. *et al.* Liquid alloy printing of microfluidic stretchable electronics. *Lab Chip* **12**, 4657-4664 (2012).

53 Kramer, R. K., Majidi, C. & Wood, R. J. Masked Deposition of Gallium-Indium Alloys for Liquid-Embedded Elastomer Conductors. *Adv. Funct. Mater.* **23**, 5292-5296 (2013).

54 Ladd, C., So, J. H., Muth, J. & Dickey, M. D. 3D printing of free standing liquid metal microstructures. *Adv. Mater.* **25**, 5081-5085 (2013).

55 Tabatabai, A., Fassler, A., Usiak, C. & Majidi, C. Liquid-phase gallium-indium alloy electronics with microcontact printing. *Langmuir* **29**, 6194-6200 (2013).

56 Cheng, S. & Wu, Z. Microfluidic electronics. *Lab Chip* **12**, 2782-2791 (2012).

57 Sempionatto, J. R. *et al.* An epidermal patch for the simultaneous monitoring of haemodynamic and metabolic biomarkers. *Nat. Biomed. Eng.* **5**, 737-748 (2021).

58 Liu, S., Shah, D. S. & Kramer-Bottiglio, R. Highly stretchable multilayer electronic circuits using biphasic gallium-indium. *Nat. Mater.* **20**, 851-858 (2021).

59 Ma, Z. *et al.* Permeable superelastic liquid-metal fibre mat enables biocompatible and monolithic stretchable electronics. *Nat. Mater.* **20**, 859-868 (2021).

60 Lopes, P. A., Santos, B. C., de Almeida, A. T. & Tavakoli, M. Reversible polymer-gel transition for ultra-stretchable chip-integrated circuits through self-soldering and self-coating and self-healing. *Nat. Commun.* **12**, 4666 (2021).

61 Mi, X. H., Qin, L., Liao, Q. W. & Wang, L. K. Electromechanical coupling coefficient and acoustic impedance of 1-1-3 piezoelectric composites. *Ceram. Int.* **43**, 7374-7377 (2017).

62 Wang, Z. *et al.* A flexible ultrasound transducer array with micro-machined bulk PZT. *Sensors (Basel)* **15**, 2538-2547 (2015).

63 Hong, C.-H. *et al.* Lead-free piezoceramics – Where to move on? *J. Materiomics* **2**, 1-24 (2016).

64 Zhu, B. P. *et al.* Sol-gel derived PMN-PT thick films for high frequency ultrasound linear array applications. *Ceram. Int.* **39**, 8709-8714 (2013).

65 Li, X. *et al.* 80-MHz intravascular ultrasound transducer using PMN-PT free-standing film. *IEEE Trans. Ultrason. Ferroelectr. Freq. Control* **58**, 2281-2288 (2011).

66 Zhu, B. *et al.* Lift-off PMN-PT Thick Film for High Frequency Ultrasonic Biomicroscopy. *J. Am. Ceram. Soc.* **93**, 2929-2931 (2010).

67 Shahriari, S. & Garcia, D. Meshfree simulations of ultrasound vector flow imaging using smoothed particle hydrodynamics. *Phys. Med. Biol.* **63**, 205011 (2018).

68 Sun, X., Li, Y. & Liu, H. in *2017 8th International IEEE/EMBS Conference on NER* 122-125 (IEEE).

69 AlMohimeed, I., Turkistani, H. & Ono, Y. in *2013 IEEE Int. Ultrason. Symp.* 1137-1140 (IEEE).

70 Bowen, C. R., Bradley, L. R., Almond, D. P. & Wilcox, P. D. Flexible piezoelectric

transducer for ultrasonic inspection of non-planar components. *Ultrasonics* **48**, 367-375 (2008).

71 Farus, L. Wearable ultrasound array for point-of-care imaging and patient monitoring. *Medicine Meets Virtual Reality 22: NextMed/MMVR22* **220**, 241 (2016).

72 Bhuyan, A. *et al.* in *2011 IEEE Int. Ultrason. Symp.* 1060-1063 (IEEE).

73 Roy, K. *et al.* in *2020 IEEE Int. Ultrason. Symp.* 1-4 (IEEE).

74 Kato, Y. *et al.* Large-Area Flexible Ultrasonic Imaging System With an Organic Transistor Active Matrix. *T Electron Dev* **57**, 995-1002 (2010).

75 Peng, C., Chen, M., Sim, H. K., Zhu, Y. & Jiang, X. in *2020 IEEE 15th International Conference on NEMS,* 143-146 (IEEE).

76 Wang, F. *et al.* Flexible Doppler ultrasound device for the monitoring of blood flow velocity. *Sci. Adv.* **7**, eabi9283 (2021).

77 Chen, J., Liu, W., Wu, D. & Ye, H. Laser Micromachined Flexible Ultrasound Line Array and Subplanar Multimodal Imaging Applications. *IEEE Open Journal of Trans. Ultrason. Ferroelectr. Freq. Control* **2**, 131-139 (2022).

78 Safavi, K. C. *et al.* Variation exists in rates of admission to intensive care units for heart failure patients across hospitals in the United States. *Circulation* **127**, 923-929 (2013).

79 Dar, O. & Cowie, M. R. Acute heart failure in the intensive care unit: epidemiology. *Crit. Care Med.* **36**, S3-8 (2008).

80 de Mendonca, A. *et al.* Acute renal failure in the ICU: risk factors and outcome evaluated by the SOFA score. *Intensive Care Med.* **26**, 915-921 (2000).

81 Yildiz, M. *et al.* Left ventricular hypertrophy and hypertension. *Prog. Cardiovasc. Dis.* **63**, 10-21 (2020).

82 Bhella, P. S. *et al.* Impact of lifelong exercise "dose" on left ventricular compliance and distensibility. *J. Am. Coll. Cardiol.* **64**, 1257-1266 (2014).

83 Moss, R. L. & Fitzsimons, D. P. **90** 11-13 (Am Heart Assoc, 2002).

84 Allan, P. L. *Clinical Doppler Ultrasound* (Elsevier Health Sciences, 2006).

85 Gennisson, J. L., Deffieux, T., Fink, M. & Tanter, M. Ultrasound elastography: principles and techniques. *Diagn Interv Imaging* **94**, 487-495 (2013).

86 Soepriatna, A. H., Damen, F. W., Vlachos, P. P. & Goergen, C. J. Cardiac and respiratory-gated volumetric murine ultrasound. *Int. J. Cardiovasc. Imaging* **34**, 713-724 (2018).

87 Bercoff, J. Ultrafast ultrasound imaging. *Ultrasound Medical*, 3-24 (2011).

88 Christensen-Jeffries, K. *et al.* Super-resolution Ultrasound Imaging. *Ultrasound Med. Biol.* **46**, 865-891 (2020).

89 Cotero, V. *et al.* Noninvasive sub-organ ultrasound stimulation for targeted neuromodulation. *Nat. Commun.* **10**, 952 (2019).

90 Woudenberg, N. V. *et al.* in *Simulation, image processing, and ultrasound systems for assisted diagnosis and navigation* 74-81 (Springer, 2018).

91 Rothberg, J. M. *et al.* Ultrasound-on-chip platform for medical imaging, analysis, and

collective intelligence. *Proc. Natl. Acad. Sci. U. S. A.* **118**, e2019339118 (2021).
92 Hou, C. *et al.* Optimized Backing Layers Design for High Frequency Broad Bandwidth Ultrasonic Transducer. *IEEE Trans. Biomed. Eng.* **69**, 475-481 (2022).
93 Kim, H. *et al.* High-Attenuation Backing Layer for Miniaturized Ultrasound Imaging Transducer. *IEEE Trans. Ultrason. Ferroelectr. Freq. Control* **69**, 1960-1969 (2022).
94 Connors, A. F. The Effectiveness of Right Heart Catheterization in the Initial Care of Critically III Patients. *JAMA:* **276**, 889-897 (1996).
95 Darmon, P. L., Hillel, Z., Mogtader, A., Mindich, B. & Thys, D. Cardiac output by transesophageal echocardiography using continuous-wave Doppler across the aortic valve. *Anesthesiology* **80**, 796-805; discussion 725A (1994).
96 Kanaya, N., Hirata, N., Kurosawa, S., Nakayama, M. & Namiki, A. Differential effects of propofol and sevoflurane on heart rate variability. *Anesthesiology* **98**, 34-40 (2003).
97 Enriquez, A. *et al.* Use of Intracardiac Echocardiography in Interventional Cardiology: Working With the Anatomy Rather Than Fighting It. *Circulation* **137**, 2278-2294 (2018).
98 Angelone, A. & Coulter, N. A., Jr. Respiratory Sinus Arrhythemia: A Frequency Dependent Phenomenon. *J. Appl. Physiol.* **19**, 479-482 (1964).
99 Nagga, K., Dong, H. J., Marcusson, J., Skoglund, S. O. & Wressle, E. Health-related factors associated with hospitalization for old people: comparisons of elderly aged 85 in a population cohort study. *Arch. Gerontol. Geriatr.* **54**, 391-397 (2012).
100 Serdyuk, S. *et al.* Cardiac arrhythmias and sudden unexpected death in epilepsy: Results of long-term monitoring. *Heart Rhythm* **18**, 221-228 (2021).
101 Jelinek, M. V. & Lown, B. Exercise stress testing for exposure of cardiac arrhythmia. *Prog. Cardiovasc. Dis.* **16**, 497-522 (1974).
102 Vourvouri, E. C., Poldermans, D., Deckers, J. W., Parharidis, G. E. & Roelandt, J. R. Evaluation of a hand carried cardiac ultrasound device in an outpatient cardiology clinic. *Heart* **91**, 171-176 (2005).
103 Nemati, E., Deen, M. J. & Mondal, T. A Wireless Wearable ECG Sensor for Long-Term Applications. *IEEE Commun. Mag.* **50**, 36-43 (2012).
104 Paradkar, N. & Chowdhury, S. R. in *2017 39th Annual International Conference of the IEEE EMBC,* 113-116 (IEEE).
105 Solosenko, A., Petrenas, A., Marozas, V. & Sornmo, L. Modeling of the photoplethysmogram during atrial fibrillation. *Comput. Biol. Med.* **81**, 130-138 (2017).
106 Lee, G., Sanders, P. & Kalman, J. M. Catheter ablation of atrial arrhythmias: state of the art. *Lancet* **380**, 1509-1519 (2012).
107 Lip, G. Y. & Hee, F. L. Paroxysmal atrial fibrillation. *QJM* **94**, 665-678 (2001).
108 Kerr, C. R. *et al.* Progression to chronic atrial fibrillation after the initial diagnosis of paroxysmal atrial fibrillation: results from the Canadian Registry of Atrial Fibrillation. *Am. Heart J.* **149**, 489-496 (2005).
109 Schor, S. S., Elsom, K. A., Elsom, K. O. & Dunn, J. P. An Evaluation of the Periodic Health

Examination: A Study of Factors Discriminating between Survival and Death from Coronary Heart Disease. *Ann. Intern. Med.* **61**, 1006-1014 (1964).

110 Thygesen, K., Alpert, J. S., White, H. D. & Joint, E. S. C. A. A. H. A. W. H. F. T. F. f. t. R. o. M. I. Universal definition of myocardial infarction. *J. Am. Coll. Cardiol.* **50**, 2173-2195 (2007).

111 Xu, S. *et al.* Soft microfluidic assemblies of sensors, circuits, and radios for the skin. *Science* **344**, 70-74 (2014).

112 Alruwaili, F., Cluff, K., Griffith, J. & Farhoud, H. Passive Self Resonant Skin Patch Sensor to Monitor Cardiac Intraventricular Stroke Volume Using Electromagnetic Properties of Blood. *IEEE J. Transl. Eng. Health Med.* **6**, 1900709 (2018).

113 Dagdeviren, C. *et al.* Conformal piezoelectric energy harvesting and storage from motions of the heart, lung, and diaphragm. *Proc. Natl. Acad. Sci. U. S. A.* **111**, 1927-1932 (2014).

114 Van den Oever, H. L., Murphy, E. J. & Christie-Taylor, G. A. USCOM (Ultrasonic Cardiac Output Monitors) lacks agreement with thermodilution cardiac output and transoesophageal echocardiography valve measurements. *Anaesth. Intensive Care* **35**, 903-910 (2007).

115 Edler, I. & Lindstrom, K. The history of echocardiography. *Ultrasound Med. Biol.* **30**, 1565-1644 (2004).

116 Karamitsos, T. D., Francis, J. M., Myerson, S., Selvanayagam, J. B. & Neubauer, S. The role of cardiovascular magnetic resonance imaging in heart failure. *J. Am. Coll. Cardiol.* **54**, 1407-1424 (2009).

117 Goo, H. W. *et al.* Computed tomography for the diagnosis of congenital heart disease in pediatric and adult patients. *Int. J. Cardiovasc. Imaging* **21**, 347-365; discussion 367 (2005).

118 Greenwood, J. P. *et al.* Cardiovascular magnetic resonance and single-photon emission computed tomography for diagnosis of coronary heart disease (CE-MARC): a prospective trial. *Lancet* **379**, 453-460 (2012).

119 Machac, J. in *Seminars in nuclear medicine* 17-36 (Elsevier, 2005).

120 Jenkins, M. W., Watanabe, M. & Rollins, A. M. Longitudinal Imaging of Heart Development With Optical Coherence Tomography. *IEEE J. Sel. Top. Quantum Electron.* **18**, 1166-1175 (2012).

121 Coote, J. H. Recovery of heart rate following intense dynamic exercise. *Exp. Physiol.* **95**, 431-440 (2010).

122 Simonson, E. *et al.* Cardiovascular Stress (Electrocardiographic Changes) Produced by Driving an Automobile. *Am. Heart J.* **75**, 125 (1968).

123 Sutherland, G. R. *et al.* Quantitation of left-ventricular asynergy by cardiac ultrasound. *Am. J. Cardiol.* **86**, 4G-9G (2000).

124 Berger, A. Magnetic resonance imaging. *BMJ* **324**, 35 (2002).

125 Hamilton, J., Franson, D. & Seiberlich, N. Recent advances in parallel imaging for MRI. *Prog. Nucl. Magn. Reson. Spectrosc.* **101**, 71-95 (2017).

126 Garvey, C. J. & Hanlon, R. Computed tomography in clinical practice. *BMJ* **324**, 1077-1080 (2002).

127 Kalisz, K. *et al.* Artifacts at Cardiac CT: Physics and Solutions. *Radiographics* **36**, 2064-2083 (2016).

128 Khalil, M. M., Tremoleda, J. L., Bayomy, T. B. & Gsell, W. Molecular SPECT Imaging: An Overview. *Int J Mol Imaging* **2011**, 796025 (2011).

129 Cherry, S. R. & Dahlbom, M. *PET: Physics, Instrumentation, and Scanners,* 1-117 (Springer, 2006).

130 Song, T. A., Chowdhury, S. R., Yang, F. & Dutta, J. Super-Resolution PET Imaging Using Convolutional Neural Networks. *IEEE Trans Comput Imaging* **6**, 518-528 (2020).

131 Guobao, W. High Temporal-Resolution Dynamic PET Image Reconstruction Using a New Spatiotemporal Kernel Method. *IEEE Trans. Med. Imaging* **38**, 664-674 (2019).

132 Chen, C. C., Shen, T. Y., Peterson, C. B., Hung, G. U. & Pan, T. Comparison of ejection fraction calculation between CT and SPECT at high heart rate: A dynamic cardiac phantom study. *J. Nucl. Cardiol.* **28**, 311-316 (2021).

133 Newman, P. G. & Rozycki, G. S. The history of ultrasound. *Surg. Clin. North Am.* **78**, 179-195 (1998).

134 Carovac, A., Smajlovic, F. & Junuzovic, D. Application of ultrasound in medicine. *Acta Inform. Med.* **19**, 168-171 (2011).

135 Hasegawa, H. in *AIP Conf. Proc.* 020015 (AIP Publishing LLC).

136 Williams, D. The physics of ultrasound. *Intensive Care* **13**, 264-268 (2012).

137 Viessmann, O. M., Eckersley, R. J., Christensen-Jeffries, K., Tang, M. X. & Dunsby, C. Acoustic super-resolution with ultrasound and microbubbles. *Phys. Med. Biol.* **58**, 6447-6458 (2013).

138 Joy, J., Cooke, I. & Love, M. Is ultrasound safe? *The Obstetrician & Gynaecologist* **8**, 222-227 (2006).

139 FDA, U. Marketing Clearance of diagnostic ultrasound systems and transducers—guidance for Industry and Food and Drug Administration Staff. *Rockville, MD: FDA* (2019).

140 Stewart, P. A., Tonge, H. M. & Wladimiroff, J. W. Arrhythmia and structural abnormalities of the fetal heart. *Br. Heart J.* **50**, 550-554 (1983).

141 Dantas, R. G., Costa, E. T. & Leeman, S. Ultrasound speckle and equivalent scatterers. *Ultrasonics* **43**, 405-420 (2005).

142 Lanata, A., Scilingo, E. P., Francesconi, R., Varone, G. & De Rossi, D. in *2006 IEEE Sensors,* 489-492 (IEEE).

143 Smith, S. W., Pavy, H. R. & von Ramm, O. T. High-speed ultrasound volumetric imaging system. I. Transducer design and beam steering. *IEEE Trans. Ultrason. Ferroelectr. Freq. Control* **38**, 100-108 (1991).

144 Yen, J. T. & Smith, S. W. Real-time rectilinear volumetric imaging. *IEEE Trans. Ultrason.*

*Ferroelectr. Freq. Control* **49**, 114-124 (2002).

145 Demore, C. E. M., Joyce, A. W., Wall, K. & Lockwood, G. R. Real-Time Volume Imaging Using a Crossed Electrode Array. *Control* **56**, 1252-1261 (2009).

146 Yen, J. T. Beamforming of sound from two-dimensional arrays using spatial matched filters. *J. Acoust. Soc. Am.* **134**, 3697-3704 (2013).

147 Kim, K.-S. & Song, T.-K. in *IEEE Ultrasonics Symposium, 2004.* 1409-1412 (IEEE).

148 Fernandez, A. T. *et al.* Synthetic elevation beamforming and image acquisition capabilities using an 8/spl times/128 1.75 D array. *IEEE Trans. Ultrason. Ferroelectr. Freq. Control* **50**, 40-57 (2003).

149 Yan, S., Guo, P. & Zhu, Q. in *Proceedings of the IEEE 27th* 61-62 (IEEE).

150 Dahl, J. J., McAleavey, S. A., Pinton, G. F., Soo, M. S. & Trahey, G. E. Adaptive imaging on a diagnostic ultrasound scanner at quasi real-time rates. *IEEE Trans. Ultrason. Ferroelectr. Freq. Control* **53**, 1832-1843 (2006).

151 Ferree, T. C., Luu, P., Russell, G. S. & Tucker, D. M. Scalp electrode impedance, infection risk, and EEG data quality. *Clin. Neurophysiol.* **112**, 536-544 (2001).

152 Sánchez, C. C., Glover, P., Power, H. & Bowtell, R. Calculation of the electric field resulting from human body rotation in a magnetic field. *Phys. Med. Biol.* **57**, 4739 (2012).

153 Stuchly, M. A. & Zhao, S. K. Magnetic field-induced currents in the human body in proximity of power lines. *IEEE Trans. Power Delivery* **11**, 102-109 (1996).

154 Mitchell, C. *et al.* Guidelines for Performing a Comprehensive Transthoracic Echocardiographic Examination in Adults: Recommendations from the American Society of Echocardiography. *J. Am. Soc. Echocardiogr.* **32**, 1-64 (2019).

155 Uribarri, A., Bueno, H., Yotti, R. & Perez-David, E. Acute heart failure as presentation of left-ACAOS. *Eur. Heart J.* **34**, 2787 (2013).

156 Austeng, A., Nilsen, C.-I. C., Jensen, A. C., Näsholm, S. P. & Holm, S. in *2011 IEEE International Ultrasonics Symposium* 2448-2451 (IEEE).

157 Stanziola, A. *et al.* Motion Artifacts and Correction in Multipulse High-Frame Rate Contrast-Enhanced Ultrasound. *IEEE Trans. Ultrason. Ferroelectr. Freq. Control* **66**, 417-420 (2019).

158 Prabhu, S. J., Kanal, K., Bhargava, P., Vaidya, S. & Dighe, M. K. Ultrasound artifacts: classification, applied physics with illustrations, and imaging appearances. *Ultrasound Q* **30**, 145-157 (2014).

159 Kallel, F., Bertrand, M. & Meunier, J. Speckle Motion Artifact under Tissue Rotation. *Control* **41**, 105-122 (1994).

160 Kirberger, R. M. Imaging Artifacts in Diagnostic Ultrasound - a Review. *Vet. Radiol. Ultrasound* **36**, 297-306 (1995).

161 Cattermole, G. N. *et al.* The normal ranges of cardiovascular parameters measured using the ultrasonic cardiac output monitor. *Physiol Rep* **5**, e13195 (2017).

162 Nagel, E. *et al.* Noninvasive diagnosis of ischemia-induced wall motion abnormalities with

the use of high-dose dobutamine stress MRI: comparison with dobutamine stress echocardiography. *Circulation* **99**, 763-770 (1999).

163 Picano, E., Pibarot, P., Lancellotti, P., Monin, J. L. & Bonow, R. O. The emerging role of exercise testing and stress echocardiography in valvular heart disease. *J. Am. Coll. Cardiol.* **54**, 2251-2260 (2009).

164 Argiento, P. *et al.* Exercise stress echocardiography for the study of the pulmonary circulation. *Eur. Respir. J.* **35**, 1273-1278 (2010).

165 Lang, R. M. *et al.* Recommendations for cardiac chamber quantification by echocardiography in adults: an update from the American Society of Echocardiography and the European Association of Cardiovascular Imaging. *J. Am. Soc. Echocardiogr.* **28**, 1-39 e14 (2015).

166 Koh, A. S. *et al.* A comprehensive population-based characterization of heart failure with mid-range ejection fraction. *Eur. J. Heart Fail.* **19**, 1624-1634 (2017).

167 Kim, Y. S., Park, M. J., Rhim, H., Lee, M. W. & Lim, H. K. Sonographic analysis of the intercostal spaces for the application of high-intensity focused ultrasound therapy to the liver. *AJR Am. J. Roentgenol.* **203**, 201-208 (2014).

168 Turakhia, M. P., McManus, D. D., Whooley, M. A. & Schiller, N. B. Increase in end-systolic volume after exercise independently predicts mortality in patients with coronary heart disease: data from the Heart and Soul Study. *Eur. Heart J.* **30**, 2478-2484 (2009).

169 Jordan, J. H. *et al.* Early Myocardial Strain Changes During Potentially Cardiotoxic Chemotherapy May Occur as a Result of Reductions in Left Ventricular End-Diastolic Volume: The Need to Interpret Left Ventricular Strain With Volumes. *Circulation* **135**, 2575-2577 (2017).

170 Lock, J. E., Block, P. C., McKay, R. G., Baim, D. S. & Keane, J. F. Transcatheter closure of ventricular septal defects. *Circulation* **78**, 361-368 (1988).

171 Stout, K. K. & Verrier, E. D. Acute valvular regurgitation. *Circulation* **119**, 3232-3241 (2009).

172 Cafarelli, A., Miloro, P., Verbeni, A., Carbone, M. & Menciassi, A. Speed of sound in rubber-based materials for ultrasonic phantoms. *J. Ultrasound* **19**, 251-256 (2016).

173 Waters, K. R., Hughes, M. S., Mobley, J., Brandenburger, G. H. & Miller, J. G. On the applicability of Kramers-Kronig relations for ultrasonic attenuation obeying a frequency power law. *J. Acoust. Soc. Am.* **108**, 556-563 (2000).

174 http://www.factor2.com/v/vspfiles/msds_2015/a-4717tech%20F2.pdf

175 McDonagh, T. A. *et al.* Corrigendum to: 2021 ESC Guidelines for the diagnosis and treatment of acute and chronic heart failure: Developed by the Task Force for the diagnosis and treatment of acute and chronic heart failure of the European Society of Cardiology (ESC) With the special contribution of the Heart Failure Association (HFA) of the ESC. *Eur. Heart J.* **42**, 4901 (2021).

176 Konstam, M. A. & Abboud, F. M. Ejection Fraction: Misunderstood and Overrated

(Changing the Paradigm in Categorizing Heart Failure). *Circulation* **135**, 717-719 (2017).
177 Bosch, X. & Theroux, P. Left ventricular ejection fraction to predict early mortality in patients with non-ST-segment elevation acute coronary syndromes. *Am. Heart J.* **150**, 215-220 (2005).
178 Curtis, J. P. *et al.* The association of left ventricular ejection fraction, mortality, and cause of death in stable outpatients with heart failure. *J. Am. Coll. Cardiol.* **42**, 736-742 (2003).
179 Vincent, J. L. Understanding cardiac output. *Crit. Care* **12**, 174 (2008).
180 Ehlers, K. C., Mylrea, K. C., Waterson, C. K. & Calkins, J. M. Cardiac output measurements. A review of current techniques and research. *Ann. Biomed. Eng.* **14**, 219-239 (1986).
181 Jakovljevic, D. G., Trenell, M. I. & MacGowan, G. A. Bioimpedance and bioreactance methods for monitoring cardiac output. *Best Pract. Res. Clin. Anaesthesiol.* **28**, 381-394 (2014).
182 Marik, P. E. Noninvasive cardiac output monitors: a state-of the-art review. *J. Cardiothorac. Vasc. Anesth.* **27**, 121-134 (2013).
183 Juncos, L. I. & Juncos, L. A. in *Clinical Decisions in Nephrology, Hypertension and Kidney Transplantation* Chapter 18, 175-182 (Springer, 2013).
184 Kakihana, Y., Ito, T., Nakahara, M., Yamaguchi, K. & Yasuda, T. Sepsis-induced myocardial dysfunction: pathophysiology and management. *J Intensive Care* **4**, 22 (2016).
185 Degroot, W. J. & Leonard, J. J. Hyperthyroidism as a High Cardiac Output State. *Am. Heart J.* **79**, 265-& (1970).
186 Fowler, N. O. & Holmes, J. C. Blood viscosity and cardiac output in acute experimental anemia. *J. Appl. Physiol.* **39**, 453-456 (1975).
187 Bamira, D. & Picard, M. in *Encyclopedia of Cardiovascular Research and Medicine* 35-45 (Elsevier, 2018).
188 Devereux, R. B. *et al.* Congestive heart failure despite normal left ventricular systolic function in a population-based sample: the Strong Heart Study. *Am. J. Cardiol.* **86**, 1090-1096 (2000).
189 Villari, B. *et al.* Influence of collagen network on left ventricular systolic and diastolic function in aortic valve disease. *J. Am. Coll. Cardiol.* **22**, 1477-1484 (1993).
190 Borlaug, B. A. & Redfield, M. M. Diastolic and systolic heart failure are distinct phenotypes within the heart failure spectrum. *Circulation* **123**, 2006-2013; discussion 2014 (2011).
191 Little, R. C. & Little, W. C. Cardiac preload, afterload, and heart failure. *Arch. Intern. Med.* **142**, 819-822 (1982).
192 Critchley, L. A., Lee, A. & Ho, A. M. A critical review of the ability of continuous cardiac output monitors to measure trends in cardiac output. *Anesth. Analg.* **111**, 1180-1192 (2010).
193 Wesseling, K. H., Jansen, J. R., Settels, J. J. & Schreuder, J. J. Computation of aortic flow from pressure in humans using a nonlinear, three-element model. *J. Appl. Physiol. (1985)*

**74**, 2566-2573 (1993).

194 Reuter, D. A. *et al.* Usefulness of left ventricular stroke volume variation to assess fluid responsiveness in patients with reduced cardiac function. *Crit. Care Med.* **31**, 1399-1404 (2003).

195 Jhanji, S., Dawson, J. & Pearse, R. M. Cardiac output monitoring: basic science and clinical application. *Anaesthesia* **63**, 172-181 (2008).

196 Piculjan, A., Sustic, M., Brumini, G., Kuharic, J. & Sustic, A. Reliability of B-line quantification by different-level observers and a software algorithm using point-of-care lung ultrasound. *J. Clin. Monit. Comput.* **34**, 1259-1264 (2020).

197 Huang, Q., Lan, J. & Li, X. Robotic Arm Based Automatic Ultrasound Scanning for Three-Dimensional Imaging. *IEEE Trans. Ind. Inf.* **15**, 1173-1182 (2019).

198 Argueta, E. E. & Paniagua, D. Thermodilution Cardiac Output: A Concept Over 250 Years in the Making. *Cardiol. Rev.* **27**, 138-144 (2019).

199 Bottiger, B. W. *et al.* Continuous versus intermittent thermodilution cardiac output measurement during orthotopic liver transplantation. *Anaesthesia* **52**, 207-214 (1997).

200 Norris, S. L., King, E. G., Grace, M. & Weir, B. Thermodilution cardiac output--an in vitro model of low flow states. *Crit. Care Med.* **14**, 57-59 (1986).

201 Baan, J. *et al.* Continuous stroke volume and cardiac output from intra-ventricular dimensions obtained with impedance catheter. *Cardiovasc. Res.* **15**, 328-334 (1981).

202 Rumberger, J. A. *et al.* in *Mayo Clin. Proc.* 860-870 (Elsevier, 1999).

203 Timmins, A. C., Giles, M., Nathan, A. W. & Hinds, C. J. Clinical validation of a radionuclide detector to measure ejection fraction in critically ill patients. *Br. J. Anaesth.* **72**, 523-528 (1994).

204 Swamy, G., Kuiper, J., Gudur, M. S., Olivier, N. B. & Mukkamala, R. Continuous left ventricular ejection fraction monitoring by aortic pressure waveform analysis. *Ann. Biomed. Eng.* **37**, 1055-1068 (2009).

205 Critchley, L. A. & Critchley, J. A. A meta-analysis of studies using bias and precision statistics to compare cardiac output measurement techniques. *J. Clin. Monit. Comput.* **15**, 85-91 (1999).

206 Lester, S. J., Ryan, E. W., Schiller, N. B. & Foster, E. Best method in clinical practice and in research studies to determine left atrial size. *Am. J. Cardiol.* **84**, 829-832 (1999).

207 Giustiniano, E., Padua, E., Negri, K., Bragato, R. M. & Cecconi, M. Echocardiography during Prone-Position Mechanical Ventilation in Patients with COVID-19: A Proposal for a New Approach. *J. Am. Soc. Echocardiogr.* **33**, 905-906 (2020).

208 Harris, M. & Chung, F. Complications of general anesthesia. *Clin. Plast. Surg.* **40**, 503-513 (2013).

209 Devereaux, P. J. *et al.* Characteristics and short-term prognosis of perioperative myocardial infarction in patients undergoing noncardiac surgery: a cohort study. *Ann. Intern. Med.* **154**, 523-528 (2011).

210 Priebe, H. J. Preoperative cardiac management of the patient for non-cardiac surgery: an individualized and evidence-based approach. *Br. J. Anaesth.* **107**, 83-96 (2011).

211 McLeod, G. *et al.* Echocardiography in Congenital Heart Disease. *Prog. Cardiovasc. Dis.* **61**, 468-475 (2018).

212 Smith, C. D., Weber, C. J. & Amerson, J. R. Laparoscopic adrenalectomy: new gold standard. *World J. Surg.* **23**, 389-396 (1999).

213 Ronneberger, O., Fischer, P. & Brox, T. in *Medical Image Computing and Computer-Assisted Intervention – MICCAI 2015,* 234-241 (Springer, 2015).

214 Long, J., Shelhamer, E. & Darrell, T. in *Proceedings of the IEEE conference on CVPR* 3431-3440 (2015).

215 Oktay, O. *et al.* Attention u-net: Learning where to look for the pancreas. *arXiv preprint arXiv:1804.03999* (2018).

216 Zhou, Z., Rahman Siddiquee, M. M., Tajbakhsh, N. & Liang, J. in *Deep Learning in Medical Image Analysis and Multimodal Learning for Clinical Decision Support* 3-11 (Springer, 2018).

217 Weng, Y., Zhou, T. B., Li, Y. J. & Qiu, X. Y. NAS-Unet: Neural Architecture Search for Medical Image Segmentation. *IEEE Access* **7**, 44247-44257 (2019).

218 Al-Haija, Q. A. & Adebanjo, A. in *2020 IEEE International IOT, IEMTRONICS* 1-7 (IEEE).

219 Mao, Y. X. *et al.* Efficient Low-Cost Ship Detection for SAR Imagery Based on Simplified U-Net. *IEEE Access* **8**, 69742-69753 (2020).

220 Wahr, D. W., Wang, Y. S. & Schiller, N. B. Left ventricular volumes determined by two-dimensional echocardiography in a normal adult population. *J. Am. Coll. Cardiol.* **1**, 863-868 (1983).

221 Rezatofighi, H. *et al.* in *Proceedings of the IEEE/CVF conference on CVPR* 658-666 (2019).

222 Smistad, E. & Østvik, A. in 2017 *IEEE Int. Ultrason. Symp.* 1-4 (IEEE).

223 Leclerc, S. *et al.* Deep Learning for Segmentation Using an Open Large-Scale Dataset in 2D Echocardiography. *IEEE Trans. Med. Imaging* **38**, 2198-2210 (2019).

224 Chen, C. *et al.* Deep Learning for Cardiac Image Segmentation: A Review. *Front. Cardiovasc. Med.* **7**, 25 (2020).

225 Yamashita, R., Nishio, M., Do, R. K. G. & Togashi, K. Convolutional neural networks: an overview and application in radiology. *Insights into Imaging* **9**, 611-629 (2018).

226 Zhou, S. K., Le, H. N., Luu, K., H, V. N. & Ayache, N. Deep reinforcement learning in medical imaging: A literature review. *Med. Image Anal.* **73**, 102193 (2021).

227 Sun, L. *et al.* Few-shot medical image segmentation using a global correlation network with discriminative embedding. *Comput. Biol. Med.* **140**, 105067 (2022).

228 Shattuck, D. P., Weinshenker, M. D., Smith, S. W. & von Ramm, O. T. Explososcan: a parallel processing technique for high speed ultrasound imaging with linear phased arrays. *J. Acoust. Soc. Am.* **75**, 1273-1282 (1984).

229 https://multimedia.3m.com/mws/media/66235O/3m-electrically-conductive-adhesive-

transfer-tape-9703.pdf

230 https://www.3m.com/3M/en_US/p/d/b00036780/

231 https://multimedia.3m.com/mws/media/66127O/3m-xyz-axis-electrically-conductive-tape-9713.pdf

232 https://www.3m.com/3M/en_US/p/d/b10179639/

233 https://multimedia.3m.com/mws/media/37370O/3m-emi-copper-foil-shielding-tape-1181-data-sheet-78-8127-9953-0-b.pdf

234 https://www.3m.com/3M/en_US/p/d/b40067945/

235 https://github.com/divamgupta/image-segmentation-keras

236 https://github.com/divamgupta/image-segmentation-keras

237 https://github.com/divamgupta/image-segmentation-keras

238 https://github.com/LeeJunHyun/Image_Segmentation

239 https://github.com/MrGiovanni/UNetPlusPlus

240 https://github.com/divamgupta/image-segmentation-keras

241 https://github.com/divamgupta/image-segmentation-keras

242 https://github.com/divamgupta/image-segmentation-keras
